# Terpenoids from *Glechoma hederacea* var. *longituba* and their biological activities

**DOI:** 10.3762/bjoc.18.58

**Published:** 2022-05-17

**Authors:** Dong Hyun Kim, Song Lim Ham, Zahra Khan, Sun Yeou Kim, Sang Un Choi, Chung Sub Kim, Kang Ro Lee

**Affiliations:** 1 School of Pharmacy, Sungkyunkwan University, Suwon 16419, Republic of Korea; 2 Department of Biopharmaceutical Convergence, Sungkyunkwan University, Suwon 16419, Republic of Korea; 3 College of Pharmacy, Gachon University #191, Hambakmeoro, Yeonsu-gu, Incheon 21936, Republic of Korea; 4 Gachon Institute of Pharmaceutical Science, Gachon University, Incheon 21936, Republic of Korea; 5 Korea Research Institute of Chemical Technology, Daejeon 34114, Republic of Korea

**Keywords:** antineuroinflammation, cytotoxicity, *Glechoma hederacea* var. *longituba*, neurotrophic effect, terpenoid

## Abstract

*Glechoma hederacea* var. *longituba* (common name: ground ivy) has been used for the treatment of asthma, bronchitis, cholelithiasis, colds, and inflammation. In the present study, three new sesquiterpene glycosides (**1**–**3**), two new diterpene glycosides (**4** and **5**), and four known compounds (**6**–**9**) were isolated from its MeOH extract. A structure elucidation was performed for the five new compounds (**1**–**5**) using 1D and 2D NMR, HRESIMS, DP4+ and ECD calculations, and chemical methods. All the isolates (**1**–**9**) were assessed for their antineuroinflammatory activities on nitric oxide (NO) production in lipopolysaccharide (LPS)-activated BV-2 cells, nerve growth factor (NGF) secretion stimulation activities in C6 glioma cells, and cytotoxic activities against four human cancer cell lines (A549, SK-OV-3, SK-MEL-2, and HCT15). Compounds **2** and **5**–**7** exhibited inhibitory effects on the NO production with IC_50_ values of 52.21, 47.90, 61.61, and 25.35 μM, respectively. Compound **5** also exhibited a significant stimulating effect on NGF secretion (122.77 ± 8.10%). Compound **9** showed potent cytotoxic activity against SK-OV-3 (IC_50_ = 3.76 μM) and SK-MEL-2 (IC_50_ = 1.48 μM) cell lines, while **7** displayed a strong cytotoxic activity against the SK-MEL-2 (IC_50_ = 9.81 μM) cell line.

## Introduction

**Figure 1:**
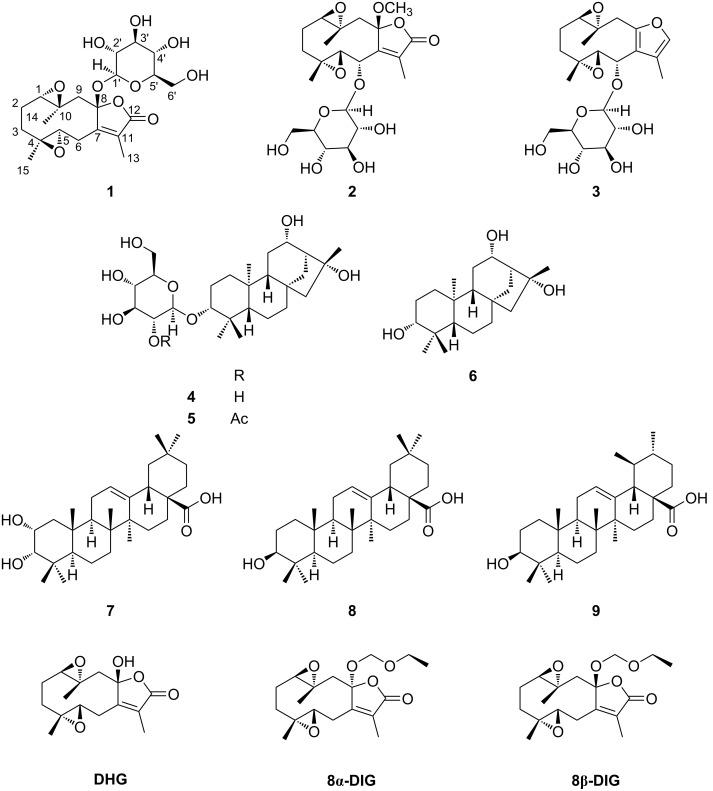
Chemical structures of compounds **1–9**.

*Glechoma hederacea* var. *longituba* is a perennial plant in the family Labiatae. It is commonly known as ‘ground ivy’ and ‘gill over the ground’ and is widely distributed in Korea, Japan, and China. This plant has been used as Korean traditional medicine for treating asthma, bronchitis, cholelithiasis, colds, and inflammation [1–2]. Previous studies have shown that *G. hederacea* var. *longituba* contains phytochemicals such as monoterpenoids, sesquiterpene lactones, lignans, flavonoids, and phenolic compounds that show anti-inflammatory, cytotoxic, and/or cytoprotective effects [3–7]. However, bioactive terpenoids of *G. hederacea* var. *longituba* with antineurodegenerative effects remain largely unknown. In this study, nine terpenoids (**1**–**9**) including five new compounds (**1**–**5**) were isolated and characterized from *G. hederacea* var. *longituba* (Figure 1). Structures of these compounds were established by 1D and 2D NMR and HRESIMS, comparison of experimental and calculated ECD data, DP4+ analysis, and hydrolysis. Herein, the isolation and structural elucidation of the isolated compounds (**1**–**9**) and assessment for their antineuroinflammatory activity on NO production in lipopolysaccharide (LPS)-activated BV-2 cells, NGF secretion-stimulation activities in C6 glioma cells, and cytotoxic activities are described.

## Results and Discussion

**Figure 2:**
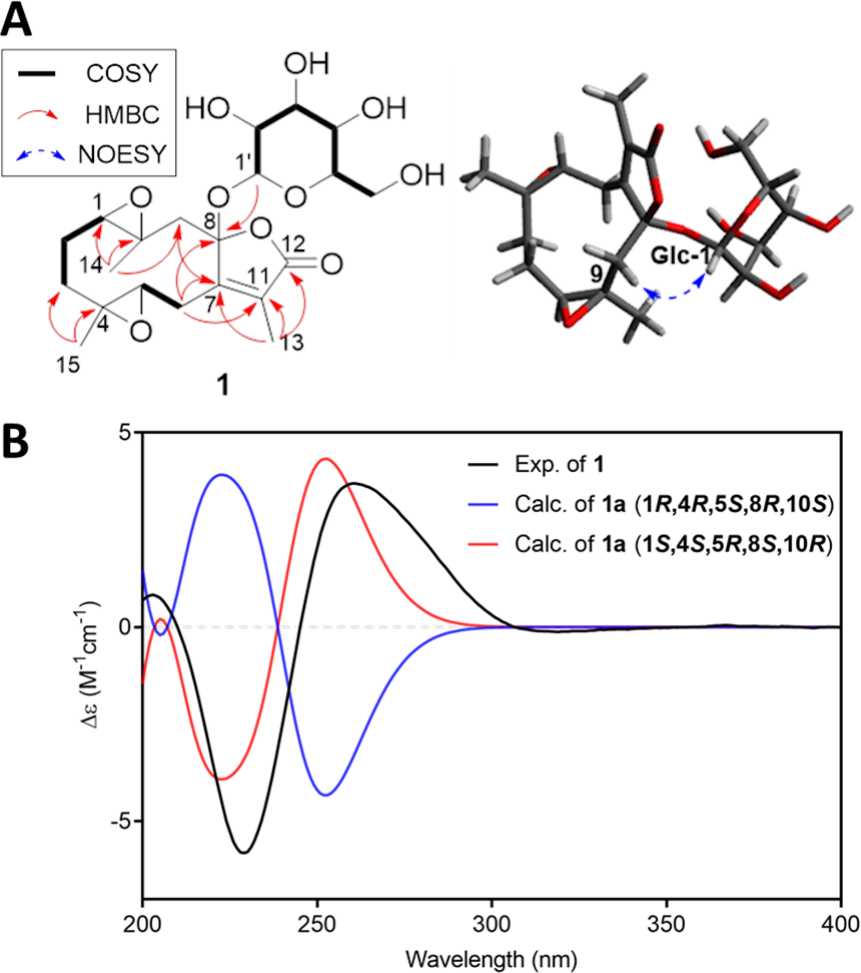
Structure elucidation of **1**. (A) Key COSY, HMBC, and NOE correlations of **1**. (B) Comparison of calculated ECD data of **1a** and experimental ECD spectrum of **1**.

Compound **1** was purified as a colorless gum with a molecular formula of C_21_H_30_O_10_ (*m*/*z* 465.1737 [M + Na]^+^, calcd for C_21_H_30_O_10_Na, 465.1737). The ^1^H NMR spectrum of **1** displayed three methyl groups [δ_H_ 1.89 (3H, d, *J* = 1.4 Hz), 1.28 (3H, s), and 1.13 (3H, s)], two oxygenated methines [δ_H_ 3.45 (1H, dd, *J* = 8.8, 5.9 Hz), and 2.99 (1H, dd, *J* = 10.9, 1.1 Hz)], and a glucopyranosyl unit [δ_H_ 4.20 (1H, d, *J* = 7.8 Hz), 3.78 (1H, dd, *J* = 11.9, 2.2 Hz), 3.59 (1H, dd, *J* = 11.9, 5.8 Hz), 3.31 (1H, overlap), 3.26 (1H, dd, *J* = 9.6, 9.0 Hz), 3.23 (1H, dd, *J* = 9.2, 7.8 Hz), and 3.05 (1H, ddd, *J* = 9.6, 5.8, 2.2 Hz)]. The ^13^C NMR spectrum of **1** showed 21 carbon signals, including four oxygenated carbons (δ_C_ 70.0, 61.4, 60.5, and 57.9), a lactone moiety (δ_C_ 172.6, 159.0, 131.2, and 109.2), and a glucopyranosyl moiety (δ_C_ 97.2, 78.7, 78.1, 74.5, 71.4, and 62.5) (Table 1). Comparison of these ^1^H and ^13^C NMR spectra of **1** with those of 1β,10α;4α,5β-diepoxy-8β-hydroxy-glechoman-8,12-olide (DHG) indicated that **1** could be the glucopyranosyl-DHG [8]. The HMBC cross peak from H-1' (δ_H_ 4.20) to C-8 (δ_C_ 109.2) suggested a glucopyranosyl unit located at C-8 (Figure 2A). The relative configuration of **1** was established based on comparison of ^1^H NMR data with the reported literature and NOESY data analysis. Comparison with ^1^H NMR of 1β,10α;4α,5β-diepoxy-8α-isobutoxy-glechomanolide (8α-DIG) and 1β,10α;4α,5β-diepoxy-8β-isobutoxy-glechomanolide (8β-DIG) of reference [8], the two epimers similar to **1** showed a significant difference in the chemical shift of H-5 (8α, δ_H_ 3.24; 8β, δ_H_ 2.69), H-13 (8α, δ_H_ 1.90; 8β, δ_H_ 2.05), and coupling constants of H-1 (8α, ^3^*J*_H-1/H-2_ = 10, 1.5 Hz; 8β, ^3^*J*_H-1/H-2_ = 4, 1 Hz). The ^1^H NMR chemical shifts and coupling constants of compound **1** (δ_H_ 3.45 and 1.89, ^3^*J*_H-1/H-2_ = 10.1, 1.1 Hz) is similar to that of 8α-DIG, suggesting that **1** could be the same orientation as 8α-DIG. Also, the NOESY spectrum of **1** showed cross peaks between H_3_-14, H-3a/H-2a; H-3a, H-6a/H_3_-15 indicating their α-orientation, whereas the NOESY cross peaks of H-1, H-1'/H-9b; H-3b, H-6b/H-5 suggested their β-orientation (Figure 2A). The absolute configuration of **1** was implied based on comparison of the calculated ECD spectra of **1a** (aglycone of **1**) and experimental ECD spectra of **1** (Figure 2B). The experimental ECD spectrum of **1** showed a negative Cotton effect at 230 nm and a positive Cotton effect at 260 nm, which showed a similarity with those of the calculated ECD spectrum of 1*S*,4*S*,5*R*,8*S*,10*R*-(**1a**). Finally, the ᴅ-glucopyranosyl moiety was identified by GC–MS analysis of a chiral derivatization product of the sugar obtained by enzyme hydrolysis of **1** [9–10]. The retention time of glucopyranose (11.3 min) corresponded to that of the standard ᴅ-glucopyranose (11.3 min) (Supporting Information File 1, Figure S8), and the coupling constant of the anomeric proton (*J* = 7.8 Hz), confirmed it as being in the β-configuration [11]. Thus, the structure of **1** was determined as (1*S*,4*S*,5*R*,8*S*,10*R*)-1,10;4,5-diepoxy-8-*O*-β-ᴅ-glucopyranosyl-glechoman-8,12-olide.

**Table 1:** ^1^H (700 MHz) and ^13^C (175 MHz) NMR data of compounds **1** and **2** in methanol-*d*_4_.

Position	**1**		**2**	

	δ_C_	δ_H_ multi (*J* in Hz)	δ_C_	δ_H_ multi (*J* in Hz)

1	70.0	2.99 dd (10.9, 1.1)	70.7	2.98 d (10.1)
2a	24.2	1.49 m	23.7	1.98 dt (13.9, 3.3)
2b		2.00 dt (14.2, 3.3)		1.50 m
3a	37.8	2.23 dt (13.3, 3.3)	38.2	1.41 td (13.4, 4.1)
3b		1.35 td (13.6, 4.0)		2.25 dt (13.2, 3.3)
4	61.4		62.3	
5	60.5	3.45 dd (8.8, 5.9)	65.7	3.52 d (7.5)
6a	29.2	2.56 dd (15.6, 8.9)	76.6	4.79 d (7.5)
6b		3.53 ddd (15.5, 5.7, 1.3)		
7	159.0		151.4	
8	109.2		110.9	
9a	46.8	3.01 d (15.2)	45.3	2.08 d (15.0)
9b		2.16 d (15.2)		3.01 d (15.0)
10	57.9		57.9	
11	131.2		136.9	
12	172.6		171.9	
13	9.8	1.89 d (1.4)	10.2	2.01 s
14	17.6	1.13 s	17.2	1.08 s
15	17.0	1.28 s	17.7	1.29 s
1'	97.2	4.20 d (7.8)	103.1	4.42 d (7.7)
2'	74.5	3.23 dd (9.2, 7.8)	75.4	3.27 overlap
3'	78.1	3.31 overlap	78.4	3.37 overlap
4'	71.4	3.26 dd (9.6, 9.0)	71.9	3.27 overlap
5'	78.7	3.05 ddd (9.6, 5.8, 2.2)	78.6	3.32 overlap
6'a	62.5	3.78 dd (11.9, 2.2)	63.3	3.90 dd (11.9, 2.3)
6'b		3.59 dd (11.9, 5.8)		3.64 dd (11.9, 6.8)
8-OCH_3_			51.3	3.29 s

**Figure 3:**
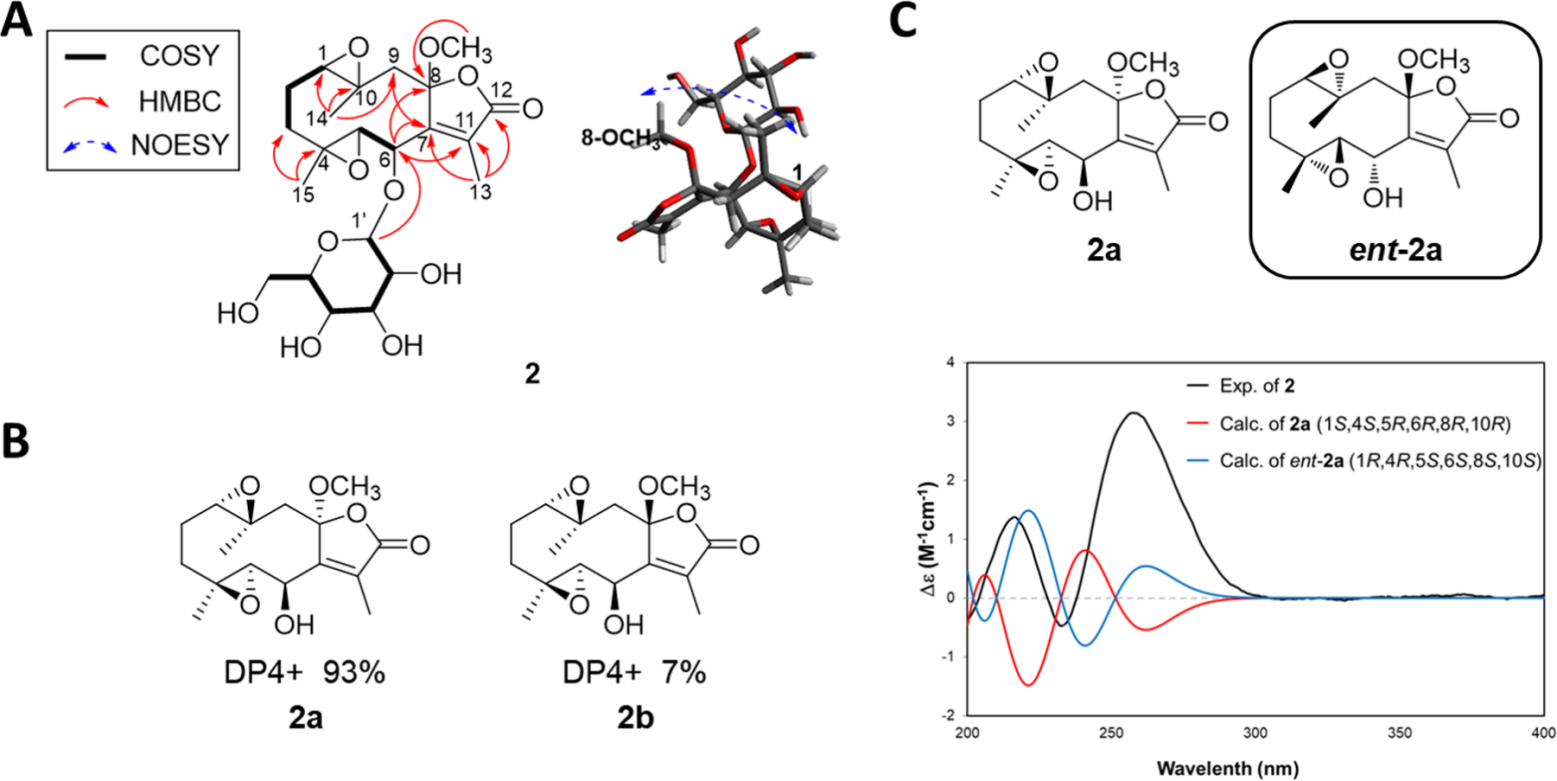
Structure elucidation of **2**. (A) Key COSY, HMBC, and NOE correlations of **2**. (B) DP4+ analysis results for **2a** and **2b**. (C) Comparison of calculated ECD data of **2a** with UV corrections and experimental ECD spectrum of **2**.

Compound **2** was obtained as a colorless gum. Its molecular formula C_22_H_32_O_11_ was determined based on HRESIMS data (*m/z* 495.1839 [M + Na]^+^, calcd for C_22_H_32_O_11_Na, 495.1842). The ^1^H NMR spectrum of **2** displayed three methyl groups [δ_H_ 2.01 (3H, s), 1.29 (3H, s), and 1.08 (3H, s)], three oxygenated methines [δ_H_ 4.79 (1H, d, *J* = 7.5 Hz), 3.52 (1H, d, *J* = 7.5 Hz), and 2.98 (1H, d, *J* = 10.1 Hz)], a methoxy group [δ_H_ 3.29 (3H, s)] and a glucopyranosyl unit [δ_H_ 4.42 (1H, d, *J* = 7.7 Hz), 3.90 (1H, dd, *J* = 11.9, 2.3 Hz), 3.64 (1H, dd, *J* = 11.9, 6.8 Hz), 3.37 (1H, overlap), 3.32 (1H, overlap), 3.27 (1H, overlap), and 3.27 (1H, overlap)]. The ^13^C NMR spectrum of **2** showed 22 carbon signals including five oxygenated carbons (δ_C_ 76.6, 70.7, 65.7, 62.3, and 57.9), a lactone moiety (δ_C_ 171.9, 151.4, 136.9, and 110.9), a methoxy carbon (δ_C_ 51.3), and a glucopyranosyl unit (δ_C_ 103.1, 78.6, 78.4, 75.4, 71.9, and 63.3) (Table 1). These NMR spectra of **2** were similar to those of substolide A except for signals of the methoxy group attached to C-8 and of a monosaccharide at C-6 in **2** [12]. The planar structure of **2** was established based on 2D NMR spectroscopic data (COSY, HSQC, and HMBC). The HMBC correlation from H-1' (δ_H_ 4.42) to C-6 (δ_C_ 76.6) suggested a glucopyranosyl unit located at C-6. Also, the HMBC correlations from 8-OCH_3_ (δ_H_ 3.29) to C-8 (δ_C_ 110.9) indicated that an additional methoxy group was located at C-8 (Figure 3). The relative configuration of **2** was established based on NOESY data and DP4+ analysis. The NOE correlations of H_3_-14, H-3b/H-2b; H-6/H-3b/H_3_-15 suggested that H-6, H_3_-14, and H_3_-15 were positioned in the same orientation. To confirm the relative configuration of C-8 in **2** was determined using a DP4+ statistical analysis [13]. The DP4+ protocol was applied to the simulated ^13^C NMR chemical shifts of the conformer **2a** and **2b** (Figure 3B). The DP4+ statistical analysis supported the structural equivalence of **2** to **2a (**α-configuration at C-8) with 93% probability (Figure 3B). The absolute configuration of **2** was implied based on a comparison of the calculated ECD spectra of **2a** and experimental ECD spectra of **2** (Figure 3C). The experimental ECD spectrum of **2** showed a negative Cotton effect at 233 nm and positive Cotton effects at 217 and 257 nm, which showed a similarity with those of calculated ECD spectrum of 1*R*,4*R*,5*S*,6*S*,8*S*,10*S*-(*ent*-**2a**). Enzyme hydrolysis and following sugar identification were performed using the same methods as those used for **1**. As a result, the monosaccharide of **2** was identified as ᴅ-glucose (Figure S17, Supporting Information File 1). The anomeric proton configuration of the glucopyranosyl unit was determined to be β-configuration based on the *J* value (7.7 Hz) [11]. Thus, the structure of **2** was established as (1*R*,4*R*,5*S*,6*S*,8*S*,10*S*)-1,10;4,5-diepoxy-6-*O*-β-ᴅ-glucopyranosyl-8-methoxy-glechoman-8,12-olide.

**Figure 4:**
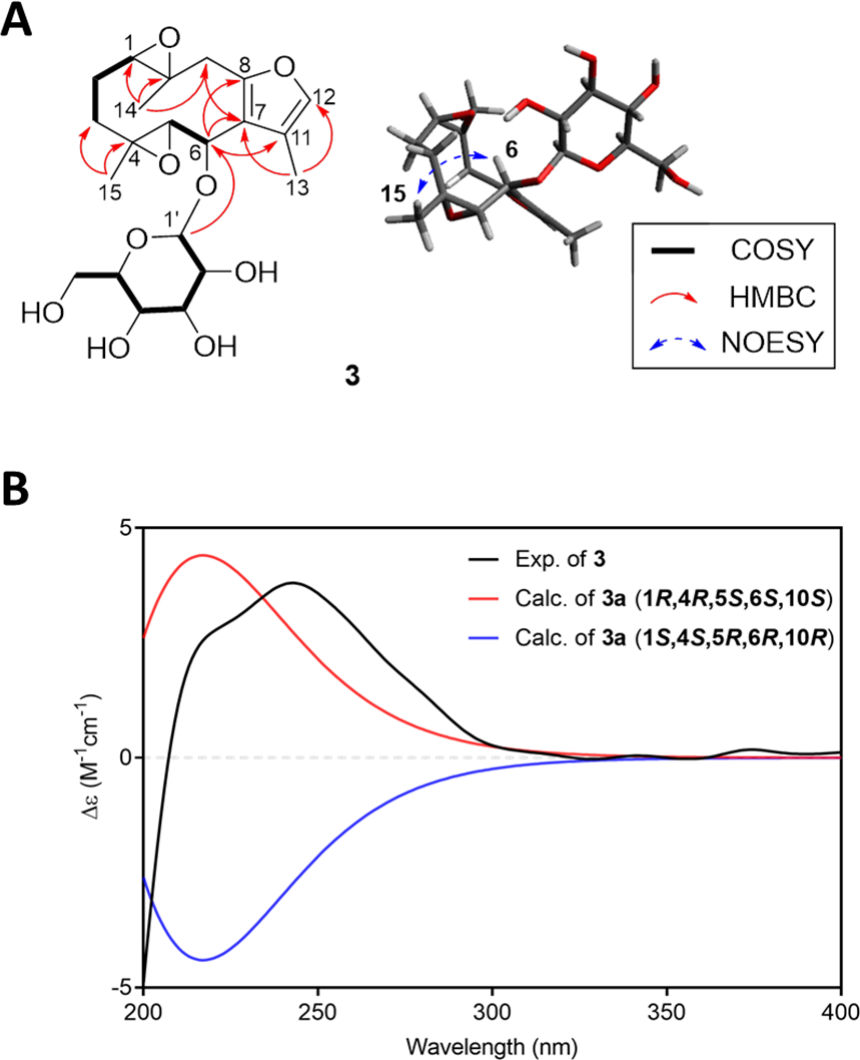
Structure elucidation of **3**. (A) Key COSY, HMBC, and NOE correlations of **3**. (B) Comparison of calculated and experimental ECD spectra of **3**.

Compound **3** was obtained as a colorless gum. The HRESIMS spectrum of **3** provided a molecular formula C_21_H_30_O_9_ (*m*/*z* 449.1789 [M + Na]^+^, calcd for C_21_H_30_O_9_Na, 449.1788). The ^1^H NMR data of **3** exhibited three methyl groups [δ_H_ 2.02 (3H, d, *J* = 1.2 Hz), 1.06 (3H, s), and 0.95 (3H, s)], three oxygenated methine [δ_H_ 4.62 (1H, d, *J* = 6.0 Hz), 3.15 (1H, d, *J* = 10.4 Hz), and 3.67 (1H, overlap)], an olefinic proton [δ_H_ 7.25 (1H, d, *J* = 1.2 Hz)], and a glucopyranosyl unit [δ_H_ 4.23 (1H, d, *J* = 7.8 Hz), 3.88 (1H, dd, *J* = 11.9, 2.3 Hz), 3.67 (1H, dd, *J* = 11.9, 6.1 Hz), 3.29 (1H, overlap), 3.28 (1H, overlap), 3.24 (1H, overlap), and 3.21 (1H, overlap)]. The ^13^C NMR spectrum of **3** showed 21 carbon signals for five oxygenated carbons (δ_C_ 74.5, 68.7 (×2), 61.6, and 61.4), a furan moiety (δ_C_ 151.2, 138.7, 124.5, and 119.6), and a glucopyranosyl unit (δ_C_ 101.7, 78.2 (×2), 75.3, 72.0, and 63.2) (Table 2). These NMR spectra of **3** were similar to those of glechomafuran, except for the signals assignable to H-6/C-6 (δ_H_ 4.62/ δ_C_ 74.5) and those indicative of the presence of a glucopyranosy residue in **3** [14]. The COSY correlation between H-5 (δ_H_ 3.67) and H-6 (δ_H_ 4.62) and the HMBC cross peak of H-1' (δ_H_ 4.23) to C-6 (δ_C_ 74.5) and between H-6 and C-7 (δ_C_ 119.6) confirmed the location of the glucopyranosyl group at C-6 (Figure 4A). The anomeric carbon configuration for a glucopyranosyl unit was defined as β-configuration from the coupling constant of 7.8 Hz [11]. The relative configuration of **3** was established using the NOESY spectrum (Figure 4A). The NOE correlations of H_3_-14, H-3b/H-2b; H-6/H-3b/H_3_-15 suggested that H-6, H_3_-14, and H_3_-15 were positioned in the same orientation. The absolute configuration of **3** was confirmed by comparing the calculated ECD spectrum of **3a** (aglycone of **3**) with the experimental ECD spectrum of **3**. The experimental ECD of **3** displayed positive Cotton effects at 217 and 243 nm, which was similar to those of 1*R*,4*R*,5*S*,6*S*,10*S*-(**3a**) (Figure 4B). Enzyme hydrolysis and sugar identification were performed and monosaccharide of **3** was identified as ᴅ-glucopyranose (Supporting Information File 1, Figure S26). Thus, the structure of **3** was determined as (1*R*,4*R*,5*S*, 6*S*,*10S*)-1,10;4,5-diepoxy-6-*O*-β-ᴅ-glucopyranosyl-glechomafuran.

**Table 2:** ^1^H (700 MHz) and ^13^C (175 MHz) NMR data of compound **3** in methanol-*d*_4_.

Position	**3**	

	δ_C_	δ_H_ multi (*J* in Hz)

1	68.7	3.15 d (10.4)
2a	23.6	1.99 m
2b		1.49 m
3a	38.5	1.41 td (13.3, 5.0)
3b		2.26 ddd (13.3, 3.9, 2.7)
4	61.6	
5	68.7	3.67 overlap
6	74.5	4.62 d (6.0)
7	119.6	
8	151.2	
9a	37.8	2.80 d (14.3)
9b		3.19 m
10	61.4	
11	124.5	
12	138.7	7.25 d (1.2)
13	8.4	2.02 d (1.2)
14	18.1	0.95 s
15	17.3	1.06 s
1'	101.7	4.23 d (7.8)
2'	75.3	3.21 overlap
3'	78.2	3.29 overlap
4'	72.0	3.28 overlap
5'	78.2	3.24 overlap
6'a	63.2	3.88 dd (11.9, 2.3)
6'b		3.67 dd (11.9, 6.1)

**Figure 5:**
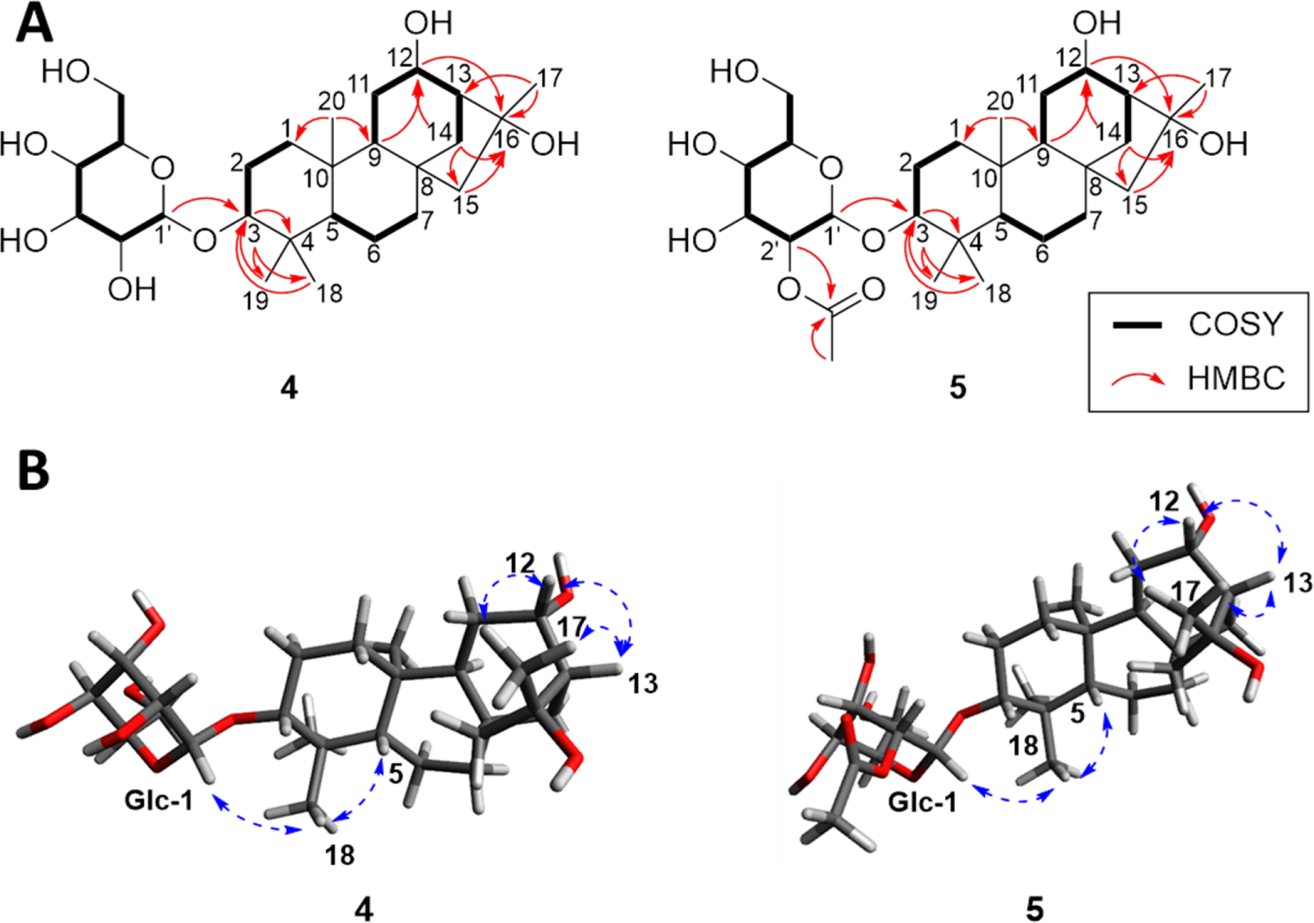
2D NMR data of **4** and **5**. (A) Key COSY and HMBC correlations of **4** and **5**. (B) Key NOE correlations of **4** and **5**.

Compound **4** was purified as a colorless gum. The HRESIMS spectrum of **4** gave a molecular formula of C_26_H_44_O_8_ according to a [M + Na]^+^ ion at *m*/*z* 507.2935 (calcd for C_26_H_44_O_8_Na, 507.2934). The ^1^H NMR spectrum of **4** displayed four methyl groups [δ_H_ 1.46 (3H, s), 1.06 (3H, s), 1.04 (3H, s), and 0.83 (3H, s)], two oxygenated methines [δ_H_ 3.97 (1H, ddd, *J* = 11.8, 6.7, 3.6 Hz) and 3.33 (1H, overlap)], and a glucopyranosyl unit [δ_H_ 4.33 (1H, d, *J* = 7.8 Hz), 3.86 (1H, dd, *J* = 11.7, 2.4 Hz), 3.68 (1H, dd, *J* = 11.7, 5.7 Hz), 3.37 (1H, t, *J* = 9.0 Hz), 3.31 (1H, overlap), 3.23 (1H, ddd, *J* = 9.6, 5.7, 2.4 Hz), and 3.18 (1H, dd, *J* = 9.1, 7.8 Hz)]. The ^13^C NMR spectrum of **4** displayed 26 carbon signals including two oxygenated carbons (δ_C_ 85.8 and 72.4), an oxygenated quaternary carbon (δ_C_ 79.5), and a glucopyranosyl unit (δ_C_ 101.9, 78.3, 77.7, 75.2, 71.9, and 63.0) (Table 3). These ^1^H and ^13^C NMR spectra of **4** were similar to those of *ent*-kauran-3α,12α,16α-triol except for an additional glucopyranosyl unit [15]. The location of glucopyranosyl unit at C-3 was confirmed by a HMBC cross peak from H-1' (δ_H_ 4.33) to C-3 (δ_C_ 85.8), as well as by the deshielding and shielding effects of the glucopyranosyl substituent on C-3 and C-2, respectively, whose resonances were found at δ_C_ 79.3 and 27.7, respectively, in the corresponding aglycone (Figure 5A). The relative configuration of **4** was established based on NOESY data and comparison of NMR, and optical rotation value with reported literature. The NOE correlations of H-3, H-5/H_3_-18; H-5/H-9; H-9, H-12, H_3_-17/H-13 suggested that H-3, H-5, H-9, H-12, H-13, H_3_-17, and H_3_-18 were positioned in the same orientation (Figure 5B). Also, ^1^H and ^13^C NMR except for those signals attributed to a glucopyranosyl unit, and optical rotation value of aglycone {[α] −66.5 (*c* 0.02, MeOH)} were compared with the reported literature and determined as 3α,12α,16α [15]. Finally, the ᴅ-glucopyranose unit in **4** was confirmed by GC–MS analysis as described above (Supporting Information File 1, Figure S35) [10], and the coupling constant of anomeric protons at δ_H_ 4.33 (*J* = 7.8 Hz) implied β-ᴅ-glucopyranose [16]. Thus, the structure of compound **4** was identified as *ent*-kauran-3α,12α,16α-triol 3-O-β-ᴅ-glucopyranoside.

**Table 3:** ^1^H (700 MHz) and ^13^C (175 MHz) NMR data of compounds **4** and **5** in methanol-*d*_4_.

Position	**4**		**5**	

	δ_C_	δ_H_ multi (*J* in Hz)	δ_C_	δ_H_ multi (*J* in Hz)

1	39.7	1.90, 0.90 m	39.8	1.91, 0.89 m
2	24.1	1.79, 1.68 m	24.0	1.77, 1.48 m
3	85.8	3.33, overlap	85.5	3.31, overlap
4	39.2		39.3	
5	56.9	0.84 dd (11.9, 1.3)	56.8	0.82 dd (11.7, 1.2)
6	21.2	1.59, 1.37 m	21.2	1.59, 1.36 m
7	42.1	1.67, 1.50 m	42.2	1.68, 1.49 m
8	45.7		45.8	
9	59.0	1.10 d (8.9) m	59.0	1.10 d (8.8) m
10	39.7		39.6	
11	28.7	1.87, 1.56 m	28.8	1.86, 1.57 m
12	72.4	3.97 ddd (11.8, 6.7, 3.6)	72.4	3.98 ddd (11.8, 6.7, 3.6)
13	56.0	2.02 m	56.1	2.02 t (4.0)
14	38.4	1.83, 1.71 m	38.5	1.83, 1.72 m
15	59.3	1.57, 1.54 m	59.4	1.56 m
16	79.5		79.6	
17	26.3	1.46 s	26.4	1.47 s
18	29.0	1.04 s	29.0	1.01 s
19	17.1	0.83 s	16.9	0.73 s
20	18.4	1.06 s	18.5	1.04 s
1'	101.9	4.33 d (7.8)	99.5	4.52 d (8.0)
2'	75.2	3.18 dd (9.1, 7.8)	75.7	4.69 dd (9.6, 8.0)
3'	78.3	3.37 t (9.0)	76.3	3.54 t (9.3)
4'	71.9	3.31 overlap	72.0	3.39 t (9.3)
5'	77.7	3.23 ddd (9.6, 5.7, 2.4)	78.5	3.28 ddd (9.7, 5.7, 2.2)
6'a	63.0	3.86 dd (11.7, 2.4)	62.9	3.88 dd (11.8, 2.2)
6'b		3.68 dd (11.7, 5.7)		3.71 dd (11.8, 5.7)
2'-OAc			172.0	
			21.2	2.03 s

Compound **5** was obtained as a colorless gum. Based on its HRESIMS and NMR data, its molecular formula was determined as C_28_H_46_O_9_ (*m*/*z* 549.3031 [M + Na]^+^, calcd for C_28_H_46_O_9_Na, 549.3040). The ^1^H NMR spectrum of **5** showed four methyl groups [δ_H_ 1.47 (3H, s), 1.04 (3H, s), 1.01 (3H, s), and 0.73 (3H, s)], two oxygenated methines [δ_H_ 3.98 (1H, ddd, *J* = 11.8, 6.7, 3.6 Hz) and 3.31 (1H, overlap)], and a (2'-*O*-acetyl)glucopyranosyl unit [δ_H_ 4.69 (1H, dd, *J* = 9.6, 8.0 Hz), 4.52 (1H, d, *J* = 8.0 Hz), 3.88 (1H, dd, *J* = 11.8, 2.2 Hz), 3.71 (1H, dd, *J* = 11.8, 5.7 Hz), 3.54 (1H, t, *J* = 9.3 Hz), 3.39 (1H, t, *J* = 9.3 Hz), and 3.28 (1H, ddd, *J* = 9.7, 5.7, 2.2 Hz)]. The ^13^C NMR spectrum of **5** displayed 28 carbon signals including two oxygenated carbons (δ_C_ 85.5 and 72.4), an oxygenated quaternary carbon (δ_C_ 79.6), a glucopyranosyl unit (δ_C_ 99.5, 75.7, 76.3, 72.0, 78.5, and 62.9), and an acetyl group carbon (δ_C_ 172.0 and 21.2) (Table 3). The ^1^H and ^13^C NMR spectra of compound **5** were similar to those of compound **4** except for the presence of acetyl group signals. The HMBC correlation from H-2' (δ_H_ 4.69) to the carbonyl carbon at δ_C_ 172.0 (C-2'') located the acetyl group at C-2' (Figure 5A). Moreover, by the shielding effect of the acetyl group on C-1' and C-3', whose chemical shifts were observed at δ_C_ 99.5 and 76.3, respectively. The relative configuration of **5** was determined as being the same as that of **4** based on NOESY data (Figure 5B) and comparison of NMR, and optical rotation value {[α] −68.5 (*c* 0.02, MeOH)}. Finally, ᴅ-glucopyranoside was confirmed by LC–MS analysis for monosaccharide derivatives obtained by derivatization reaction after acid hydrolysis of **5** [17]. The retention time of glucopyranose (13.8 min) corresponded with standard ᴅ-Glc (13.8 min) (Supporting Information File 1, Figure S43). Thus, the structure of compound **5** was established as *ent*-kauran-3α,12α,16α-triol 3-O-β-ᴅ-(2'-*O*-acetyl)-glucopyranoside.

By comparing the NMR and MS data with those reported in literature, the four known compounds **6**–**9** were identified as *ent*-kauran-3α,12α,16α-triol (**6**) [15], 3-epimaslinic acid (**7**) [18], oleanolic acid (**8**) [19], and ursolic acid (**9**) [20].

To find potential antineuroinflammatory, neurotrophic, and cytotoxic secondary metabolites from *G. hederacea* var. *longituba*, the isolated compounds (**1**–**9**) were evaluated for these biological activities. The antineuroinflammatory activity of all isolates (**1**–**9**) was assessed by measuring nitric oxide (NO) production levels in LPS-stimulated murine microglia BV-2 cells (Table 4). Compounds **2** and **5**–**7** significantly reduced NO levels, with IC_50_ values of 52.21, 47.90, 61.61, and 25.35 μM, respectively. Interestingly, of the two diterpene glucosides (**4** and **5**) that were structurally similar except for the C-2' functionality (**4**, -OH; **5**, -OAc) of the glucopyranosyl group, only compound **5** showed NO inhibitory activity. This suggests that the presence of an acetyl group at C-2' might play an important role in the NO inhibitory activity.

**Table 4:** Inhibitory effects of compounds **1**–**9** on NO production in LPS-activated BV-2 cells.

Compound	IC_50_ (μM)^a^	Cell viability (%)^b^

**1**	119.48	71.22 ± 2.49
**2**	52.21	68.86 ± 3.27
**3**	292.32	97.02 ± 5.57
**4**	>500	103.11 ± 4.17
**5**	47.90	106.26 ± 3.71
**6**	61.61	95.29 ± 5.25
**7**	25.35	104.88 ± 4.74
**8**	>500	101.17 ± 3.89
**9**	104.25	97.34 ± 3.97
L-NMMA^c^	55.75	97.84 ± 2.05

^a^IC_50_ value of each compound was defined as the concentration (μM) that caused 50% inhibition of NO production in LPS-activated BV-2 cells. ^b^Cell viability after treatment with 10 μM of each compound was determined by MTT assay and is expressed in percentage (%). The results are averages of three independent experiments, and the data are expressed as mean ± SD. ^c^ʟ-NMMA as positive control.

Compounds **1**–**9** were also tested for their abilities to stimulate the NGF secretion in C6 glioma cells (Table 5). Compound **5** exhibited a significant stimulating effect on the secretion of NGF (122.77 ± 8.10%), whereas other compounds did not. Similar to results of their NO inhibitory activities, compound **5** showed a stronger stimulating effect on the secretion of NGF than compound **4** (76.56 ± 6.51%).

**Table 5:** Effects of compounds **1**–**9** on NGF secretion in C6 cells.

Compound	NGF secretion^a^	Cell viability (%)^b^

**1**	82.87 ± 8.63	93.47 ± 9.87
**2**	97.01 ± 11.40	103.47 ± 2.80
**3**	80.06 ± 3.46	101.18 ± 5.56
**4**	76.56 ± 6.51	85.46 ± 11.23
**5**	122.77 ± 8.10	125.51 ± 2.37
**6**	85.51 ± 6.68	119.35 ± 11.92
**7**	76.50 ± 7.21	121.92 ± 7.43
**8**	88.40 ± 9.04	120.92 ± 6.78
**9**	87.55 ± 6.62	100.33 ± 9.68
6-shogaol^c^	116.85 ± 14.91	99.22 ± 3.75

^a^C6 cells were treated with 10 μM of compounds. After 24 h, the content of NGF secretion in C6-conditioned media was measured by ELISA. The level of secreted NGF cells is expressed as percentage of the untreated control. The data shown represent the means ± SD of three independent experiments performed in triplicate. ^b^Cell viability after treatment with 10 μM of each compound was determined by MTT assay and is expressed in percentage (%). The results are averages of three independent experiments, and the data are expressed as mean ± SD. ^c^6-Shogaol as positive control.

Cytotoxic effects of compounds **1**–**9** against four human cancer cell lines [non-small-cell lung adenocarcinoma (A549), malignant ovarian ascites (SK-OV-3), skin melanoma (SK-MEL-2), and colon adenocarcinoma (HCT15)] were also investigated using a sulforhodamine B (SRB) bioassay (Table 6). Compound **9** showed selective cytotoxic activities against SK-OV-3 and SK-MEL-2 cell lines, with IC_50_ values of 3.76 and 1.48 μM, respectively. Compound **7** also displayed a cytotoxic activity against SK-MEL-2 cell line, with an IC_50_ value of 9.81 μM.

**Table 6:** Cytotoxic activities of selected compounds against four cultured human cancer cell lines in the SRB bioassay.

Compound	IC_50_ (μM)^a^			

	A549	SK-OV-3	SK-MEL-2	HCT15

**7**	>10	>10	9.81	>10
**9**	>10	3.76	1.48	>10
etoposide^b^	1.21	2.27	2.04	2.38

^a^50% inhibitory concentration; the concentration of compound that caused a 50% inhibition in cell growth. ^b^Etoposide as a positive control.

## Conclusion

Nine terpene derivatives, including three new sesquiterpene glycosides (**1**–**3**), two new diterpene glycosides (**4** and **5**), a known diterpene (**6**), and three known triterpenes (**7**–**9**) were isolated from CHCl_3_-, EtOAc-, and *n*-BuOH-soluble layers of an 80% MeOH extract of *G. hederacea* var. *longituba*. The structures of these compounds were established by 1D and 2D NMR and HRESIMS, comparison of experimental and calculated ECD data, and hydrolysis. All isolated compounds were evaluated for antineuroinflammatory activity, neurotrophic effect, and cytotoxicity. Compounds **2** and **5**–**7** caused significantly reduced NO levels. Compound **5** exhibited a neurotrophic effect. Interestingly, compounds **4** and **5** showed differences in antineuroinflammatory activity and neurotrophic effect according to the C-2' functional group of the glucopyranosyl group. Compounds **7** and **9** showed selective cytotoxic activities against human cancer cell lines. This study suggests that the bioactive tepenoids (**2**, **5–7**, and **9**) from *G. hederacea* var. *longituba* would be potential new drug candidates.

## Experimental

**General experimental procedures.** Optical rotations were measured on a Jasco P-2000 polarimeter using methanol as solvent. High-resolution ESI mass spectrometer data were recorded on a Waters SYNAPT G2 mass spectrometer. ECD spectra were garnered with a JASCO J-1500 CD spectrometer (JASCO, Easton, MD, USA). The NMR spectra were recorded on a Bruker AVANCE III 700 NMR spectrometer at 700 MHz (^1^H) and 175 MHz (^13^C) with solvent resonance as the internal standard (^1^H NMR: CD_3_OD at 3.31 ppm; ^13^C NMR: CD_3_OD at 49.00 ppm). To practice LC–MS analysis, an Agilent 1200 Series high-performance liquid chromatography (HPLC) system furnished with a diode array detector and a 6130 Series ESIMS spectrometer connected to an analytical Kinetex C_18_ column (250 mm × 4.6 mm, 5 µm; Phenomenex, Torrance, CA, USA) was utilized. The Agilent 7820A GC system equipped with a 5977B mass selective detector system was controlled by qualitative navigator version B.08.00 software. Preparative HPLC was performed using a Gilson 321 pump with a Shodex Refractive Index Detector, an INNO column C18 5 μm column (250 × 10 mm), and a Lux 5 μm Cellulose-1 column (250 × 4.6 mm). Silica gel 60 (Merck, 70–230 mesh and 230–400 mesh), RP-C_18_ silica gel (Merck, 230–400 mesh), Sephadex LH-20 (Parmacia Co., Japan), and Diaion HP-20 (Mitsubishi Chemical Co., Japan) were used for column chromatography. Merck precoated silica gel F_254_ plates and reversed-phase (RP)-18 F_254s_ plates (Merck) were used for thin-layer chromatography (TLC). Spots of compounds on TLC were detected under UV light or by heating after dipping in anisaldehyde–sulfuric acid.

**Plant material.** Aerial parts of *G. hederacea* var. *longituba* were purchased from Hongcheon, Gangwon-do, Korea, in May 2016. The plant material was identified by Kang Ro Lee. A voucher specimen of this material (SKKU-NPL-1412) has been deposited in the herbarium of the School of Pharmacy, Sungkyunkwan University, Suwon, Korea.

**Extraction and isolation.** The dried aerial parts of *G. hederacea* var. *longituba* (3.0 kg) were extracted with 80% MeOH (each 12.0 L × 1 day, 3 times) at room temperature and filtered. The filtrate was evaporated in vacuo to yield an 80% MeOH extract (400.0 g). The 80% MeOH extract was suspended in distilled H_2_O (2.4 L) and then successively partitioned with hexanes (2.4 L × 3), CHCl_3_ (2.4 L × 4), EtOAc (2.4 L × 3), and *n*-BuOH (2.4 L × 3) to yield 11.0 g, 16.0 g, 14.0 g, and 37.0 g, respectively. The CHCl_3_ soluble layer (13.0 g) was subjected to a silica gel column (CHCl_3_/MeOH, 20:1 → 1:1) to give five fractions (C1–C5). Fraction C2 (3.0 g) was applied to an RP-C_18_ silica gel column (50% MeOH) to give 12 subfractions (C2a–C2l). Compound **6** (4 mg, *t*_R_ = 25.9 min) was yielded by purifying subfraction C2h (32 mg) using a semipreparative HPLC (65% MeCN). Subfraction C2j (155 mg) was purified by semipreparative HPLC (70% MeCN) to yield compound **7** (3 mg, *t*_R_ = 32.4 min). Compounds **8** (5 mg, *t*_R_ = 23.3 min) and **9** (6 mg, *t*_R_ = 18.9 min) were obtained by purification of subfraction C2k (238 mg) using a semipreparative HPLC (100% MeOH). Fraction C3 (1.3 g) was applied to an RP-C_18_ silica gel column (60% MeOH) to yield nine subfractions (C3a–C3i). Subfraction C3a (266 mg) was fractionated with a silica gel column (CHCl_3_/MeOH, 20:1) to give three subfractions (C3a1–C3a3). C3a1 (62 mg) was purified by semipreparative HPLC (25% MeCN) to obtain compound **5** (3 mg, *t*_R_ = 26.3 min). The EtOAc soluble layer (9.5 g) was chromatographed over a silica column (CHCl_3_/MeOH, 30:1 → 1:1) to give six fractions (E1–E6). Fraction E4 (1.1 g) was applied to an RP-C_18_ silica column (40% MeOH) to yield six subfractions (E4a–E4f). Subfraction E4d (274 mg) was subjected to a silica gel column (CHCl_3_/MeOH/H_2_O, 6:1:0.1) and further purified by semipreparative HPLC (39% MeOH) to obtain compound **4** (4 mg, *t*_R_ = 28.7 min). The *n*-BuOH-soluble layer (34.0 g) was subjected to Diaion HP-20 (H_2_O→MeOH) to yield six fractions (B1–B6). Fraction B4 (4.1 g) was applied to a silica gel column (CHCl_3_/MeOH/H_2_O, 6:1:0.1→1:1:0.1) to give five subfractions (B1a–B1e). Subfraction B1a (484 mg) was chromatographed over an RP-C_18_ silica gel column (40% MeOH) to yield five subfractions (B1a1–B1a5). Subfraction B1a1 was purified by semipreparative HPLC (26% MeCN) to obtain compounds **1** (4 mg, *t*_R_ = 22.1 min) and **2** (5 mg, *t*_R_ = 24.4 min). Subfraction B1a3 (66 mg) was purified by semipreparative HPLC (26% MeCN) to obtain compound **3** (4 mg, *t*_R_ = 28.7 min).

**(1*S*,4*S*,5*R*,8*S*,10*R*)-1,10;4,5-Diepoxy-8-*O*-β-ᴅ-glucopyranosyl-glechoman-8,12-olide (1).** Colorless gum; [α]_D_^25^ +228.0 (*c* 0.1, MeOH); ECD (MeOH) λ_max_ (Δε) 230 (−5.8), 260 (+3.7) nm; ^1^H and ^13^C NMR data, see Table 1; HRESIMS (Supporting Information File 1, Figure S1) *m*/*z*: 465.1737 [M + Na]^+^ (calcd for C_21_H_30_O_10_Na, 465.1737).

**(1*R*,4*R*,5*S*,6*S*,8*S*,10*S*)-1,10;4,5-Diepoxy-6-*O*-β-ᴅ-glucopyranosyl-8-methoxy-glechoman-8,12-olide (2).** Colorless gum; [α]_D_^25^ +24.6 (*c* 0.1, MeOH); ECD (MeOH) λ_max_ (Δε) 217 (+1.4), 233 (−0.5), 257 (+3.1) nm; ^1^H and ^13^C NMR data, see Table 1; HRESIMS (Supporting Information File 1, Figure S10) *m/z*: 495.1839 [M + Na]^+^ (calcd for C_22_H_32_O_11_Na, 495.1842).

**(1*R*,4*R*,5*S*,6*S*,10*S*)-1,10;4,5-Diepoxy-6-*O*-β-ᴅ-glucopyranosyl-glechomafuran (3).** Colorless gum; [α]_D_^25^ –28.0 (*c* 0.1, MeOH); ECD (MeOH) λ_max_ (Δε) 217 (+2.5), 243 (+3.8) nm; ^1^H and ^13^C NMR data, see Table 2; HRESIMS (Supporting Information File 1, Figure S19) *m/z*: 449.1789 [M + Na]^+^ (calcd for C_21_H_30_O_9_Na, 449.1788).

***ent*-Kauran-3α,12α,16α-triol 3-*O*-β-ᴅ-glucopyranoside (4).** Colorless gum; [α]_D_^25^ −94.6 (*c* 0.1, MeOH); ^1^H and ^13^C NMR data, see Table 3; HRESIMS (Supporting Information File 1, Figure S28) *m/z*: 507.2935 [M + Na]^+^ (calcd for C_26_H_44_O_8_Na, 507.2934).

***ent*-Kauran-3α,12α,16α-triol 3-*O*-β-ᴅ-(2'-*O*-acetyl)glucopyranoside (5).** Colorless gum; [α]_D_^25^ −24.6 (*c* 0.1, MeOH); ^1^H and ^13^C NMR data, see Table 3; HRESIMS (Supporting Information File 1, Figure S36) *m/z*: 549.3031 [M + Na]^+^ (calcd for C_28_H_46_O_9_Na, 549.3040).

**Enzyme hydrolysis of compounds 1**–**3.** Compounds **1** (0.8 mg), **2** (0.7 mg), and **3** (0.8 mg) were dissolved in water (2 mL) and then hydrolyzed with β-glucosidase (20 mg, from Almonds, Sigma) at 37 °C for 48 h. The reaction mixture was extracted with CHCl_3_ (3 times), and the aqueous layer was evaporated in vacuo to obtain the monosaccharide (**1**, 0.4 mg, 50%; **2**, 0.3 mg, 43%, **3**, 0.4 mg, 50%).

**Acid hydrolysis of compounds 4 and 5.** Each compound (**4**, 1.0 mg; **5**, 0.8 mg) was hydrolyzed with 1 N HCl (1 mL) under reflux for 1.5 h. The hydrolysate was extracted with CHCl_3_ (3 times), and a monosaccharide residue (**4**, 0.4 mg, 40%; **5**, 0.3 mg, 37%) was obtained from the aqueous layer.

**Sugar analysis using LC–MS.** In a manner similar to [21]. The monosaccharide (**5**, 0.3 mg), obtained by hydrolysis, was dissolved in pyridine (0.5 mL), then ʟ-cysteine methyl ester hydrochloride (2 mg) was added. The reaction mixture was stirred at 60 °C for 1 h. Then *O*-tolyl isothiocyanate (30 μL) was added and stirred at 60 °C for 1 h. The reaction mixture was analyzed without purification by LC–MS analysis. The monosaccharide of **5** was detected at 13.8 min, the same detection time (13.8 min) as ᴅ-glucopyranoside. LC–MS analysis was perfomed under the following conditions: flow rate 0.7 mL/min; 25% MeCN with 0.1% formic acid, column, Kinetex C_18_ column (250 mm × 4.6 mm, 5 µm, Phenomenex).

**Sugar analysis using GC–MS.** Monosaccharides (**1**, 0.4 mg; **2**, 0.3 mg; **3**, 0.4 mg; **4**, 0.4 mg), obtained by hydrolysis, were dissolved in pyridine (0.5 mL), then ʟ-cysteine methyl ester hydrochloride (2 mg) was added. The reaction mixtures were then stirred at 60 °C for 2 h. After adding 1-trimethylsilylimidazole (0.1 mL), the reaction mixture was allowed to react again at 60 °C for 2 h. The reactant was suspended in distilled H_2_O (1 mL) and then partitioned with *n*-hexane (1 mL). The *n*-hexane soluble layer was analyzed using GC–MS analysis. All monosaccharides were detected at 11.3 min, the same detection time (11.3 min) as ᴅ-glucopyranoside. GC–MS analysis was perfomed under the following conditions: capillary column, HP-5MS UI (30 m × 0.25 mm × 0.25 μm, Agilent); column temperature, 230 °C; injection temperature, 250 °C; flow rate, 1.0 mL/min; carrier gas, N_2_ [22].

**Cytotoxic activity, NO production, NGF secretion, and cell viability assays.** The biological activity assays were performed for all compounds isolated using methods described in Supporting Information File 1.

**Computational analysis**. All conformers of **1**–**3** used in this study were found using the Macromodel (version 2019-2, Schrödinger LLC) module with “Mixed torsional/Low-mode sampling” in the MMFF force field. The searches were implemented in the gas phase with a 15 kJ/mol energy window limit and 10,000 maximum number of steps to explore all potential conformers. The Polak–Ribiere conjugate gradient (PRCG) method was utilized to minimize conformers with 10,000 iterations and a 0.001 kJ (mol Å)^−1^ convergence threshold on the root mean quare (RMS) gradient. All the conformers of **1**–**3** within 3 kJ/mol of each global minimum were subjected to geometry optimization using the Gaussian 16 package (Gaussian Inc.) in the gas phase at B3LYP/6-31G(d) level and proceeded to calculation of excitation energies, oscillator strength, and rotatory strength at B3LYP/SVP and B3LYP/6-31G(d) levels in the polarizable continuum model (PCM, methanol). The GIAO magnetic shielding tensors were calculated at the mPW1PW91/6–31G(d,p) level in the PCM (MeOH) and averaged based on the Boltzmann populations of each conformer in the associated Gibbs free energy (Supporting Information File 1, Figure S45). The ECD spectra were Boltzmann-weighted and generated using SpecDis software (Version 1.71) [23] with a σ/γ value of 0.30 eV. The chemical shift values were calculated via an equation below [24] (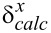: calculated ^13^C NMR chemical shift for nucleus *x*, σ^0^: shielding tensor for the carbon nuclei in tetramethylsilane, σ*^x^*: shielding tensor for the carbon nucleus *x*)


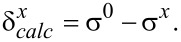


The calculated ^13^C NMR chemical shifit of **2** were averaged as described above and used for calculations of DP4+ probability using an Excel sheet [25].

## Supporting Information

File 1 HRESIMS and 1D and 2D NMR data of compounds **1**–**5**, experimental ECD data of **1**–**3**, comparison with standard samples and monosaccharides of **1**–**5**, coordinates of the conformers, cytotoxic activity, NO production, NGF secretion, and cell viability assays.
