# Supplementary material for: Terpenoids from Glechoma hederacea var. longituba and their biological activities
Source: Beilstein J Org Chem. 2022 May 17;18:555–66. doi: 10.3762/bjoc.18.58 (PMC9127242; doi:10.3762/bjoc.18.58)
Supplement: File 1 — HRESIMS and 1D and 2D NMR data of compounds 1–5, experimental ECD data of 1–3, comparison with standard samples and monosaccharides of 1–5, coordinates of the conformers, cytotoxic activity, NO production, NGF secretion, and cell viability assays. [file Beilstein_J_Org_Chem-18-555-s001.pdf]

BEILSTEIN JOURNAL OF ORGANIC CHEMISTRY

Supporting Information

for

Terpenoids from *Glechoma hederacea* var. *longituba* and their biological activities

Dong Hyun Kim, Song Lim Ham, Zahra Khan, Sun Yeou Kim, Sang Un Choi, Chung Sub Kim and Kang Ro Lee

Beilstein J. Org. Chem. **2022,** *18,* 555–566. doi:10.3762/bjoc.18.58

HRESIMS and 1D and 2D NMR data of compounds 1–5, experimental ECD data of 1–3, comparison with standard samples and monosaccharides of 1–5, coordinates of the conformers, cytotoxic activity, NO production, NGF secretion, and cell viability assays

License and Terms: This is a supporting information file under the terms of the Creative Commons Attribution License (https://creativecommons.org/licenses/by/4.0). Please note that the reuse, redistribution and reproduction in particular requires that the author(s) and source are credited and that individual graphics may be subject to special legal provisions.
The license is subject to the *Beilstein Journal of Organic Chemistry* terms and conditions: (https://www.beilstein-journals.org/bjoc/terms)

Contents

S1. Detailed experimental procedures ········ S3

Figure S1. HRESIMS data of 1 ········ S5

Figure S2. ^{1}H NMR data of 1 in CD_3OD ········ S6

Figure S3. ^{13}C NMR data of 1 in CD_3OD ········ S7

Figure S4. ^{1}H-^{1}H COSY spectrum of 1 in CD_3OD ········ S8

Figure S5. HSQC spectrum of 1 in CD_3OD ········ S9

Figure S6. HMBC spectrum of 1 in CD_3OD ········ S10

Figure S7. NOESY spectrum of 1 in CD_3OD ········ S11

Figure S8. Comparison of standard D-glucopyranose and monosaccharide obtained by hydrolysis of 1 ··· S12

Figure S9. ECD spectrum of 1 ········ S13

Figure S10. HRESIMS data of 2 ········ S14

Figure S11. ^{1}H NMR data of 2 in CD_3OD ········ S15

Figure S12. ^{13}C NMR data of 2 in CD_3OD ········ S16

Figure S13. ^{1}H-^{1}H COSY spectrum of 2 in CD_3OD ········ S17

Figure S14. HSQC spectrum of 2 in CD_3OD ········ S18

Figure S15. HMBC spectrum of 2 in CD_3OD ········ S19

Figure S16. NOESY spectrum of 2 in CD_3OD ········ S20

Figure S17. Comparison of standard D-glucopyranose and monosaccharide obtained by hydrolysis of 2 · S21

Figure S18. ECD spectrum with UV correction of 2 ········ S22

Figure S19. HRESIMS data of 3 ········ S23

Figure S20. ^{1}H NMR data of 3 in CD_3OD ········ S24

Figure S21. ^{13}C NMR data of 3 in CD_3OD ········ S25

Figure S22. ^{1}H-^{1}H COSY spectrum of 3 in CD_3OD ········ S26

Figure S23. HSQC spectrum of 3 in CD_3OD ········ S27

Figure S24. HMBC spectrum of 3 in CD_3OD ········ S28

Figure S25. NOESY spectrum of 3 in CD_3OD ········ S29

Figure S26. Comparison of standard D-glucopyranose and monosaccharide obtained by hydrolysis of 3 · S30

Figure S27. ECD spectrum of 3 ········ S31

Figure S28. HRESIMS data of 4 ········ S32

Figure S29. ^{1}H NMR data of 4 in CD_3OD ········ S33

Figure S30. ^{13}C NMR data of 4 in CD_3OD ········ S34

Figure S31. ^{1}H-^{1}H COSY spectrum of 4 in CD_3OD ········ S35

Figure S32. HSQC spectrum of 4 in CD_3OD ········ S36

Figure S33. HMBC spectrum of 4 in CD_3OD ········ S37

Figure S34. NOESY spectrum of 4 in CD_3OD ········ S38

Figure S35. Comparison of standard D-glucopyranose and monosaccharide obtained by hydrolysis of 4 · S39

Figure S36. HRESIMS data of 5 ········· S40

Figure S37. ^{1}H NMR data of 5 in CD_3OD ········· S41

Figure S38. ^{13}C NMR data of 5 in CD_3OD ········· S42

Figure S39. ^{1}H-^{1}H COSY spectrum of 5 in CD_3OD ········· S43

Figure S40. HSQC spectrum of 5 in CD_3OD ········· S44

Figure S41. HMBC spectrum of 5 in CD_3OD ········· S45

Figure S42. NOESY spectrum of 5 in CD_3OD ········· S46

Figure S43. Comparison of standard D-glucopyranose and monosaccharide obtained by hydrolysis of 5 · S47

Figure S44. Optimized geometries of conformers of compound 1 at B3LYP/6-31G(d) ········· S48

Figure S45. Optimized geometries of conformers of compound 2 (Boltzmann populations in parenthesis) at B3LYP/6-31G(d) ········· S49

Figure S46. Optimized geometries of conformers of compound 3 at B3LYP/6-31G(d) ········· S49

Table S1. Sum of electronic and zero-point energies (ZPE), thermal energies (E), thermal enthalpies (H), thermal free energies(G) calculated at B3LYP/6-31G(d) level for individual conformers of compound 1 with the use of an PCM model of methanol. ········· S50

Table S2. Sum of electronic and zero-point energies (ZPE), thermal energies (E), thermal enthalpies (H), thermal free energies(G) calculated at B3LYP/6-31G(d) level for individual conformers of compound 2 with the use of an PCM model of methanol. ········· S50

Table S3. Sum of electronic and zero-point energies (ZPE), thermal energies (E), thermal enthalpies (H), thermal free energies(G) calculated at B3LYP/6-31G(d) level for individual conformers of compound 3 with the use of an PCM model of methanol. ········· S50

Table S4. Experimental (Exp.) and calculated (Cal.) ^{13}C chemical shift values of 2 and its possible isomers 2a and 2b used for DP4+ analysis. ········· S51

Table S5. Coordinates of the conformers 1-1 – 1-8. ········· S52

Table S6. Coordinates of the conformers 2-R1, 2-S1– 2-S3. ········· S68

Table S7. Coordinates of the conformers 3-1 – 3-5. ········· S76

S1. Detailed experimental procedures

Evaluation of NO Production and Cell viability in LPS-stressed BV-2 cells.

The inhibitory effect of the compounds on NO production was tested using BV-2 cells [1,2]. BV-2 cells were seeded on a 96-well plate (4×10^4 cells/well), which treated with or without different concentrations of the compounds. LPS (100 ng/mL) was added to BV-2 cells followed by incubation for 24 h. The concentration of nitrite (NO_2) in the culture medium was evaluated using Gries reagent (0.1% *N*-1-naphthylethylenediamine dihydrochloride and 1% sulfanilamide in 5% phosphoric acid). 50 μL of supernatant was mixed with an equal volume of Gries reagent. Absorbance was measured after 10 min using a microplate reader (Emax, Molecular Devices, Sunnyvale, CA, USA) at 570 nm wavelength. L-NMMA, well-known nitric oxide synthase (NOS) inhibitor [3], was used as a positive control. Graded sodium nitrite solution was used as a standard to calculate nitrite concentrations. Cell viability was assessed by observing the ability of viable cells to reduce the yellow-colored MTT to a purple-colored formazan, using an MTT assay, as described previously [1].

NGF and Cell viability Assays.

Analogous as described in [4]. The C6 glioma cells (Korean Cell Line Bank, Seoul, Republic of Korea) were used to assess the release of NGF into the culture medium. The test cells were seeded onto 24-well plates at a density of 1×10^5 cells/well. After 24 h, cells were treated with serum-free Dulbecco's modified Eagle's medium and incubated with the designated concentrations of compounds for an additional 24 h. The medium supernatant was collected from the culture plates, and NGF levels were evaluated using an ELISA development kit. Cell viability was also assessed with a 3- [4,5-dimethylthiazol-2-yl]-2,5-diphenyltetrazolium bromide (MTT) assay in which the results were expressed as a percentage of the control group (untreated cells).

Cytotoxicity assay against four human cancer cell lines using SRB assay

A sulforhodamine B bioassay (SRB) was used to determine the cytotoxicity of each compound on four cultured human cancer cell lines [5]. The assays were performed at the Korea Research Institute of Chemical Technology. The cell lines used were A549 (non small cell lung adenocarcinoma), SK-OV-3 (ovarian cancer

cells), SK-MEL-2 (skin melanoma), and HCT15 (colon cancer cells). Doxorubicin (Sigma Chemical Co., C98 %) was used as a positive control [5].

Reference

1. Blasi, E.; Barluzzi, R.; Bocchini, V.; Mazzolla, R.; Bistoni, F. *J. Neuroimmunol.* **1990,** *27*, 229-237. doi: 10.1016/0165-5728(90)90073-V

2. Choi, Y.; Lee, M.; Lim, S.; Sung, S.; Kim, Y. *Br. J. Pharmacol.* **2009,** *156*, 933-940. doi: 10.1111/j.1476-5381.2009.00022.x

3. Reif, D. W.; McCreedy, S. A. *Arch. Biochem. Biophys* **1995,** *320*, 170-176. doi: 10.1006/abbi.1995.1356

4. Kim, C. S.; Oh, J.; Subedi, L.; Kim, S. Y.; Choi, S. U.; Lee, K. R. *J. Nat. Prod.* **2018,** *81*, 1795-1802. doi: 10.1021/acs.jnatprod.8b002455. Skehan, P.; Storeng, R.; Scudiero, D.; Monks, A.; McMahon, J.; Vistica, D.; Warren, J. T.; Bokesch, H.; Kenney, S.; Boyd, M. R. *J. Natl Cancer Inst.* **1990,** *82*, 1107-1112. doi: 10.1093/jnci/82.13.1107

Figure S1. HRESIMS data of **1**

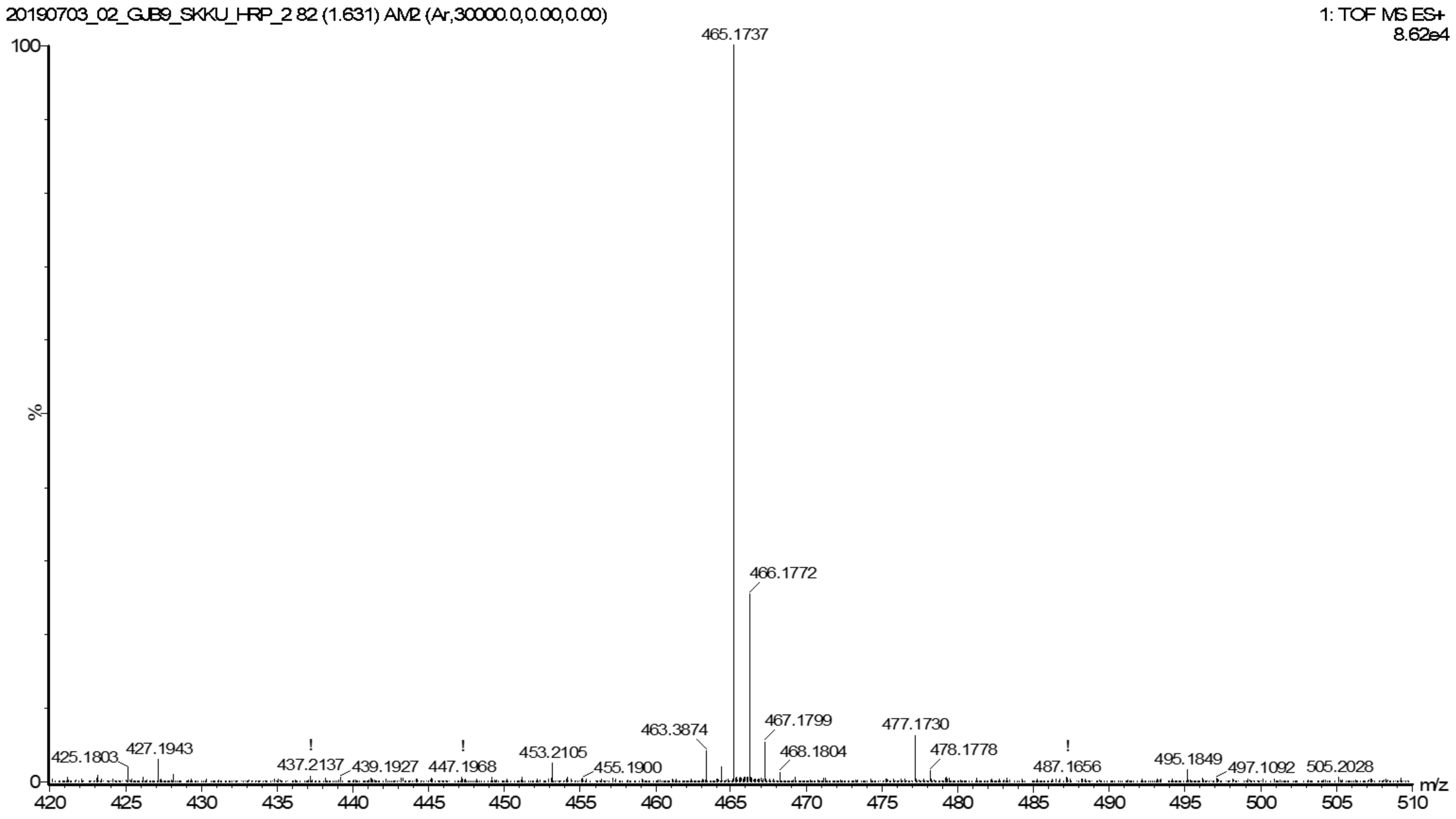

Figure S2. ^{1}H NMR data of **1** in CD_3OD

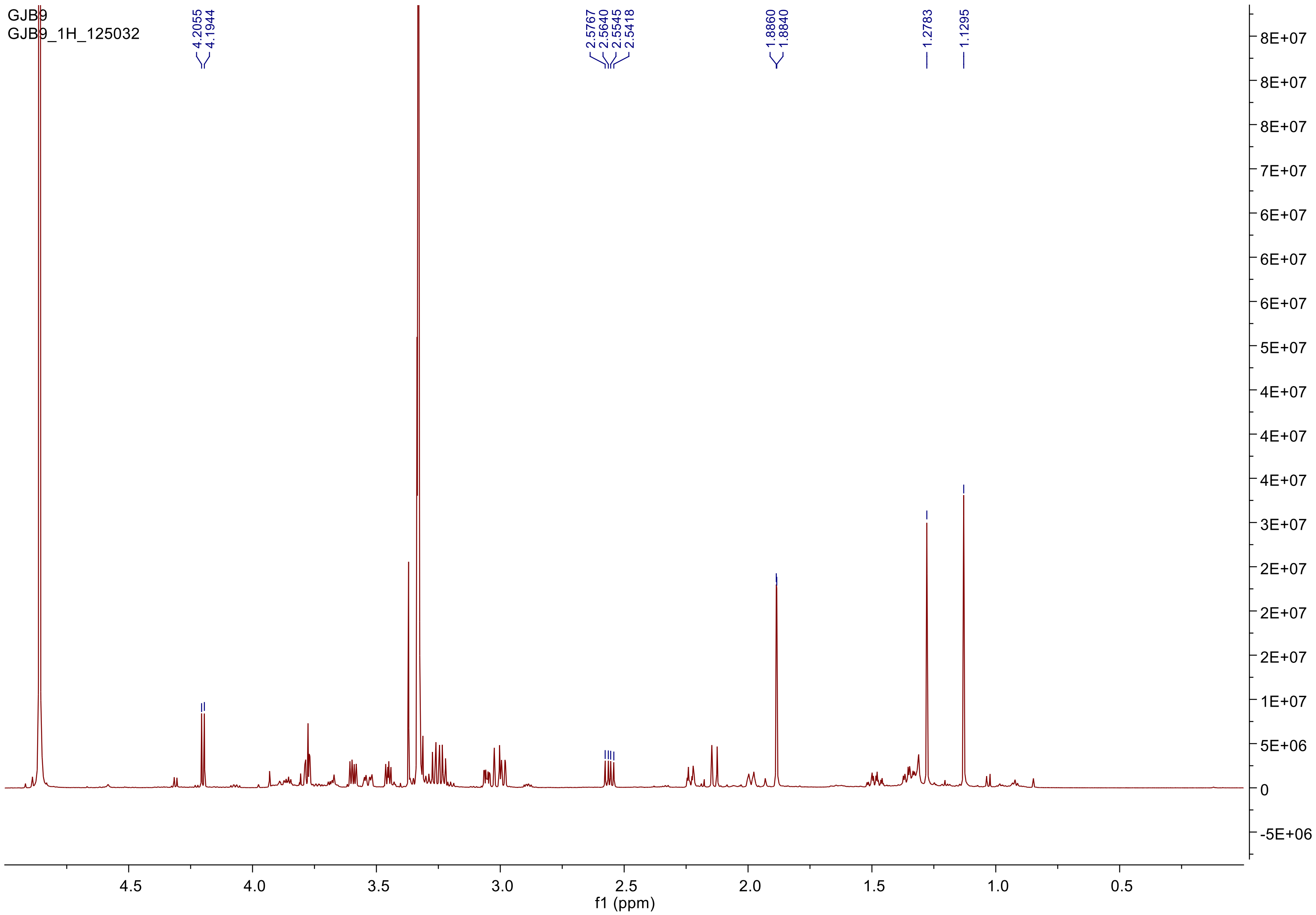

Figure S3. ^{13}C NMR data of **1** in CD_3OD

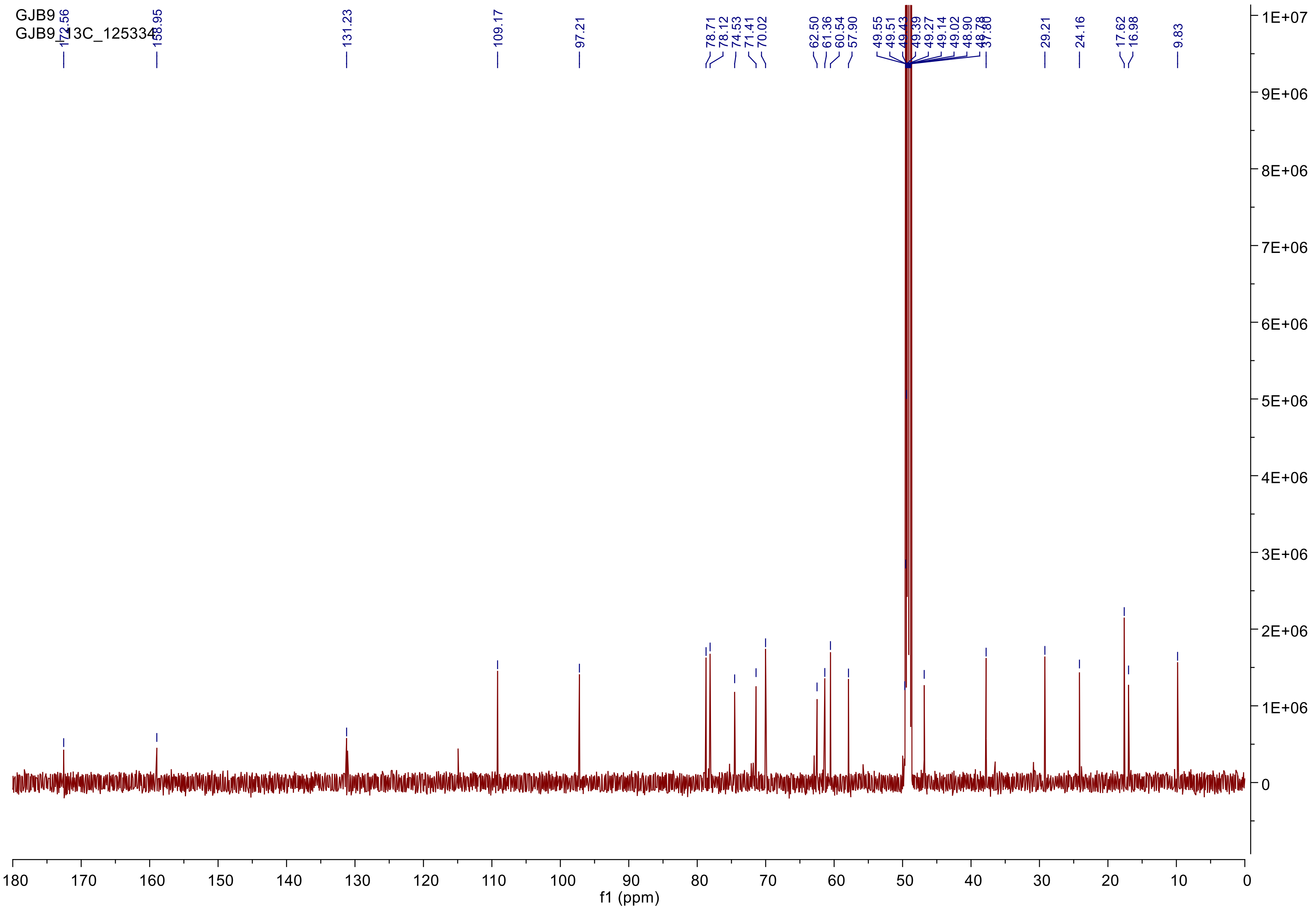

Figure S4. ^1H-^1H COSY spectrum of **1** in CD_3OD

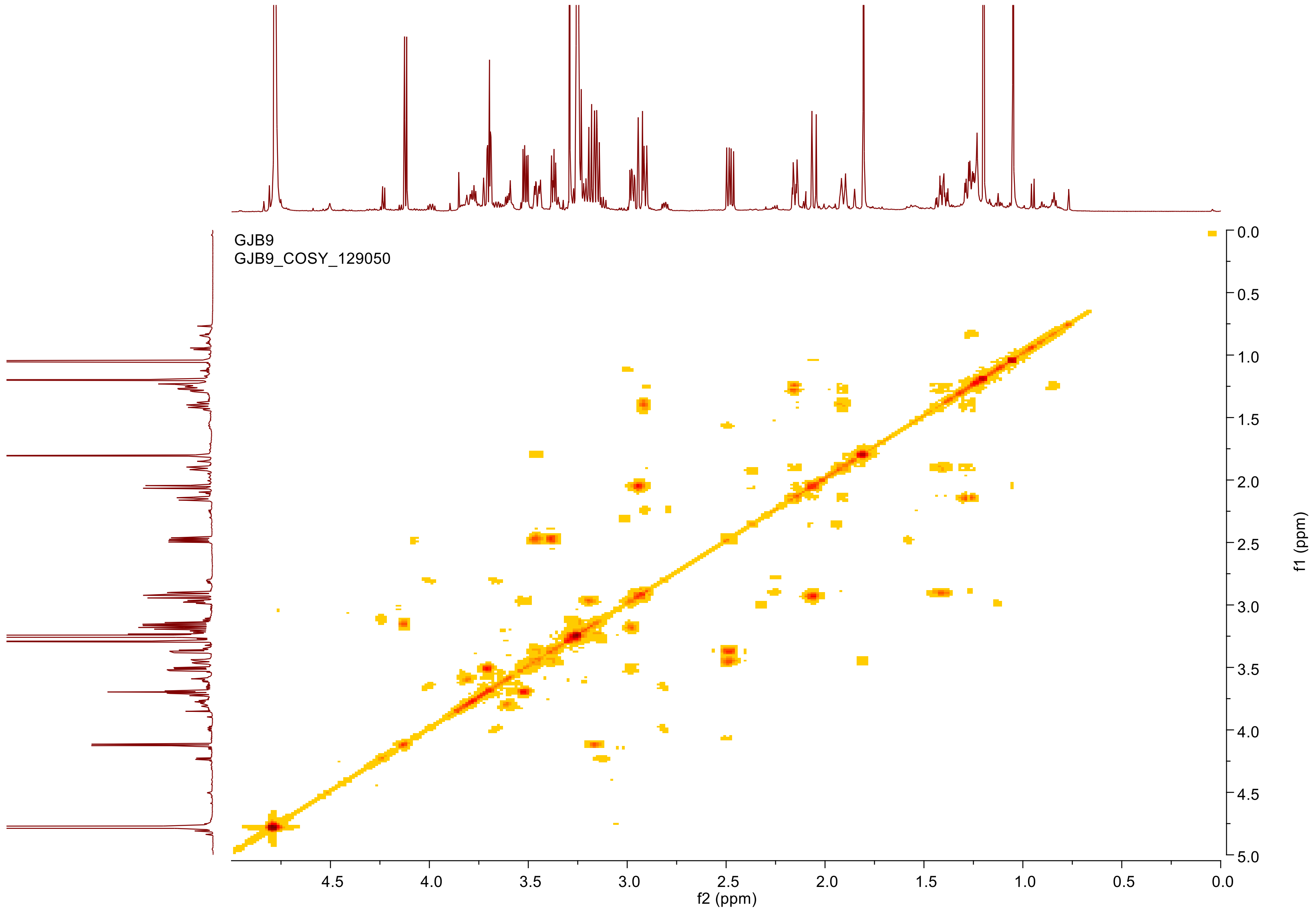

Figure S5. HSQC spectrum of **1** in CD_3OD

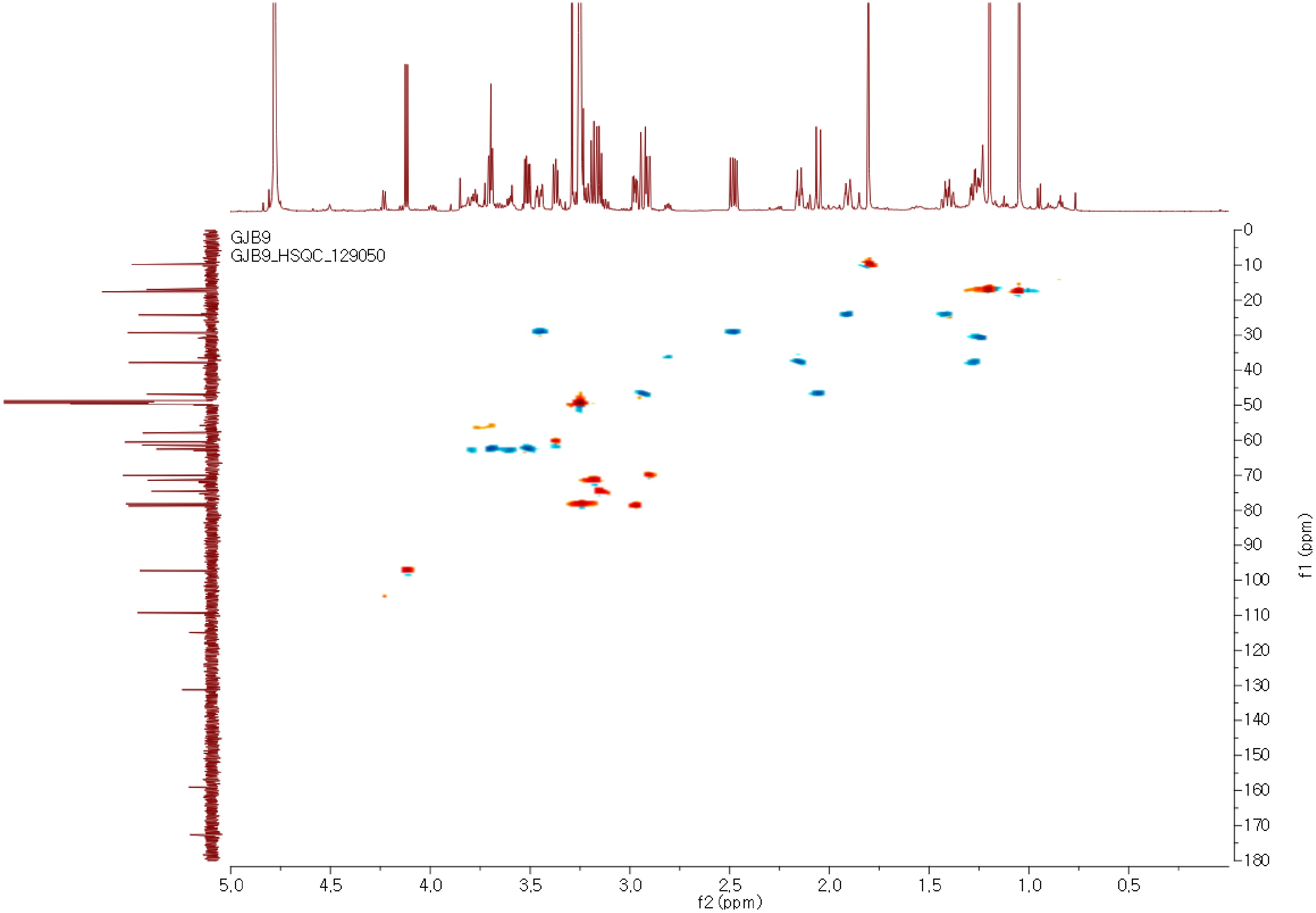

Figure S6. HMBC spectrum of **1** in CD_3OD

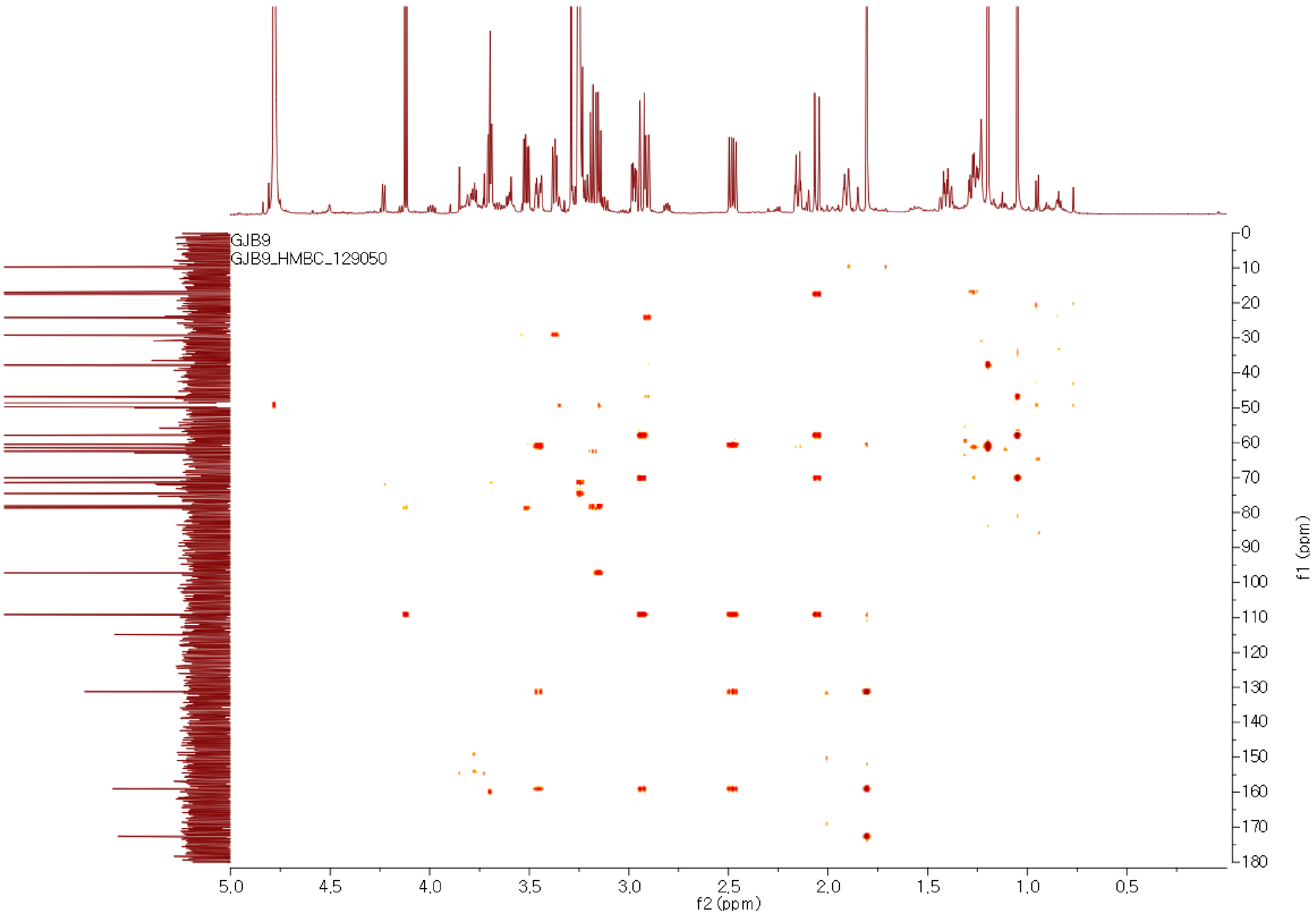

Figure S7. NOESY spectrum of **1** in CD_3OD

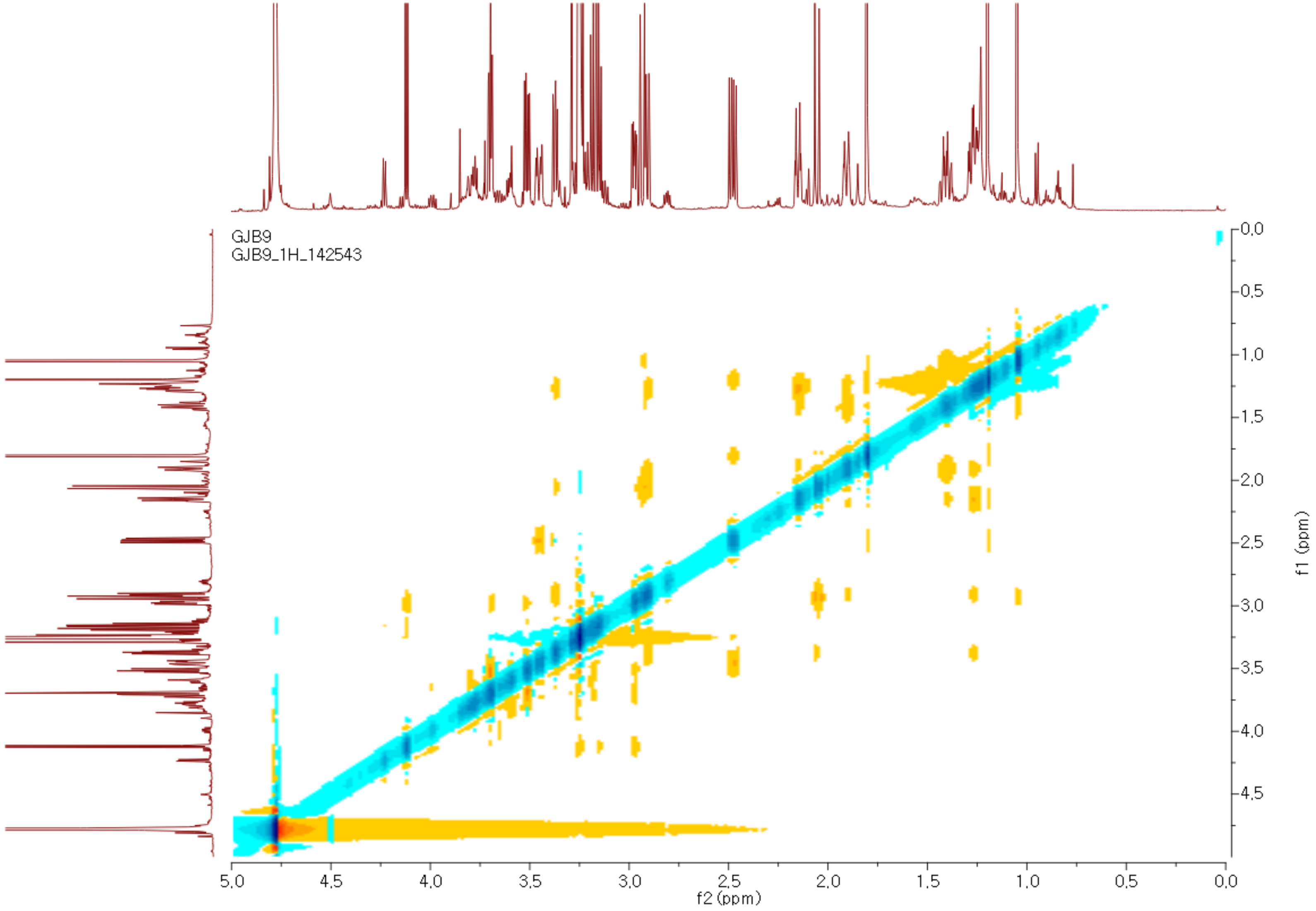

Figure S8. Comparison of standard D-glucopyranose and monosaccharide obtained by hydrolysis of **1**

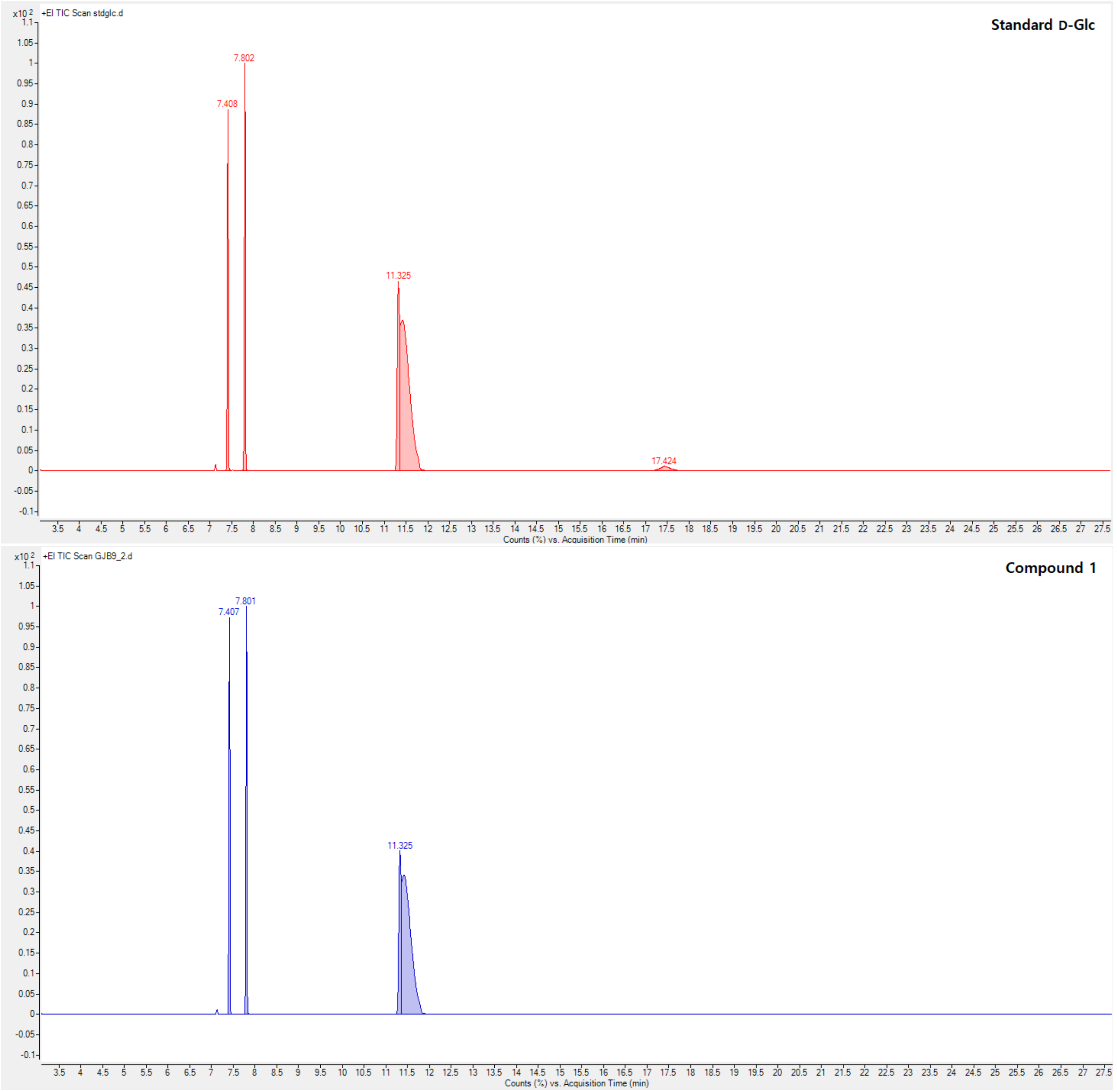

Figure S9. ECD spectrum of **1**

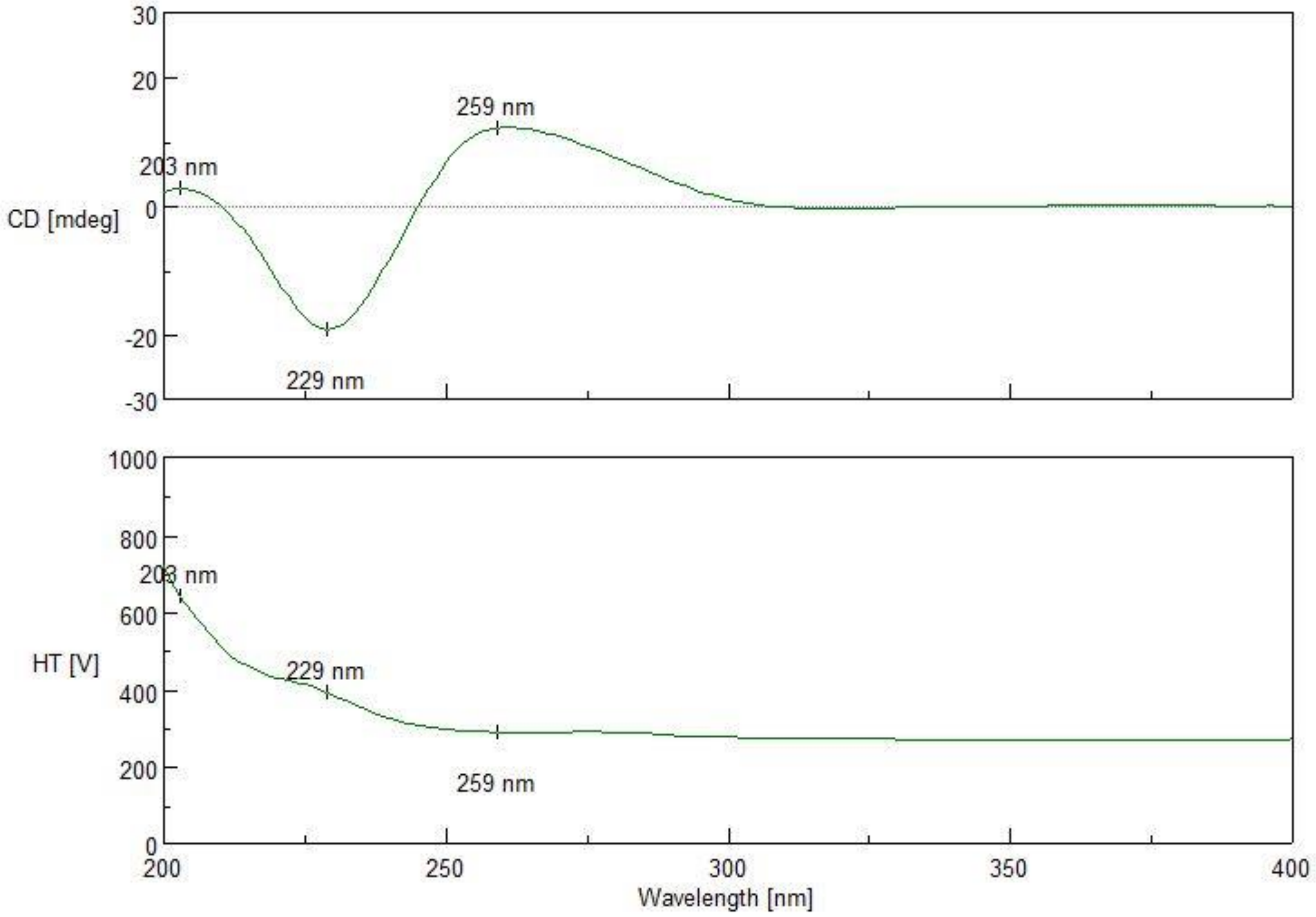

Figure S10. HRESIMS data of **2**

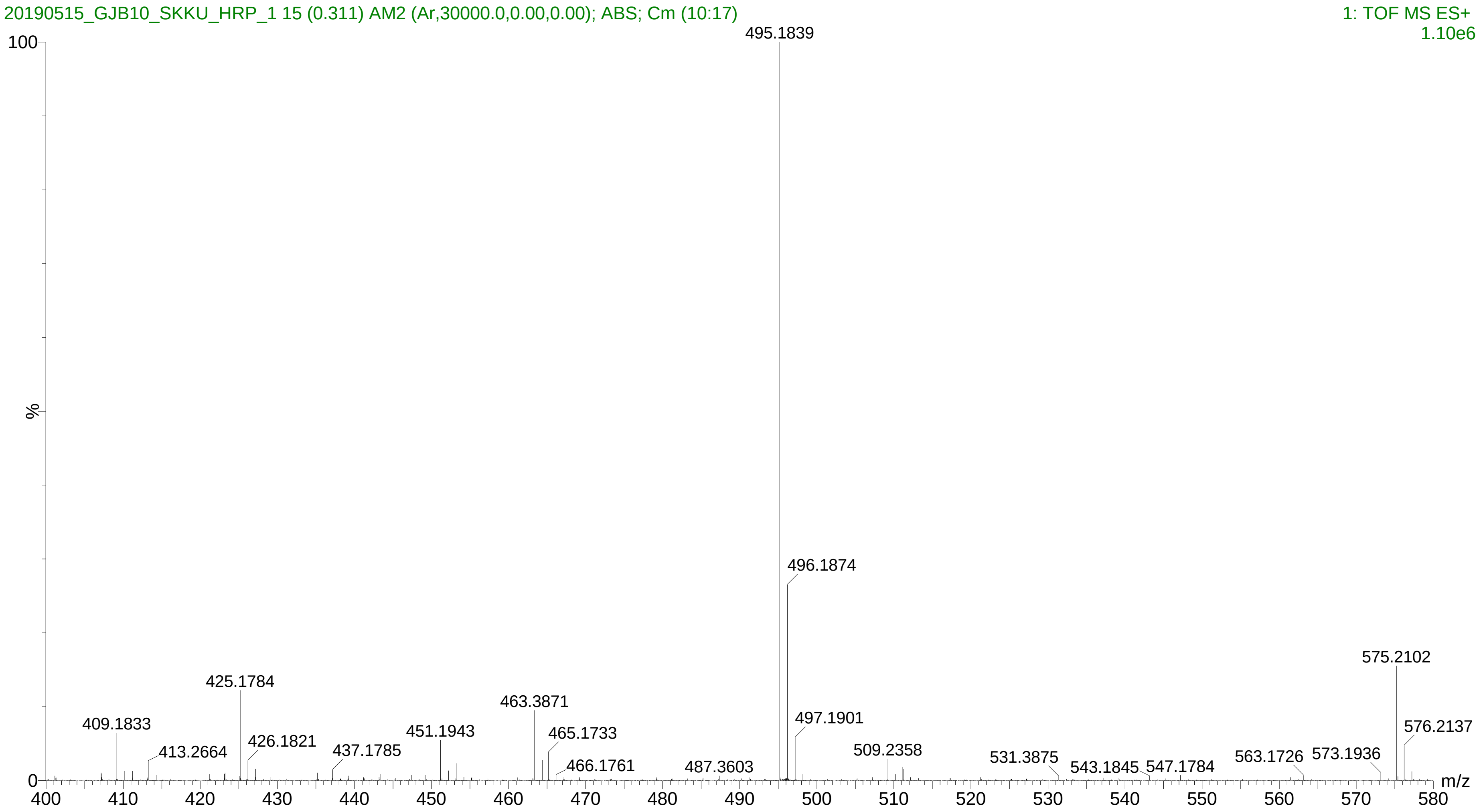

Figure S11. ^{1}H NMR data of **2** in CD_3OD

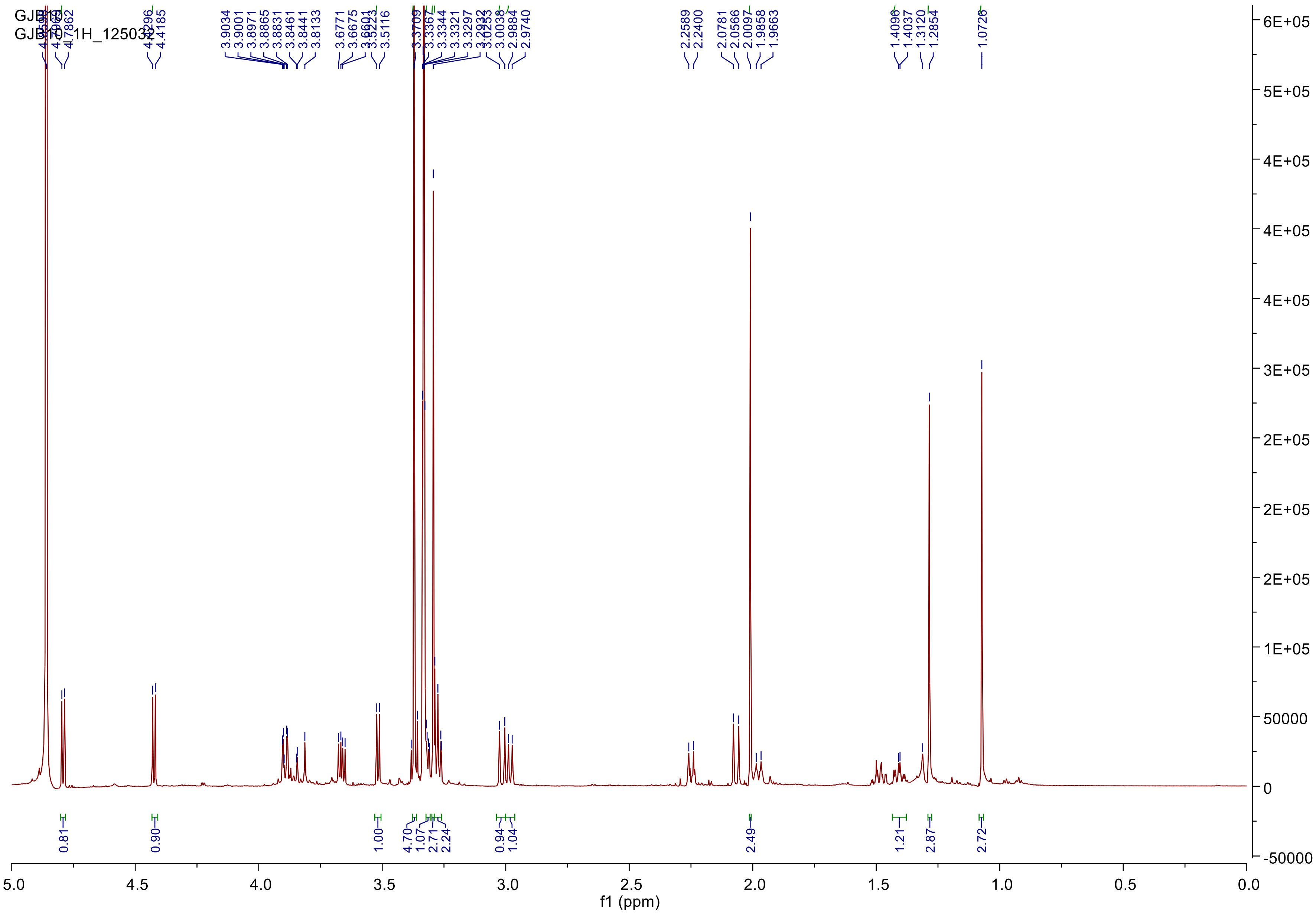

Figure S12. ^{13}C NMR data of **2** in CD_3OD

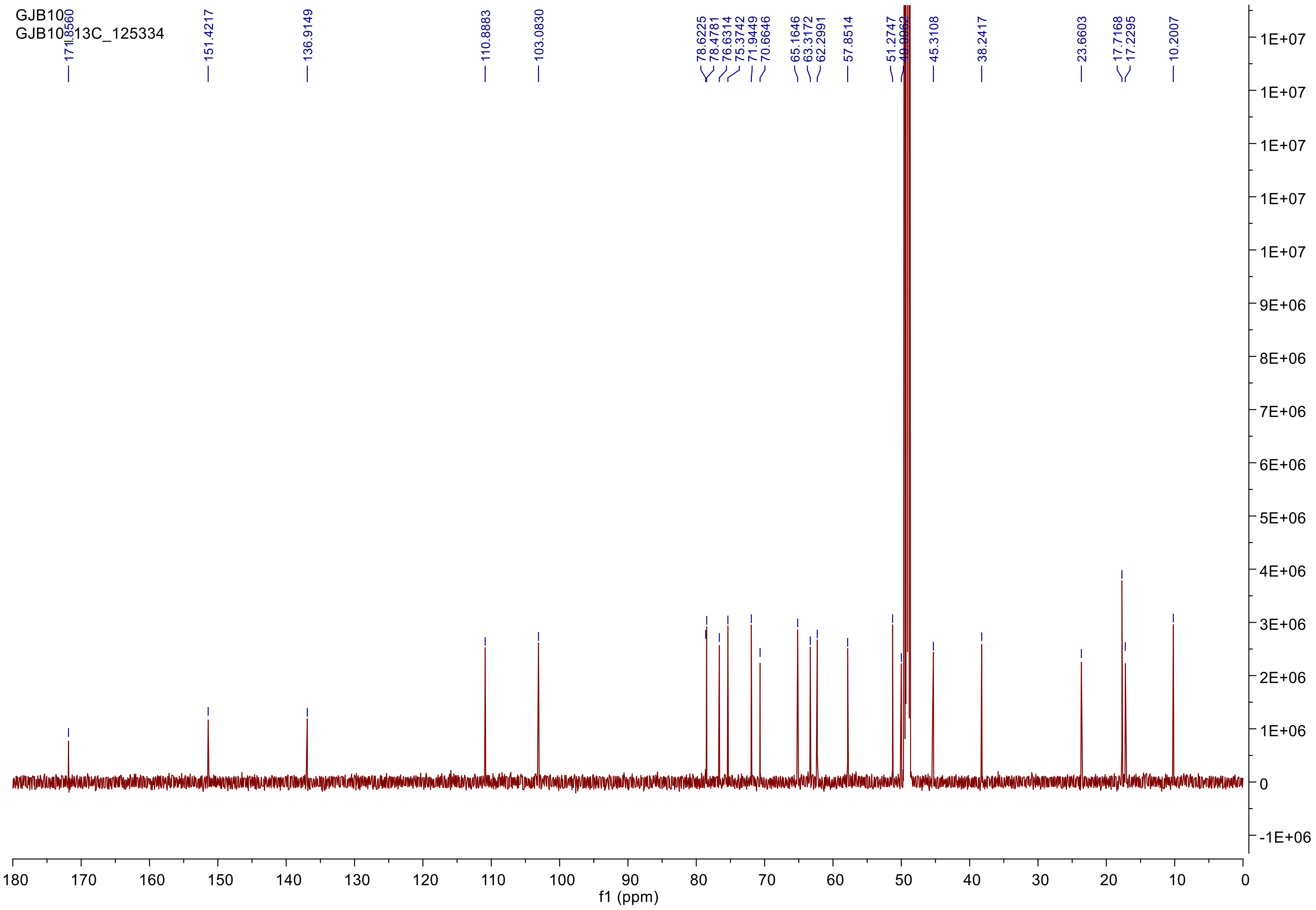

Figure S13. ^{1}H-^{1}H COSY spectrum of **2** in CD_3OD

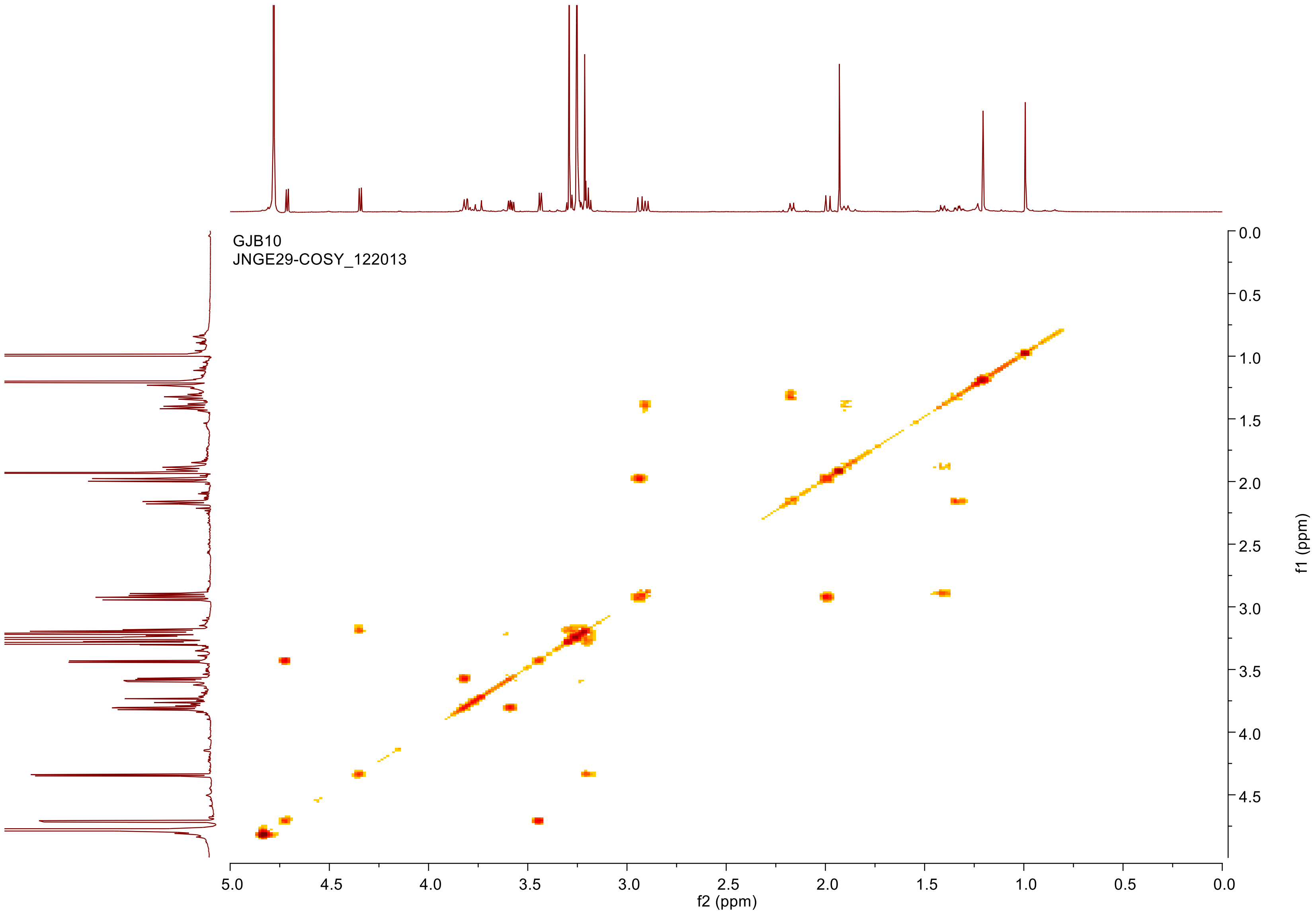

Figure S14. HSQC spectrum of **2** in CD_3OD

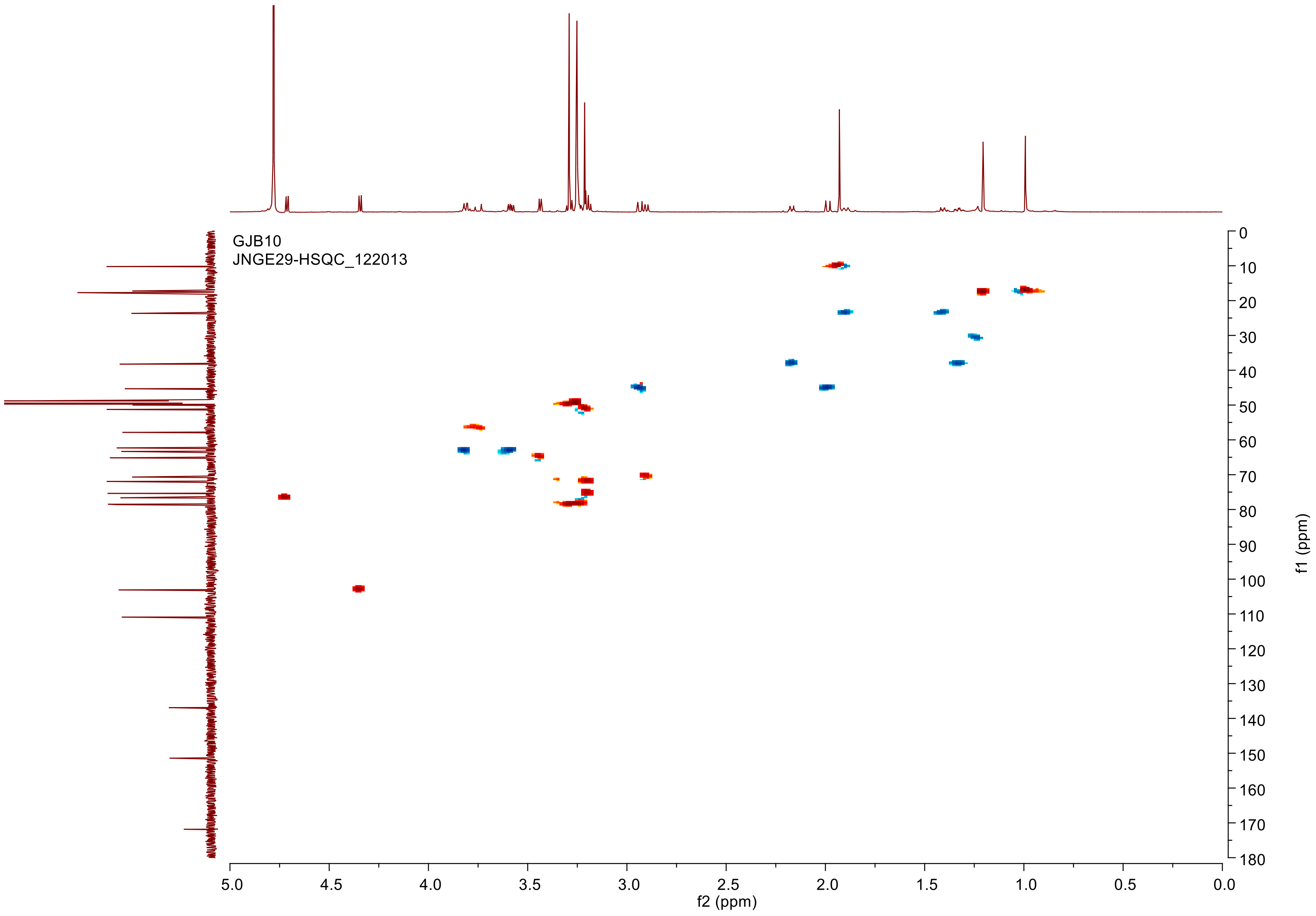

Figure S15. HMBC spectrum of **2** in CD_3OD

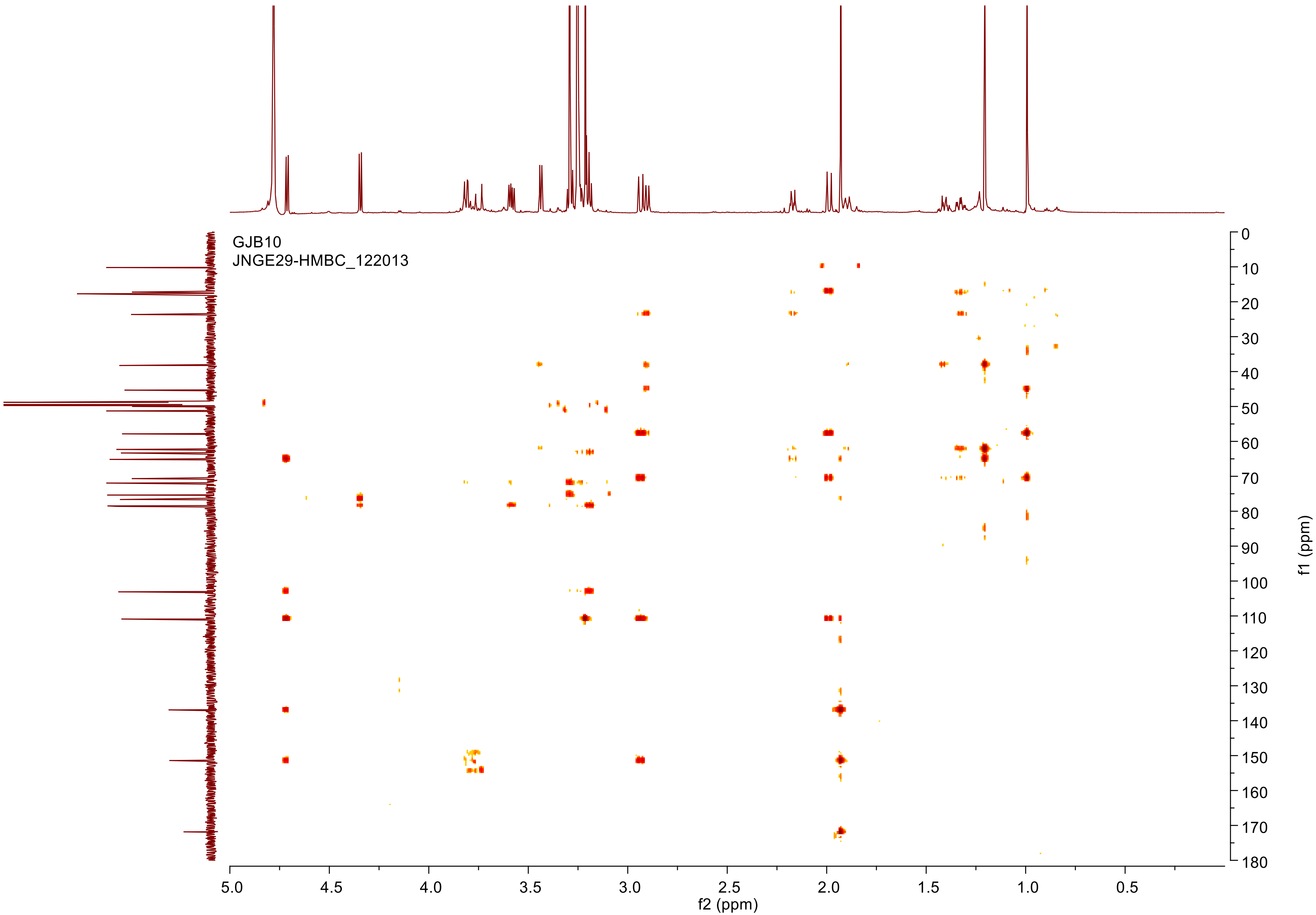

Figure S16. NOESY spectrum of **2** in CD_3OD

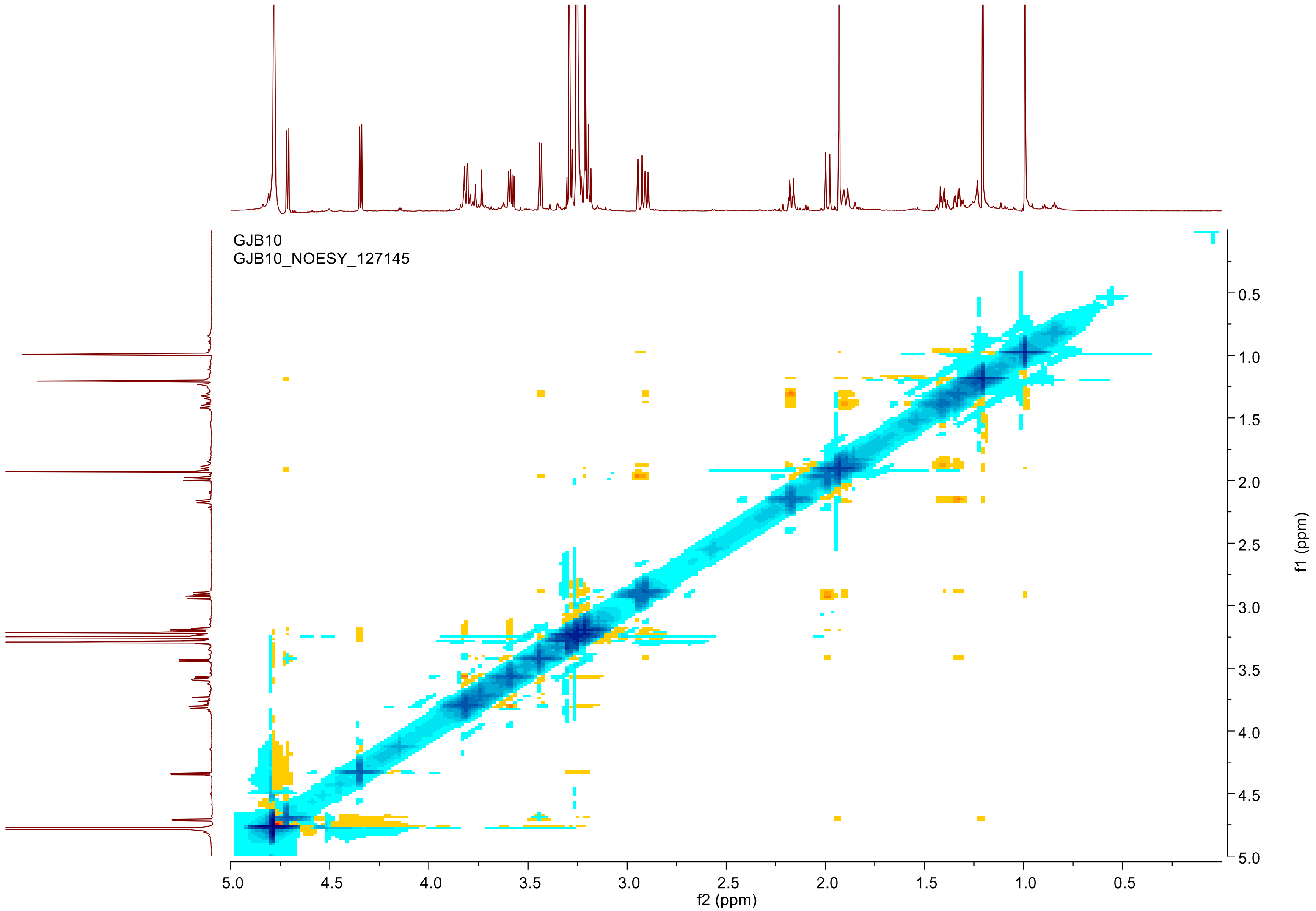

Figure S17. Comparison of standard D-glucopyranose and monosaccharide obtained by hydrolysis of **2**

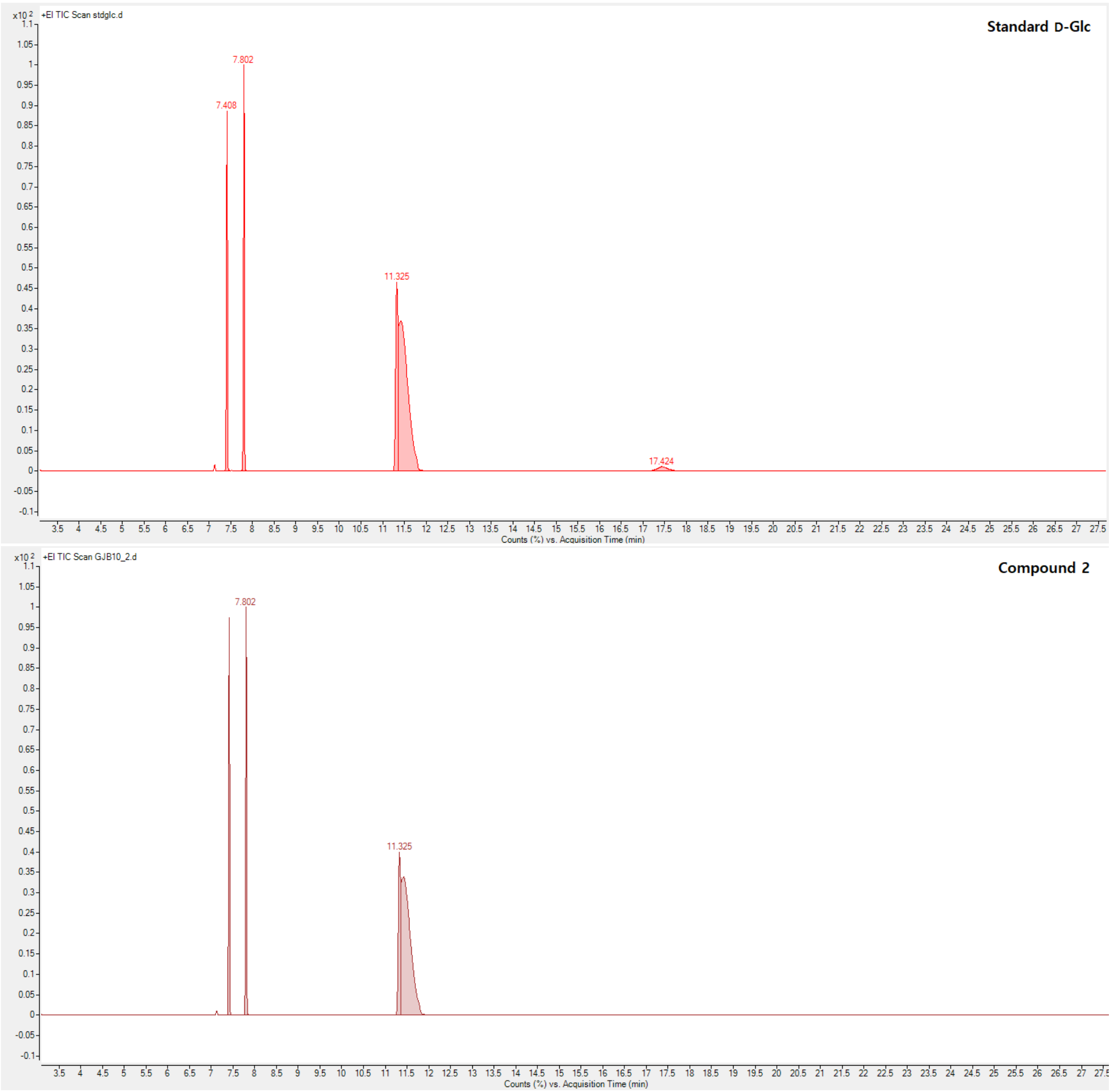

Figure S18. ECD spectrum with UV correction of **2**

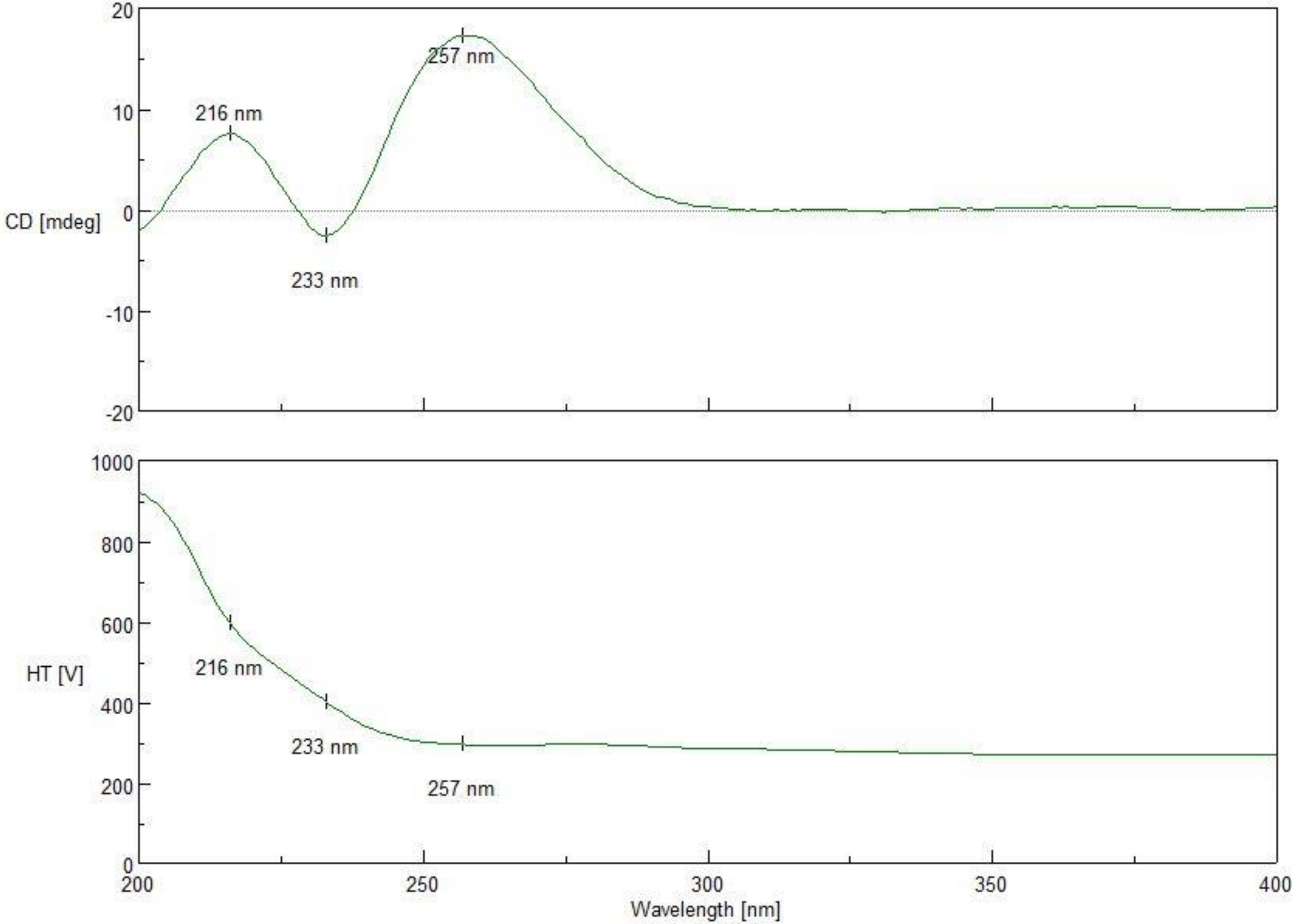

Figure S19. HRESIMS data of **3**

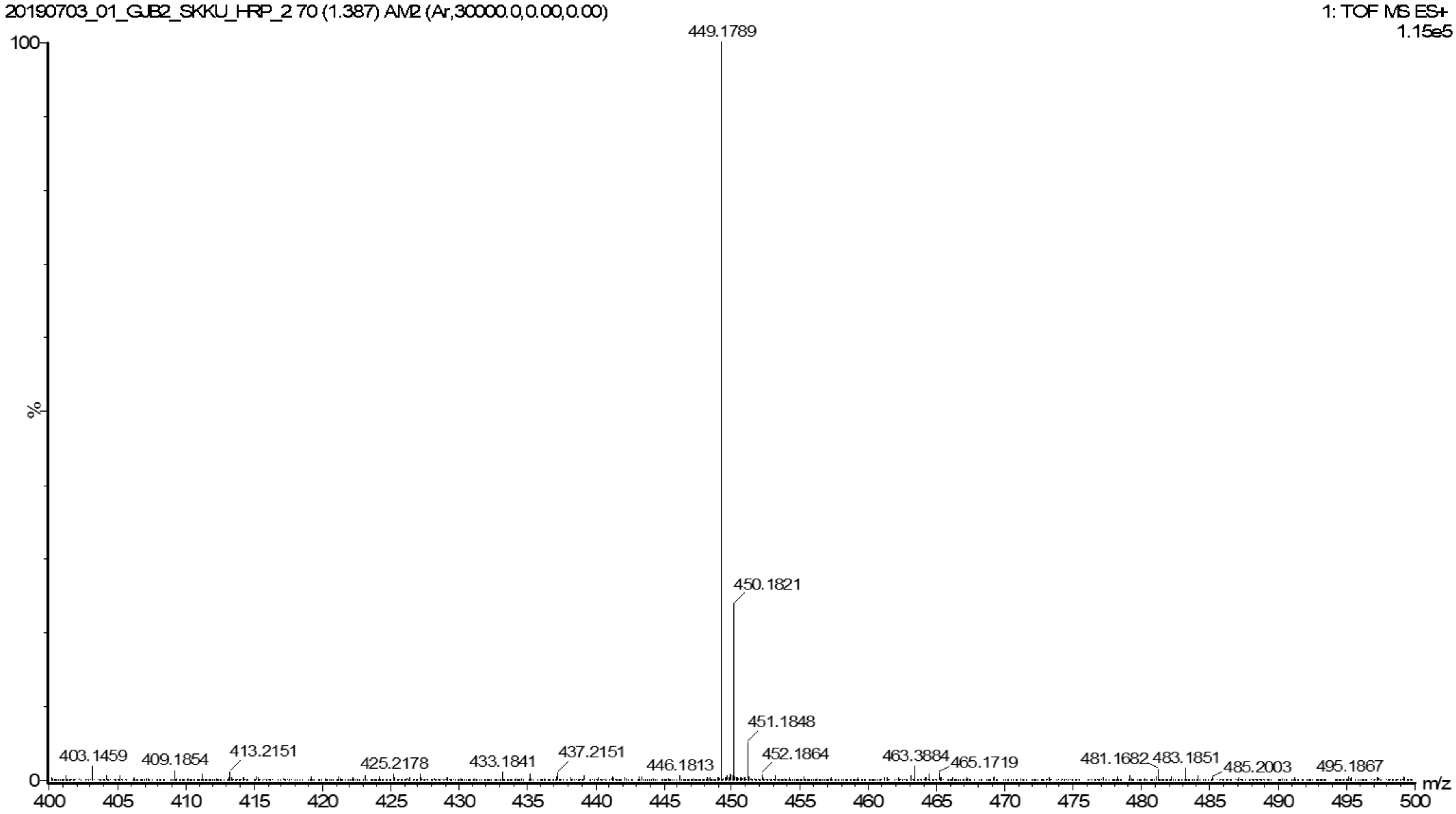

Figure S20. ^{1}H NMR data of **3** in CD_3OD

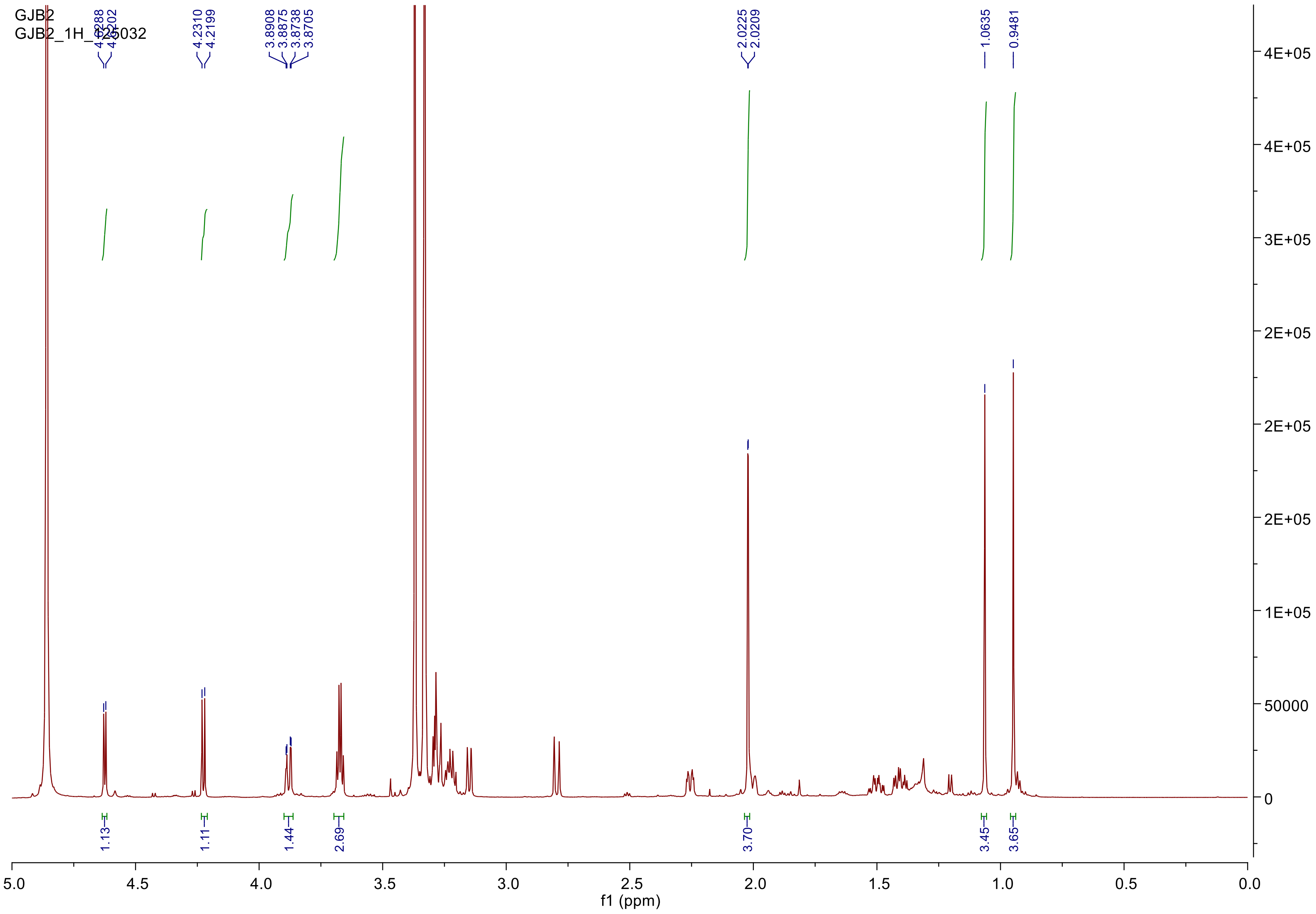

Figure S21. ^{13}C NMR data of **3** in CD_3OD

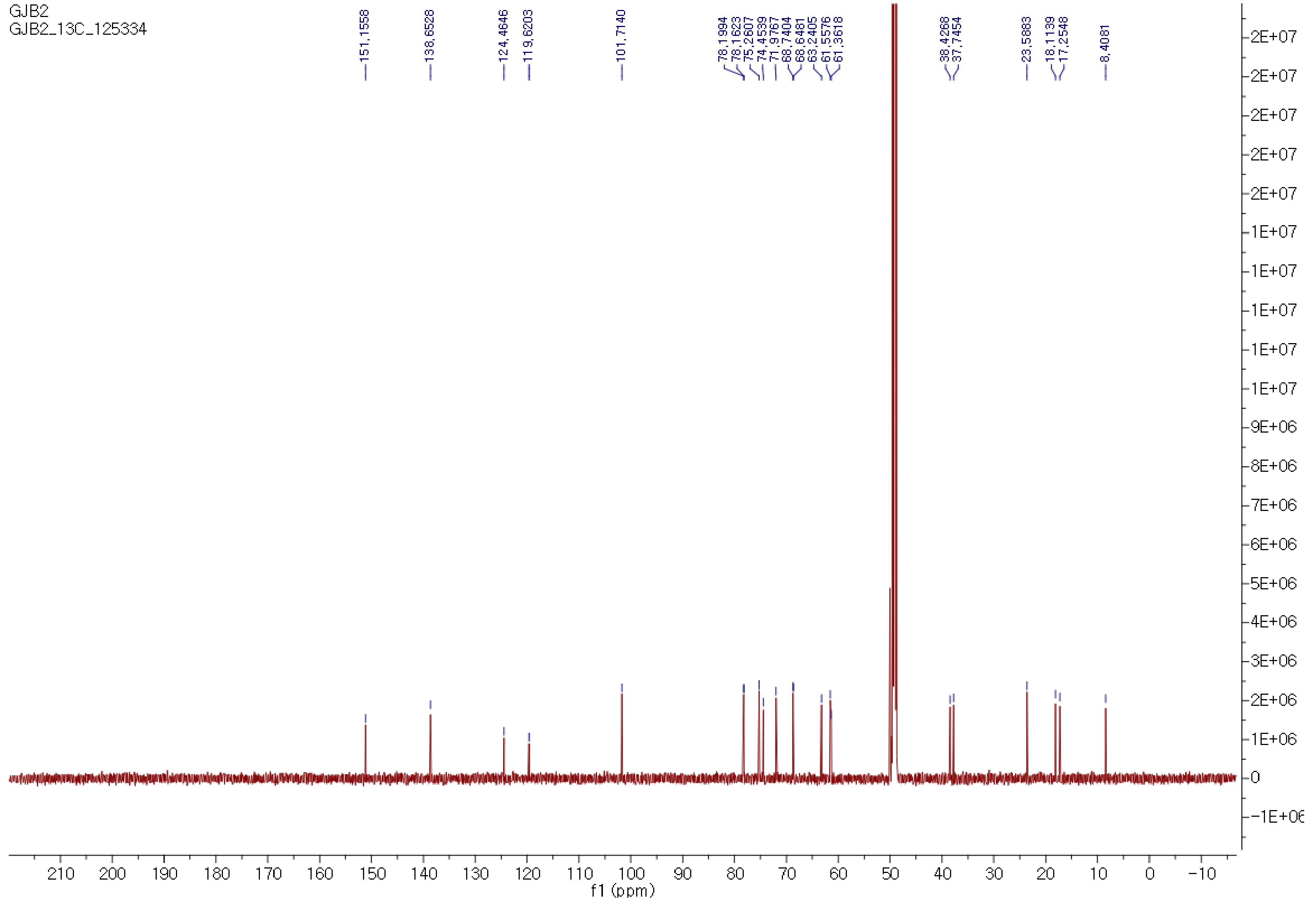

Figure S22. ^{1}H-^{1}H COSY spectrum of **3** in CD_3OD

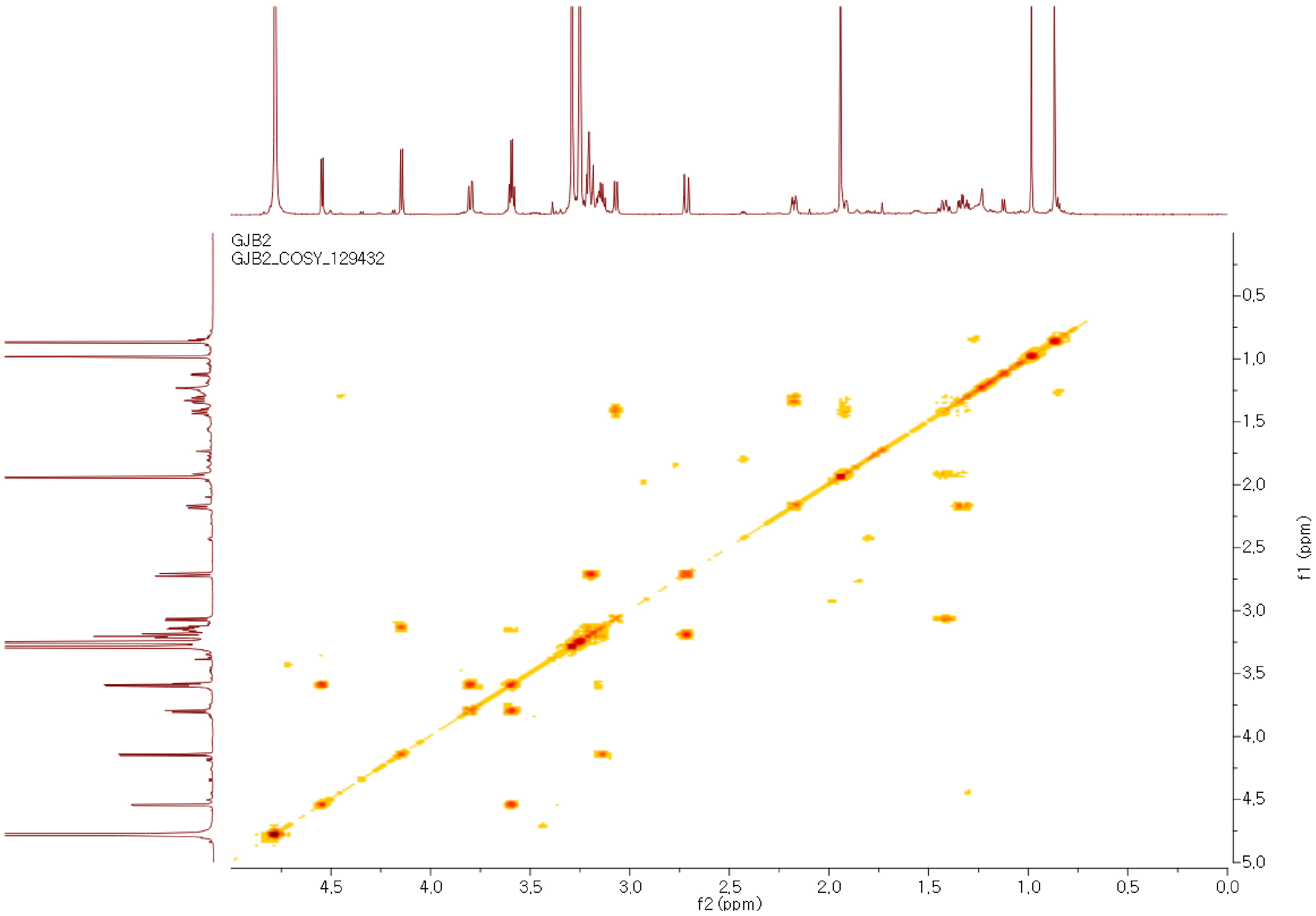

Figure S23. HSQC spectrum of **3** in CD_3OD

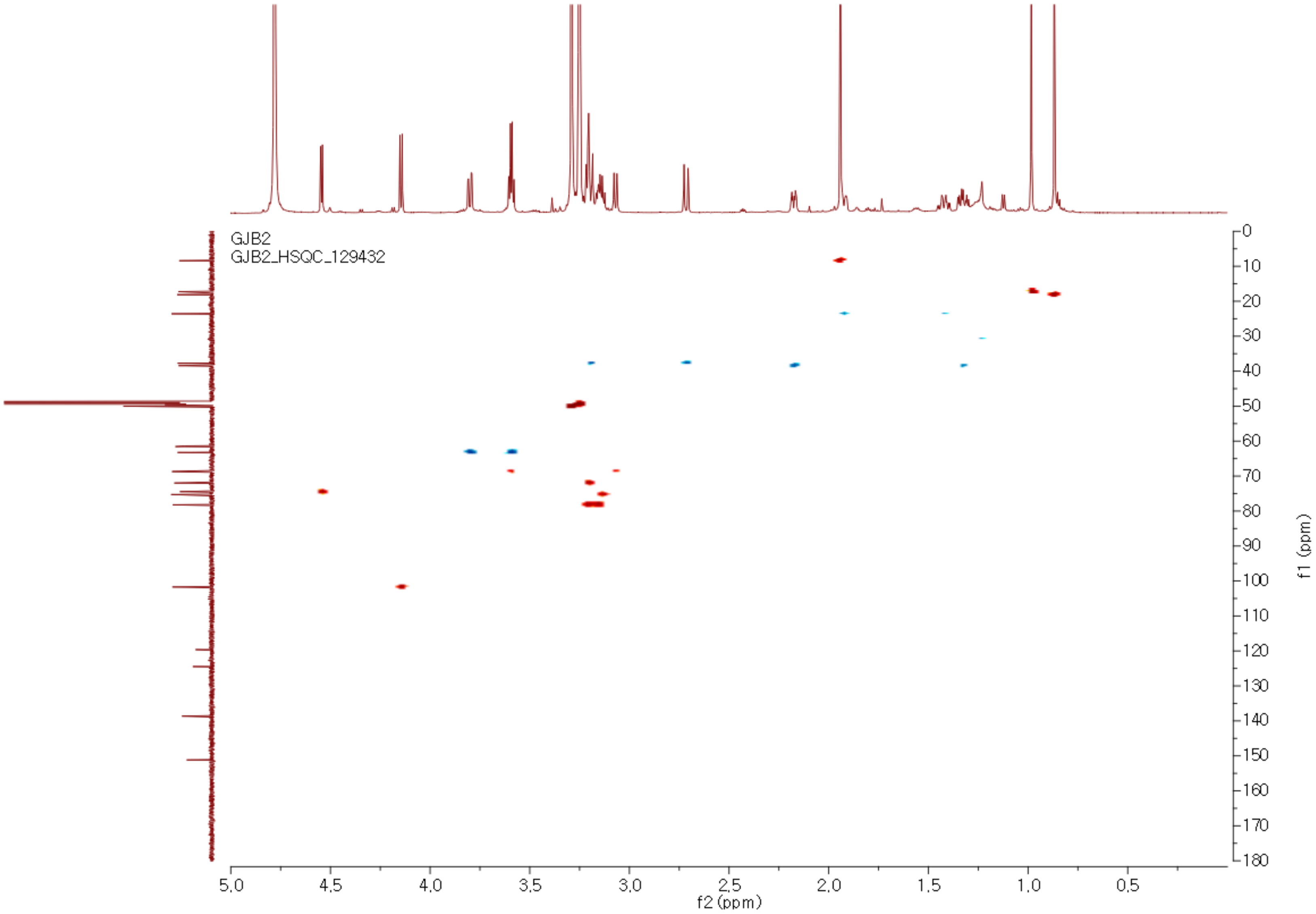

Figure S24. HMBC spectrum of **3** in CD_3OD

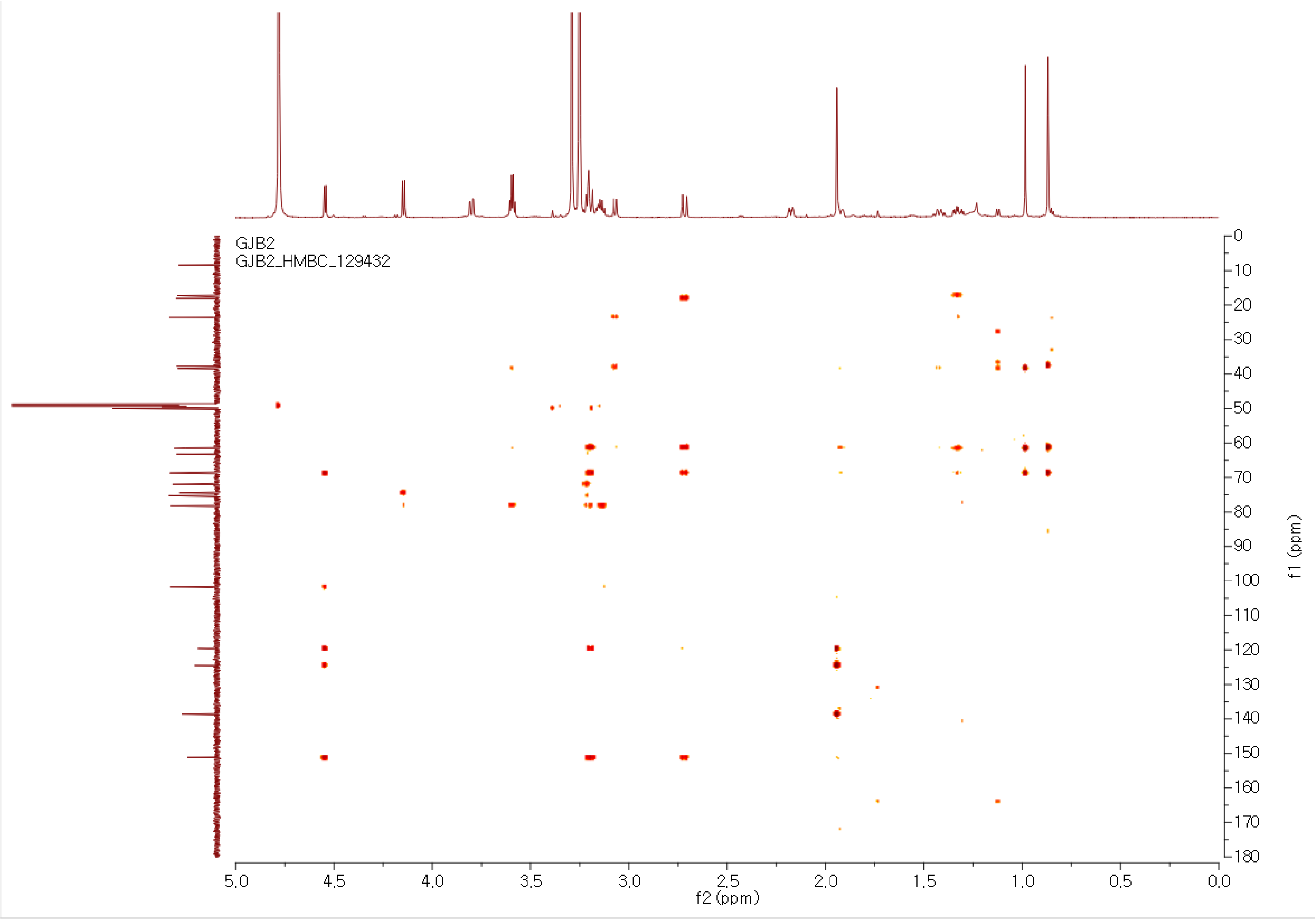

Figure S25. NOESY spectrum of **3** in CD_3OD

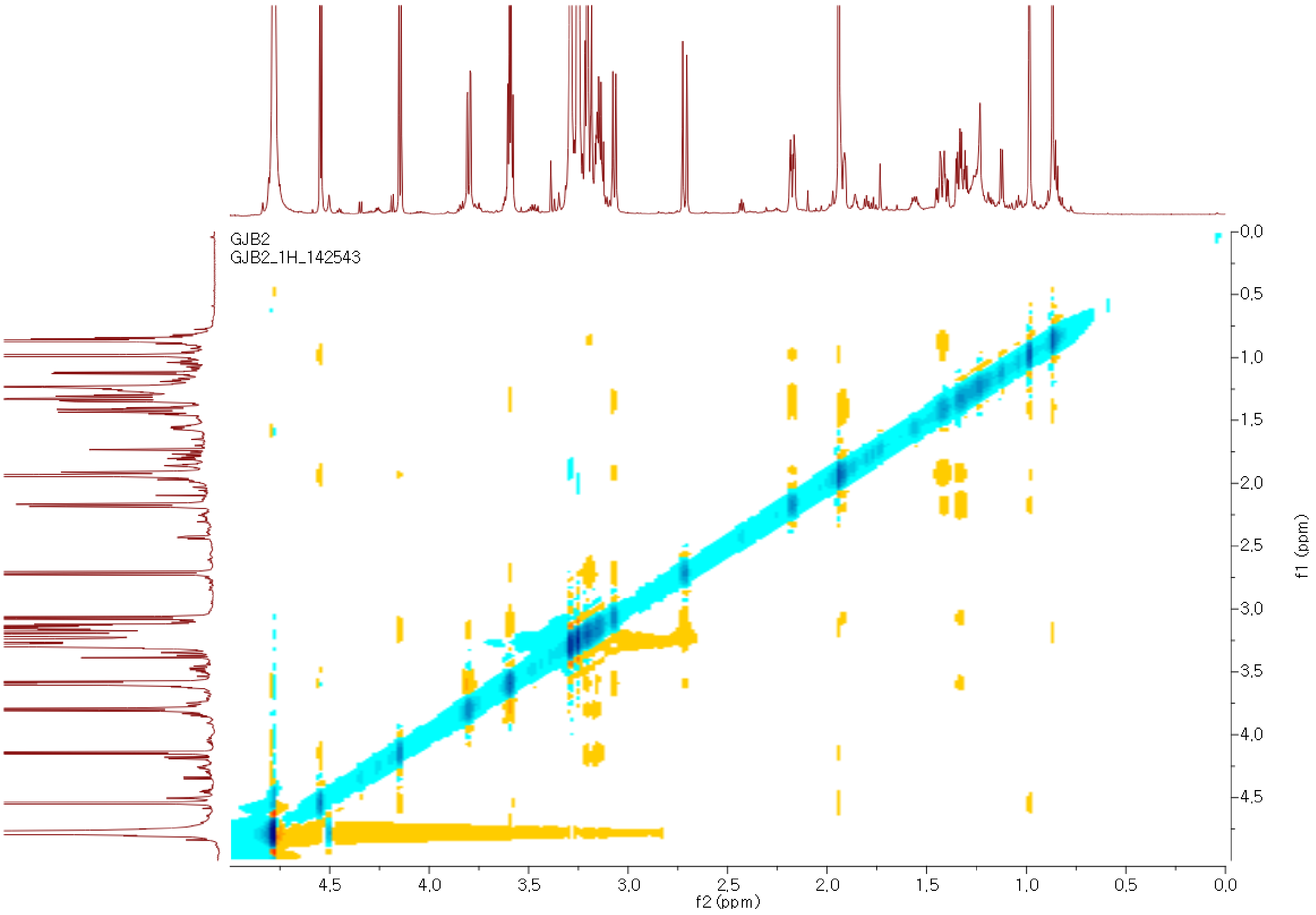

Figure S26. Comparison of standard D-glucopyranose and monosaccharide obtained by hydrolysis of **3**

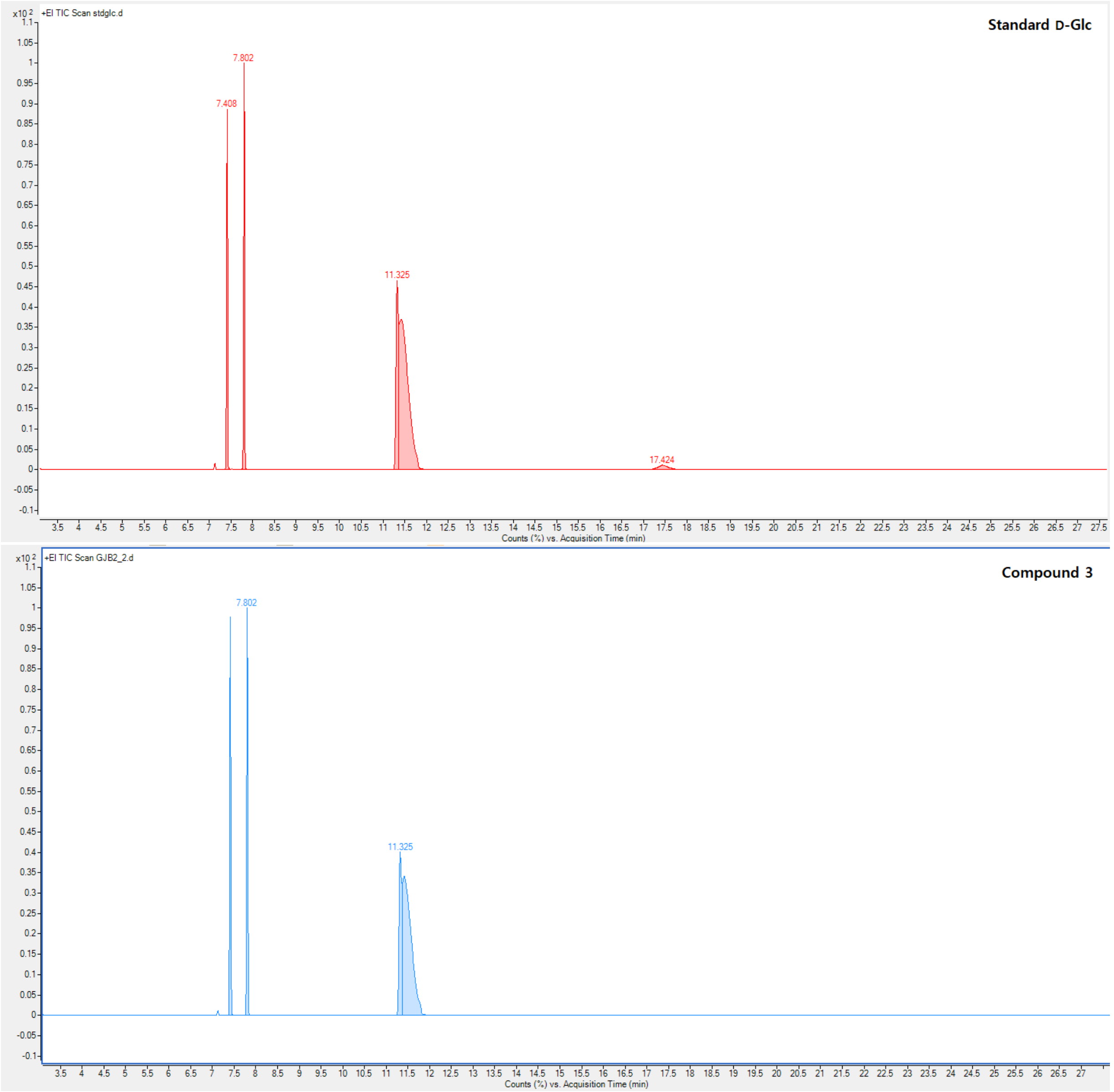

Figure S27. ECD spectrum of **3**

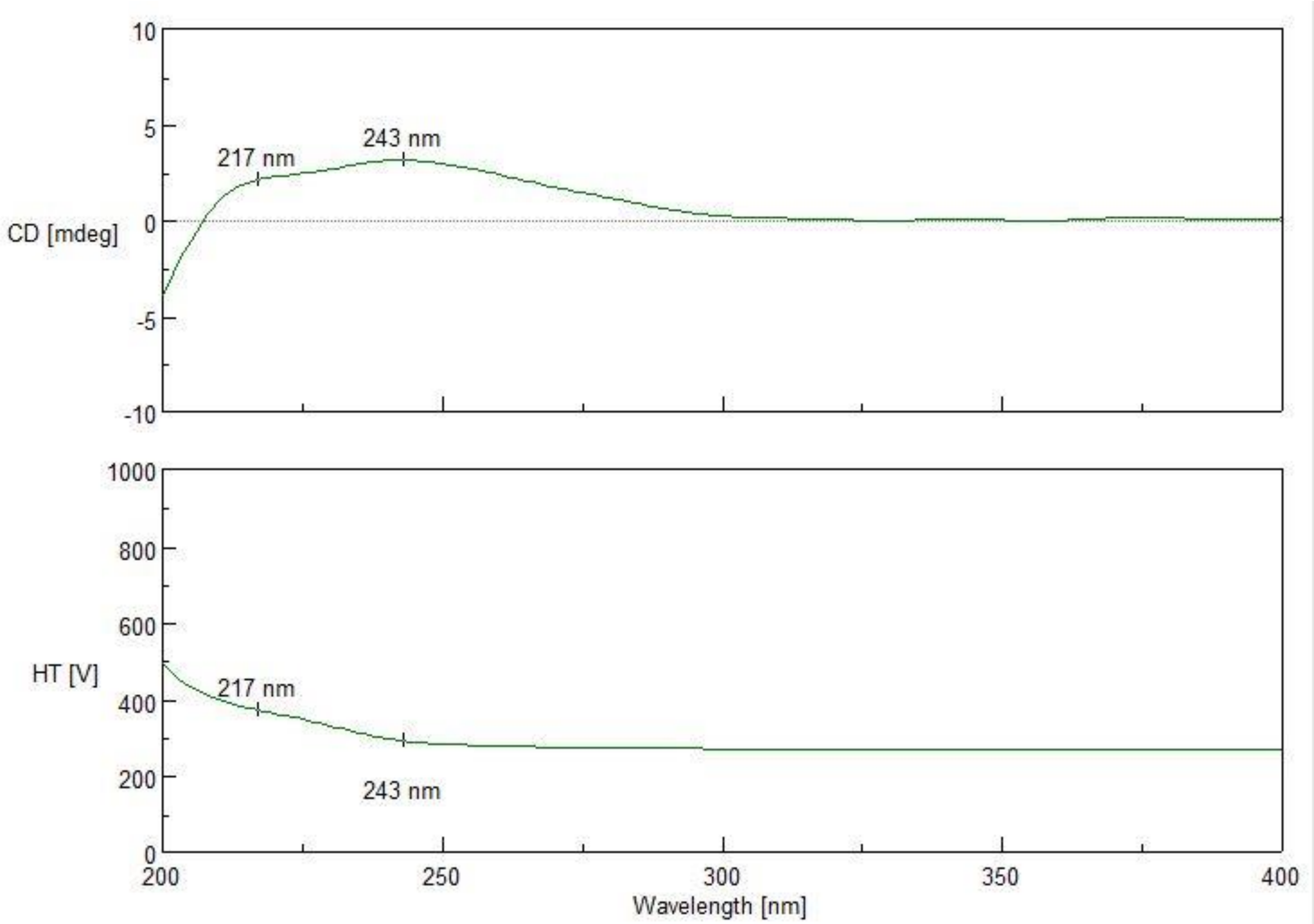

Figure S28. HRESIMS data of **4**

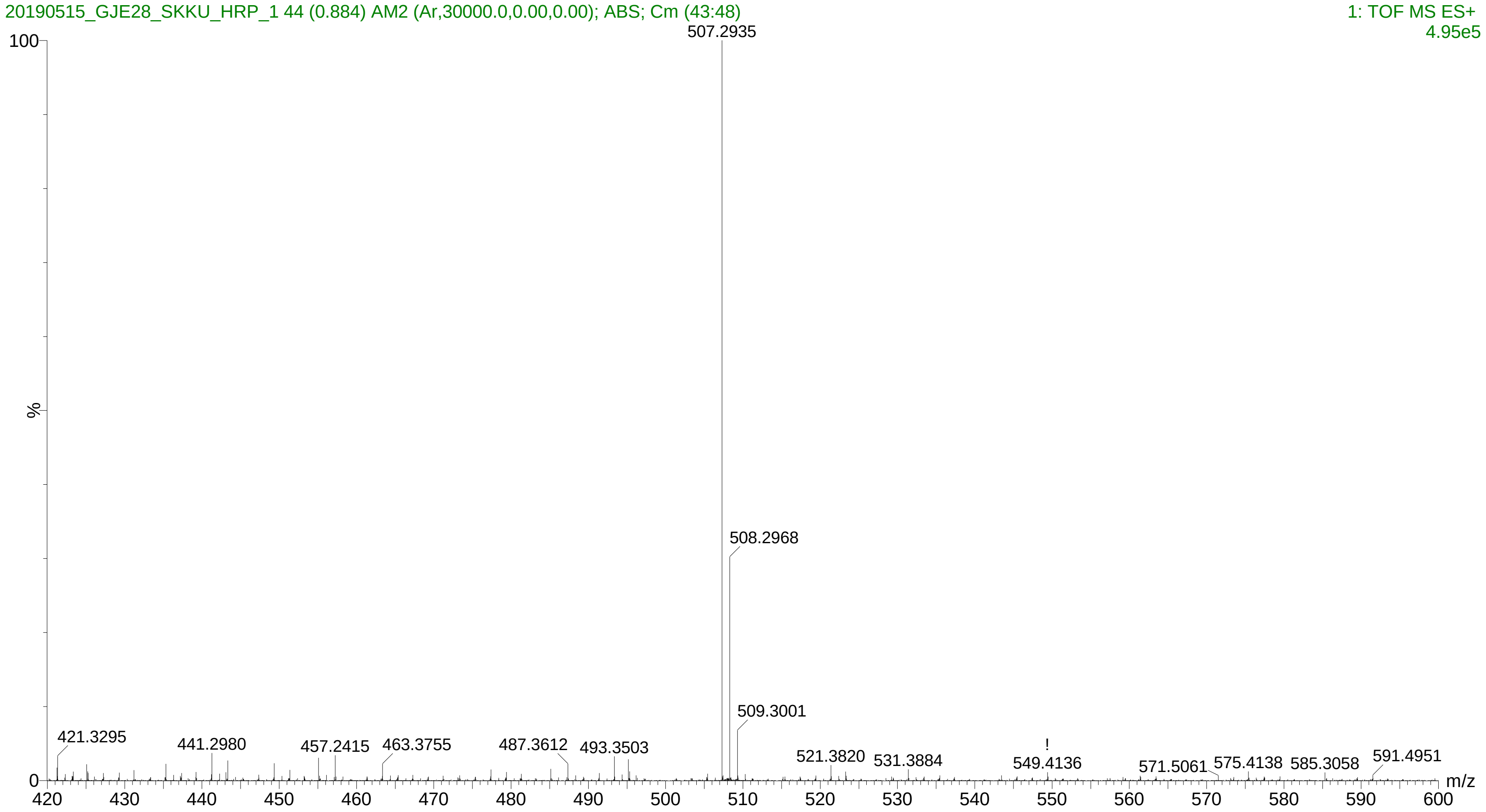

Figure S29. ^{1}H NMR data of **4** in CD_3OD

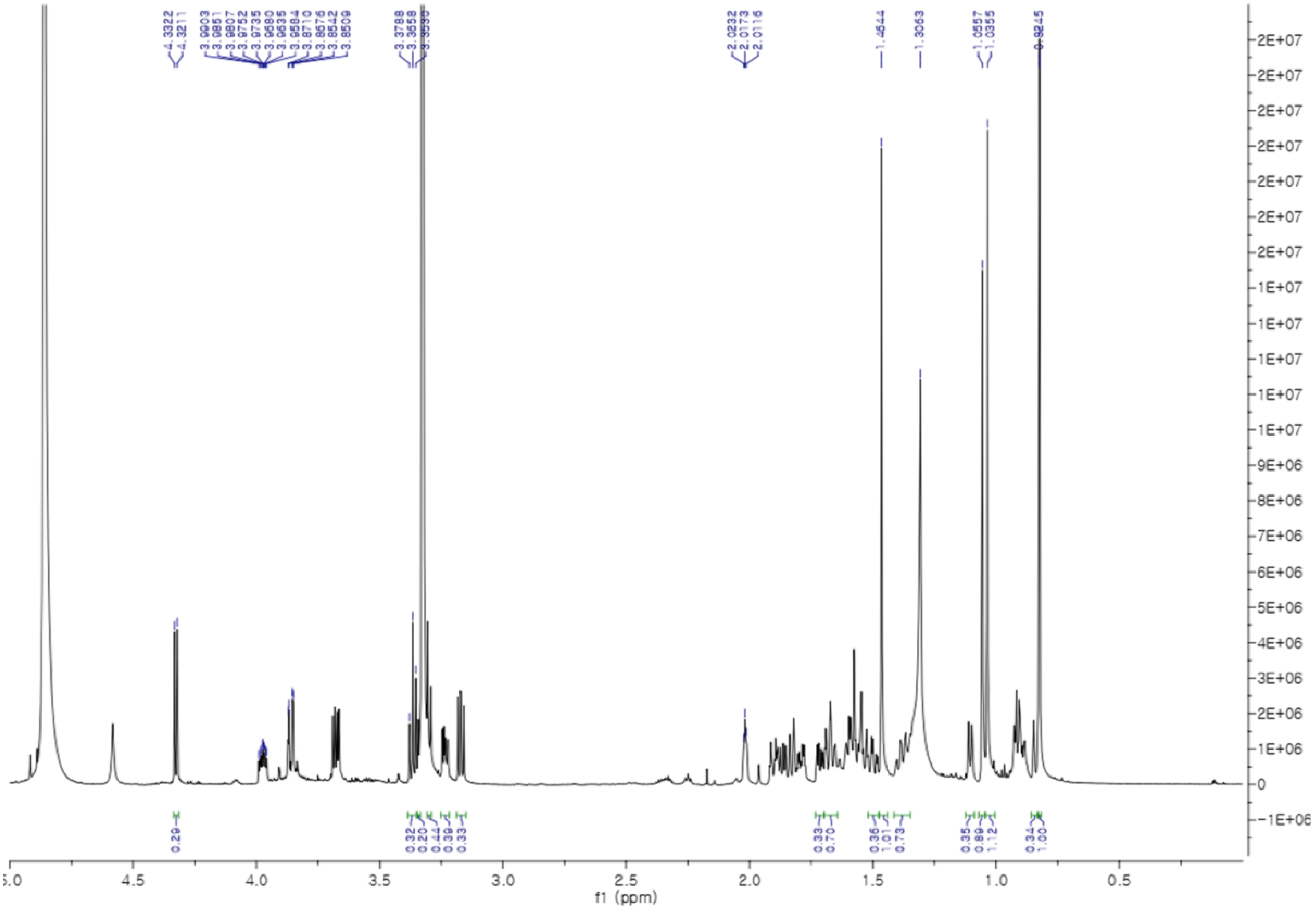

Figure S30. ^{13}C NMR data of **4** in CD_3OD

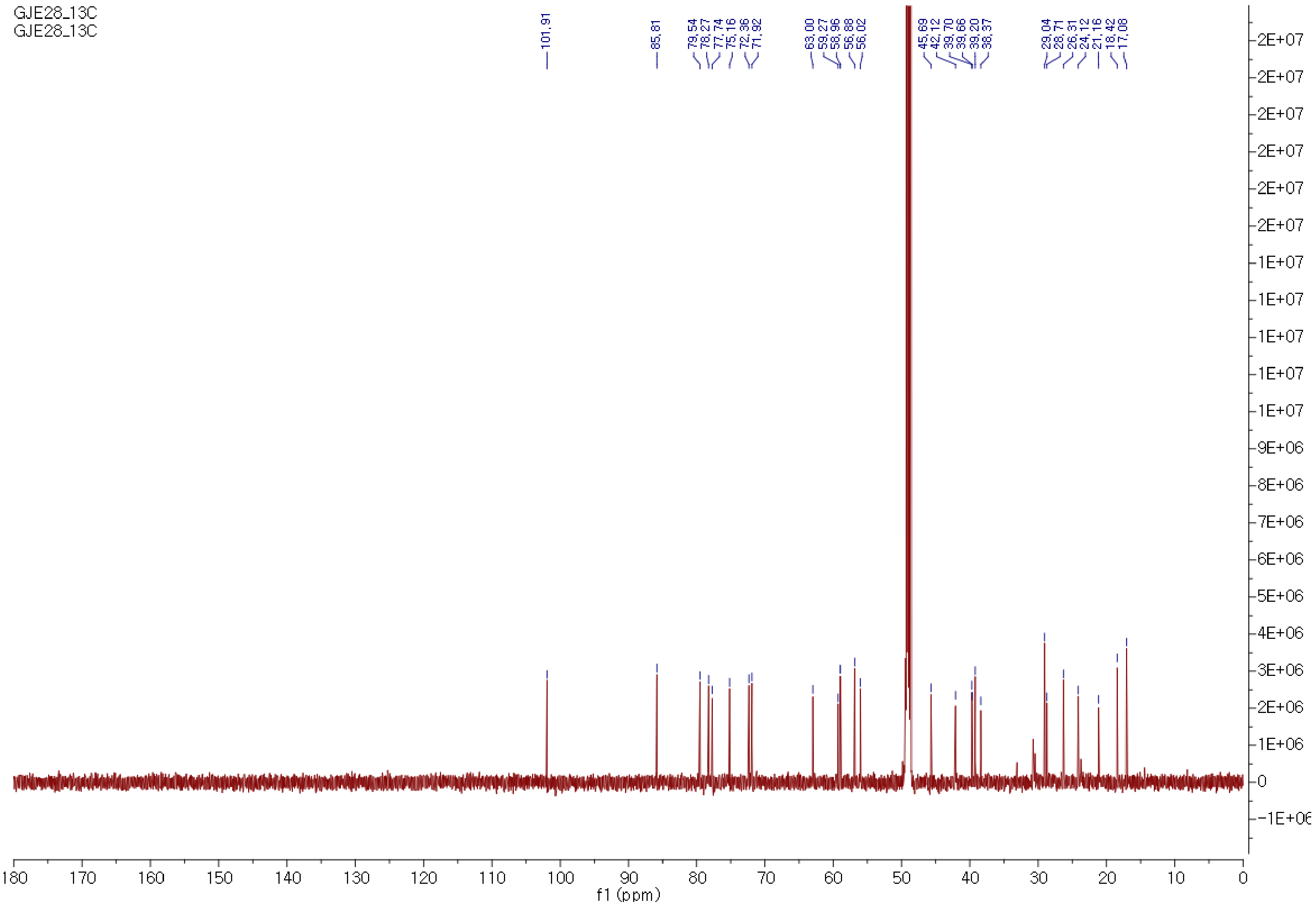

Figure S31. ^{1}H-^{1}H COSY spectrum of **4** in CD_3OD

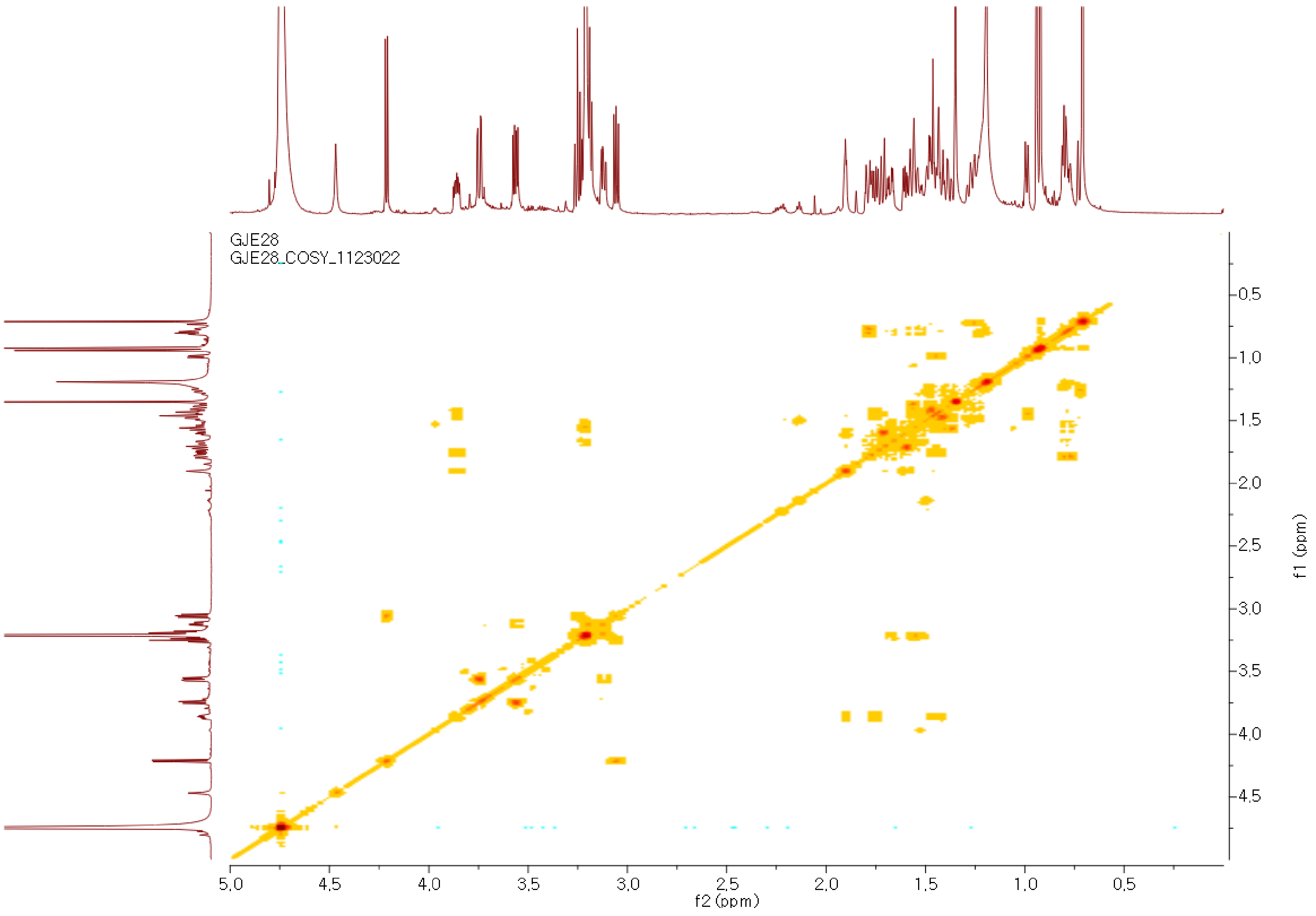

Figure S32. HSQC spectrum of **4** in CD_3OD

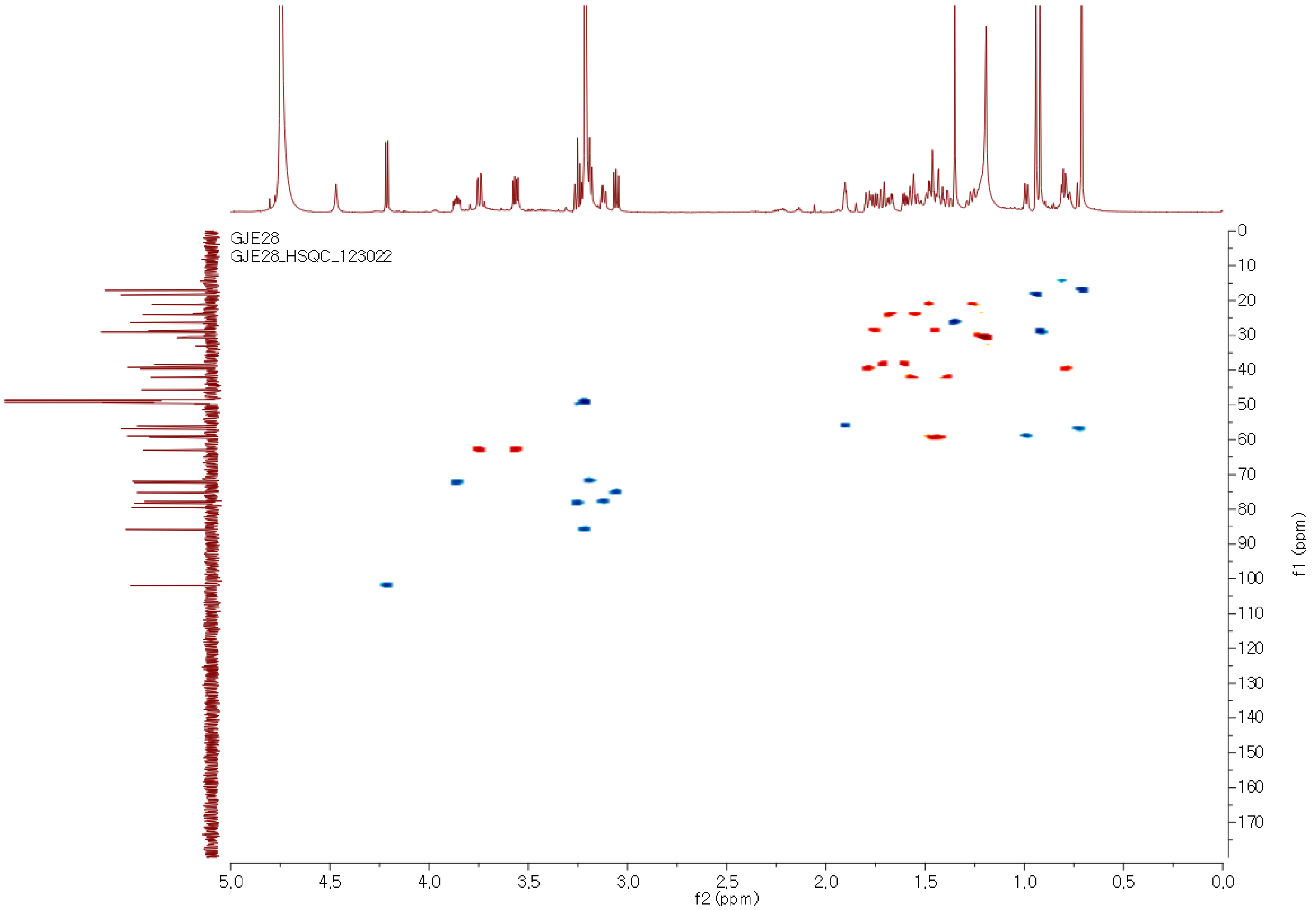

Figure S33. HMBC spectrum of **4** in CD_3OD

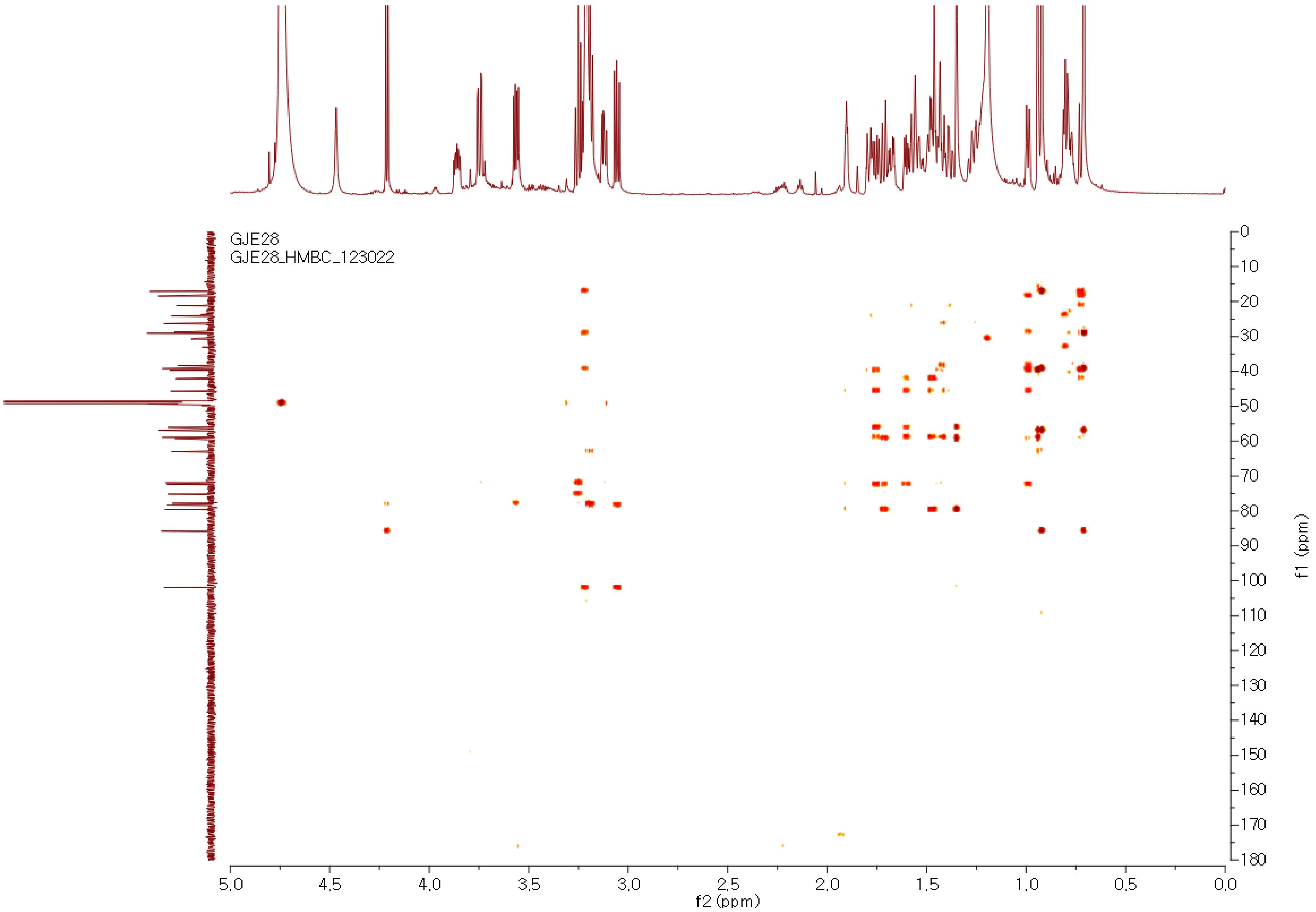

Figure S34. NOESY spectrum of **4** in CD_3OD

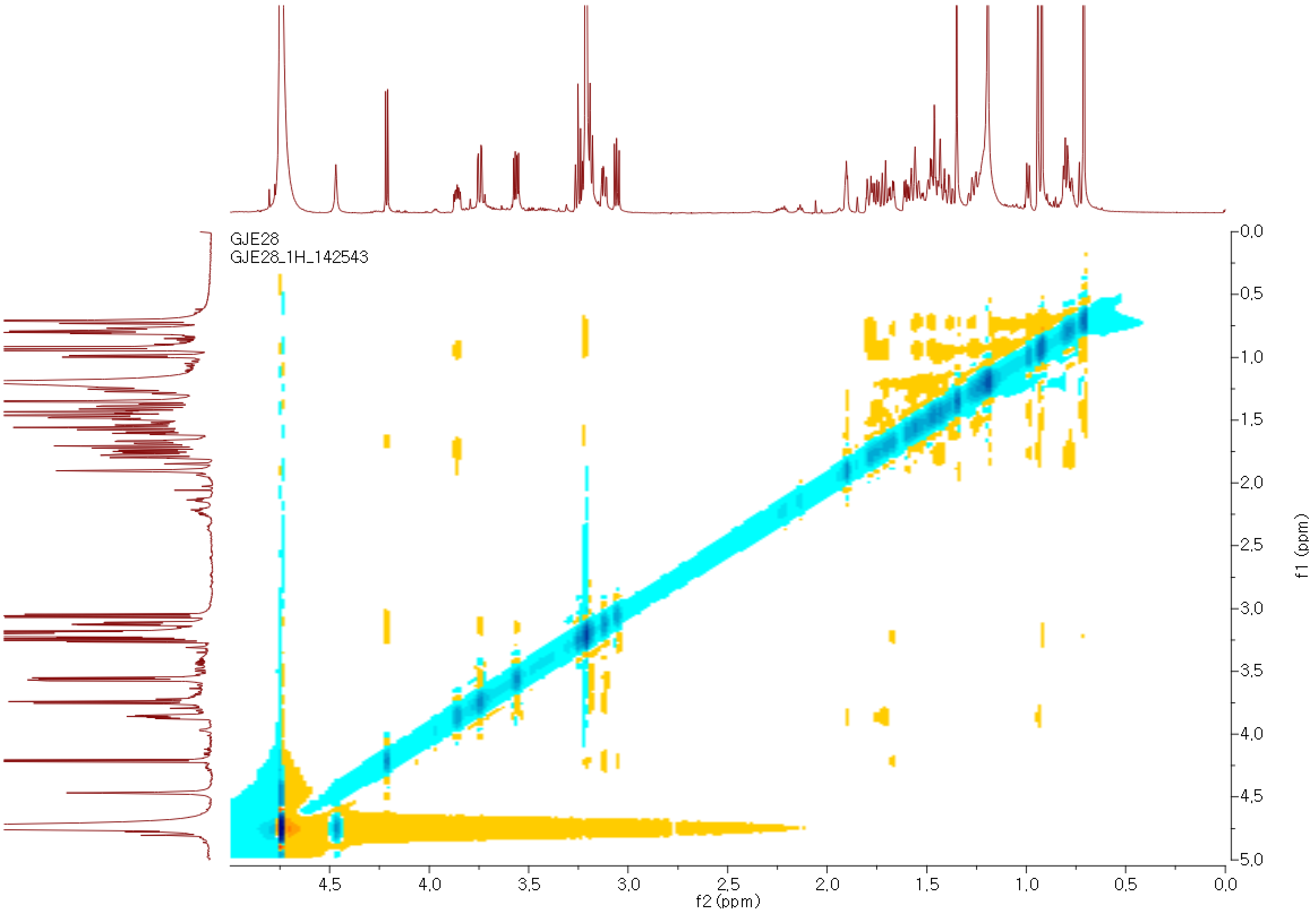

Figure S35. Comparison of standard D-glucopyranose and monosaccharide obtained by hydrolysis of **4**

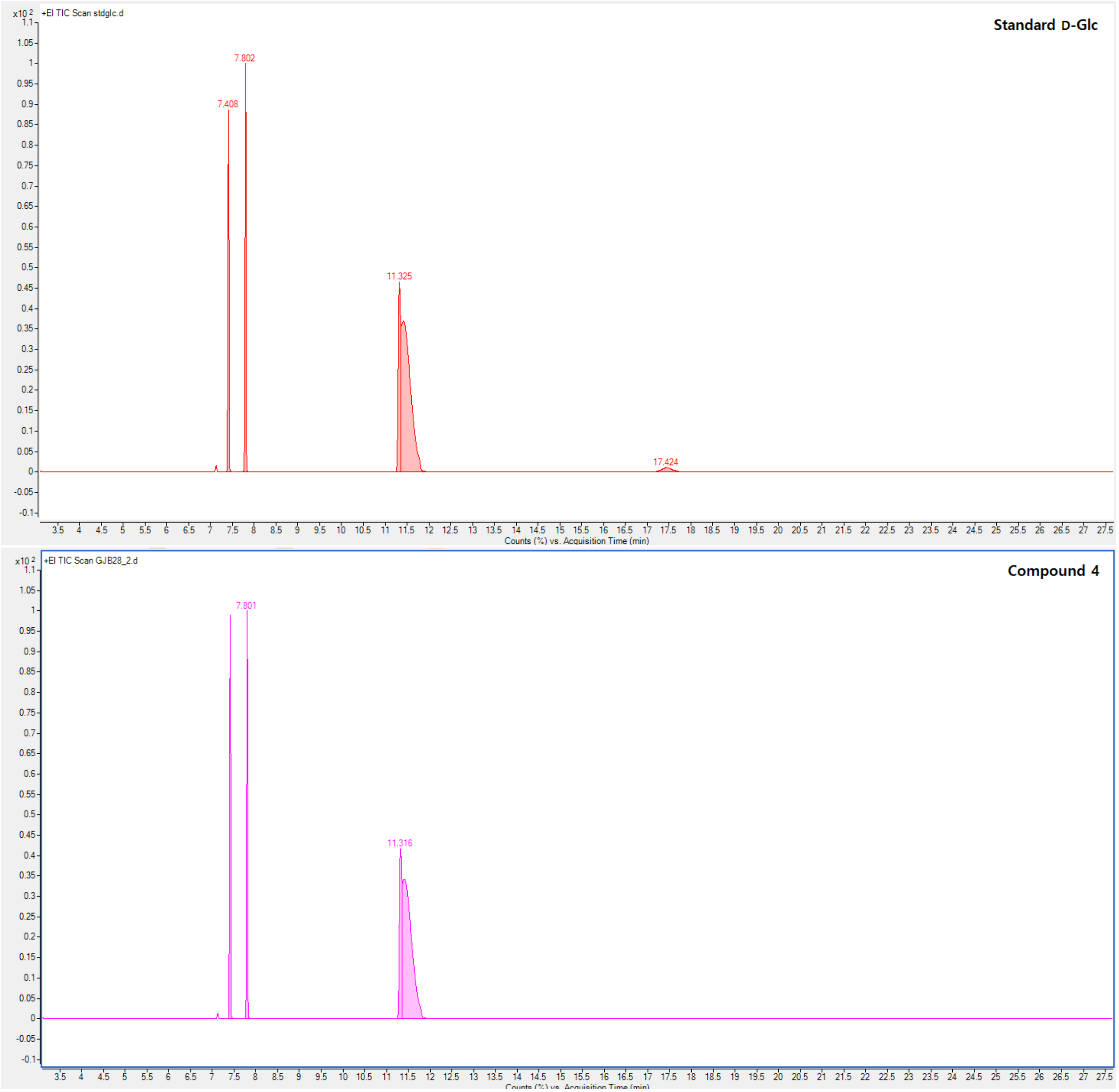

Figure S36. HRESIMS data of **5**

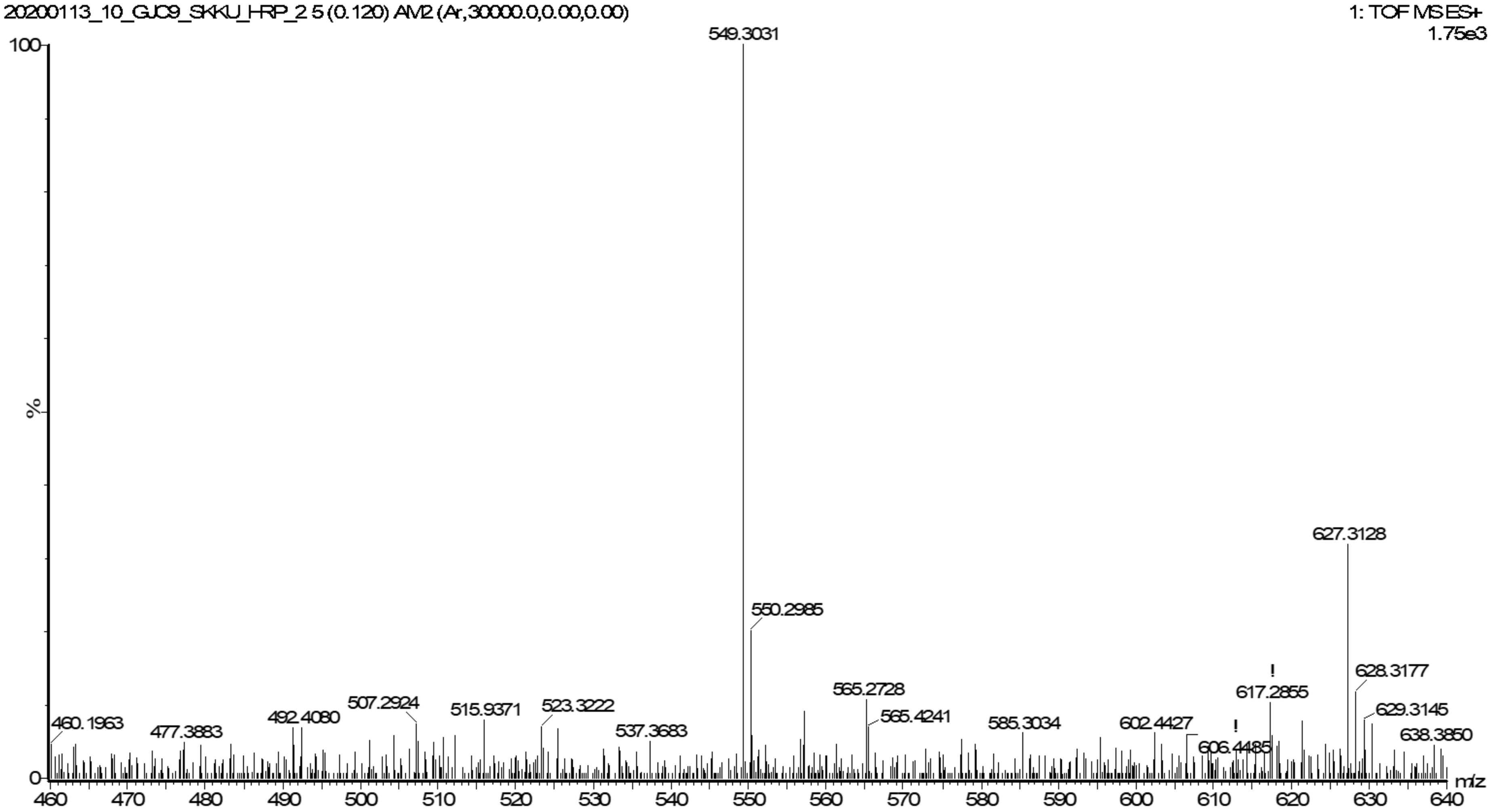

Figure S37. ^{1}H NMR data of **5** in CD_3OD

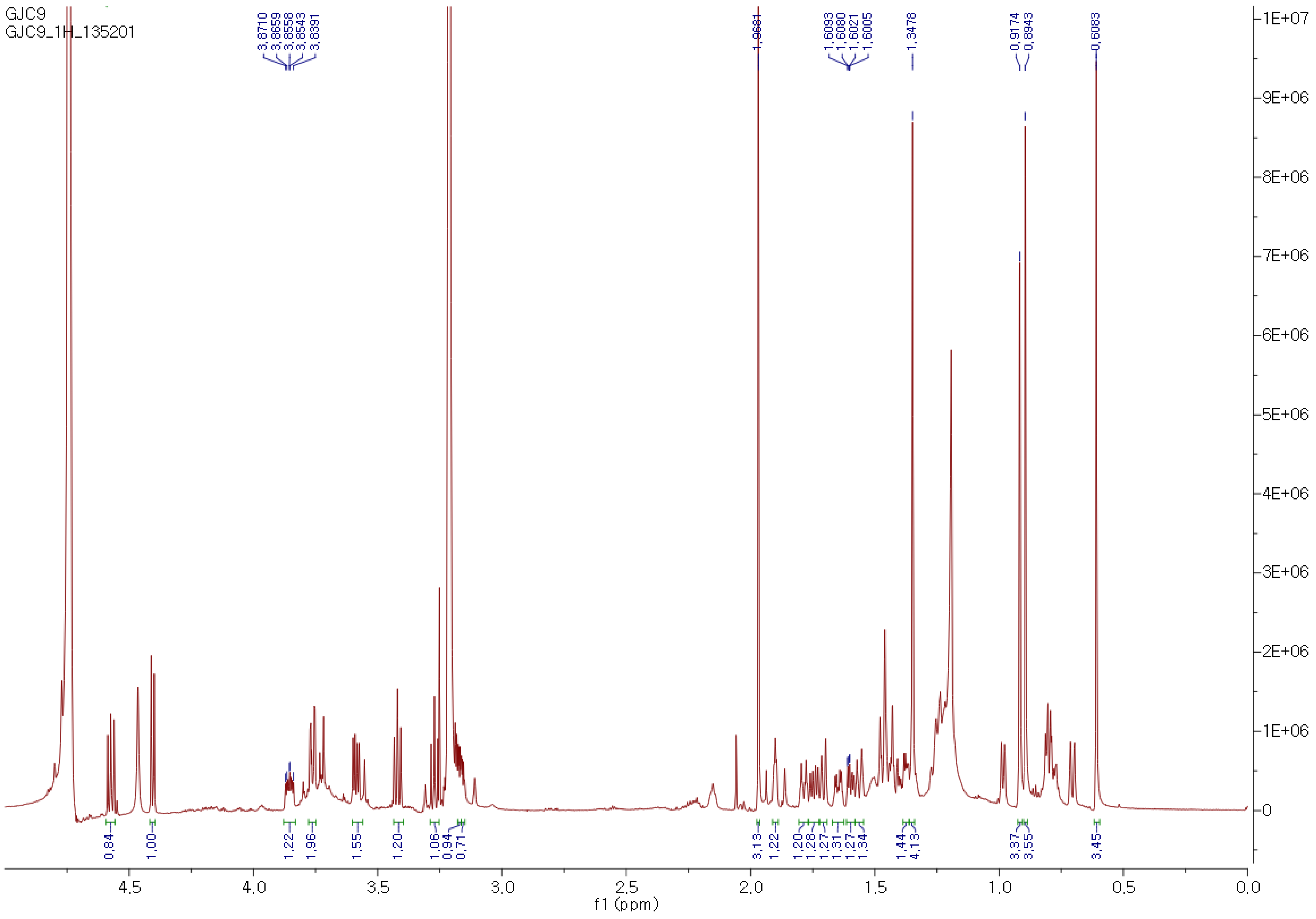

Figure S38. ^{13}C NMR data of **5** in CD_3OD

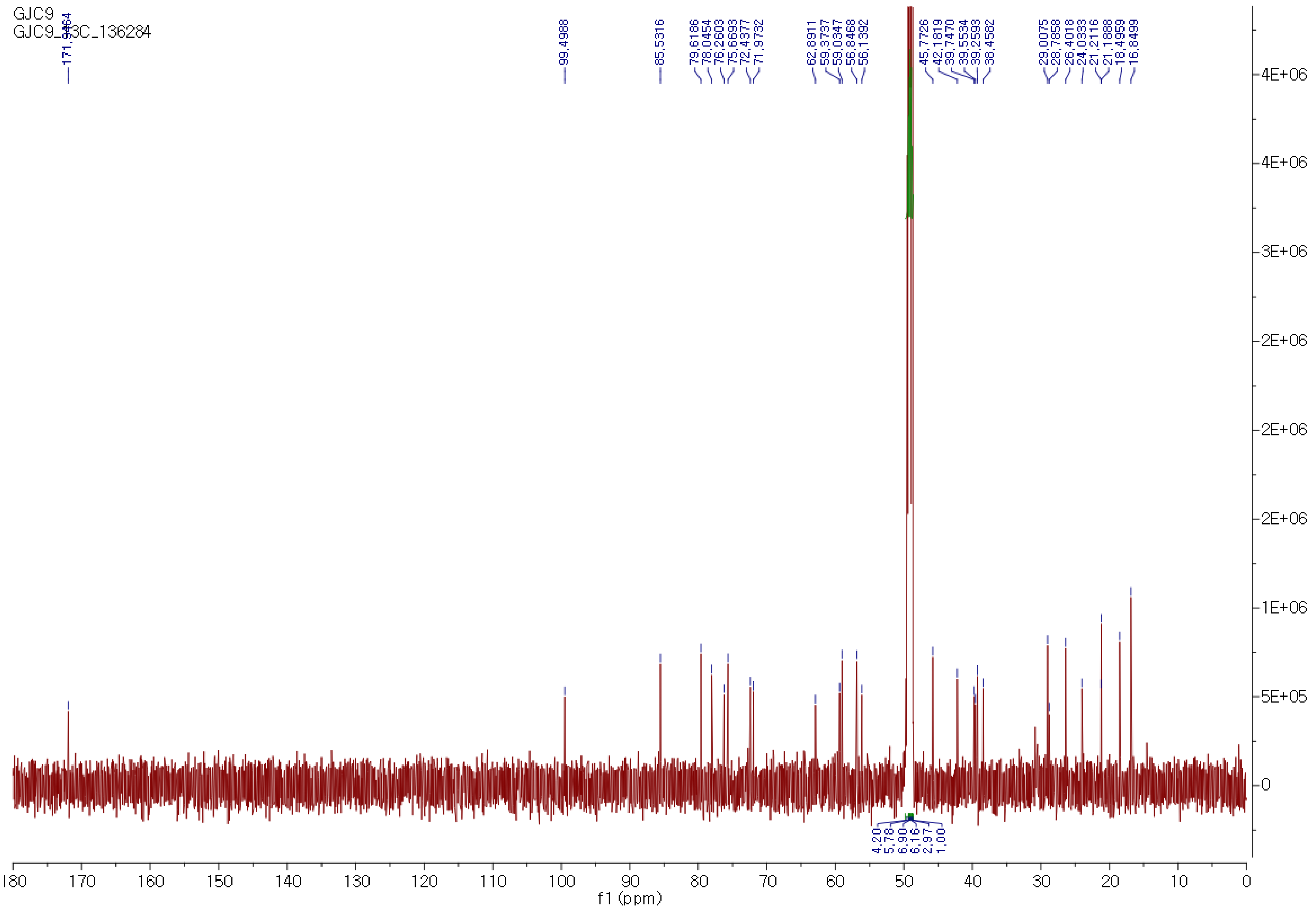

Figure S39. ^{1}H-^{1}H COSY spectrum of **5** in CD_3OD

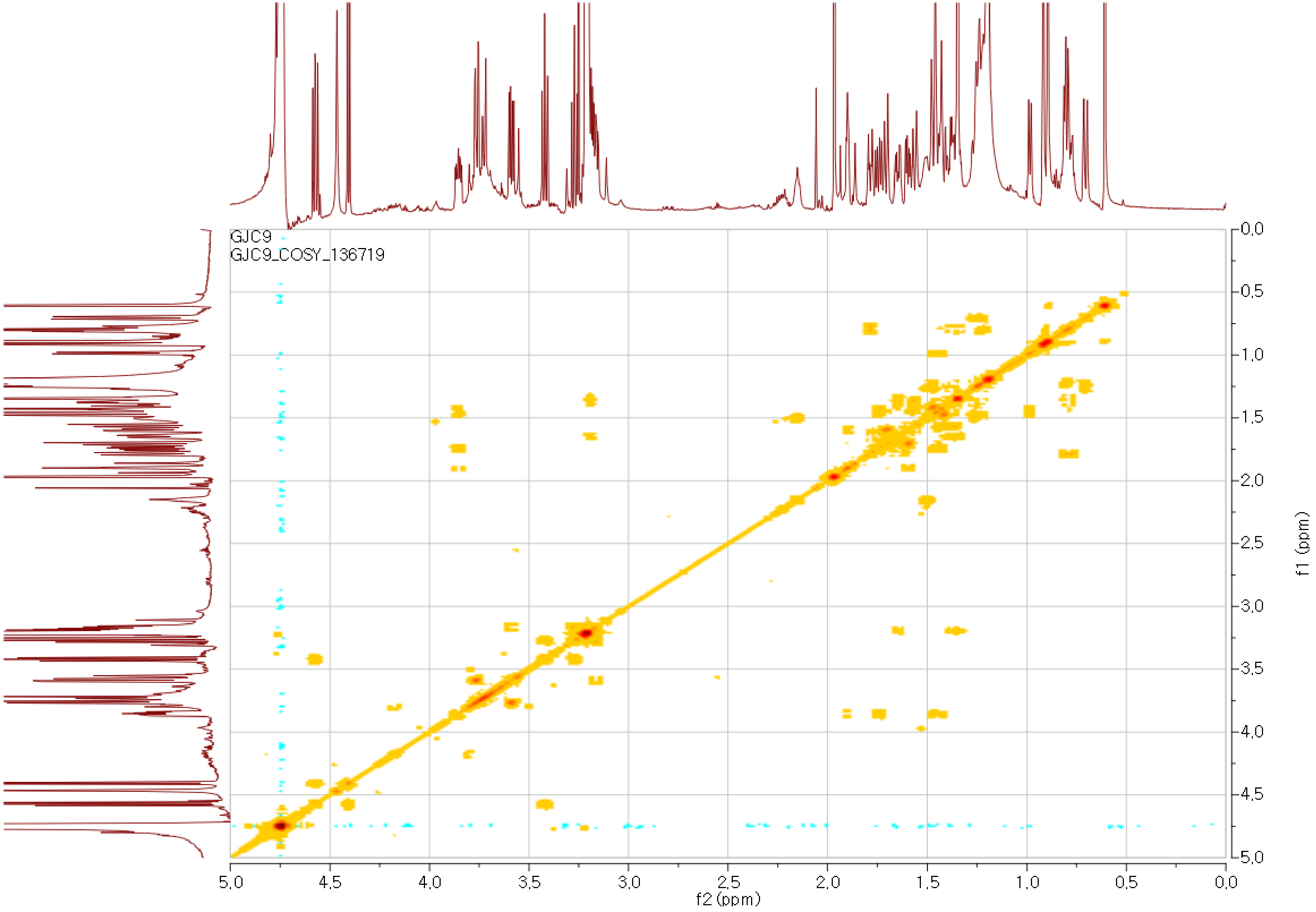

Figure S40. HSQC spectrum of **5** in CD_3OD

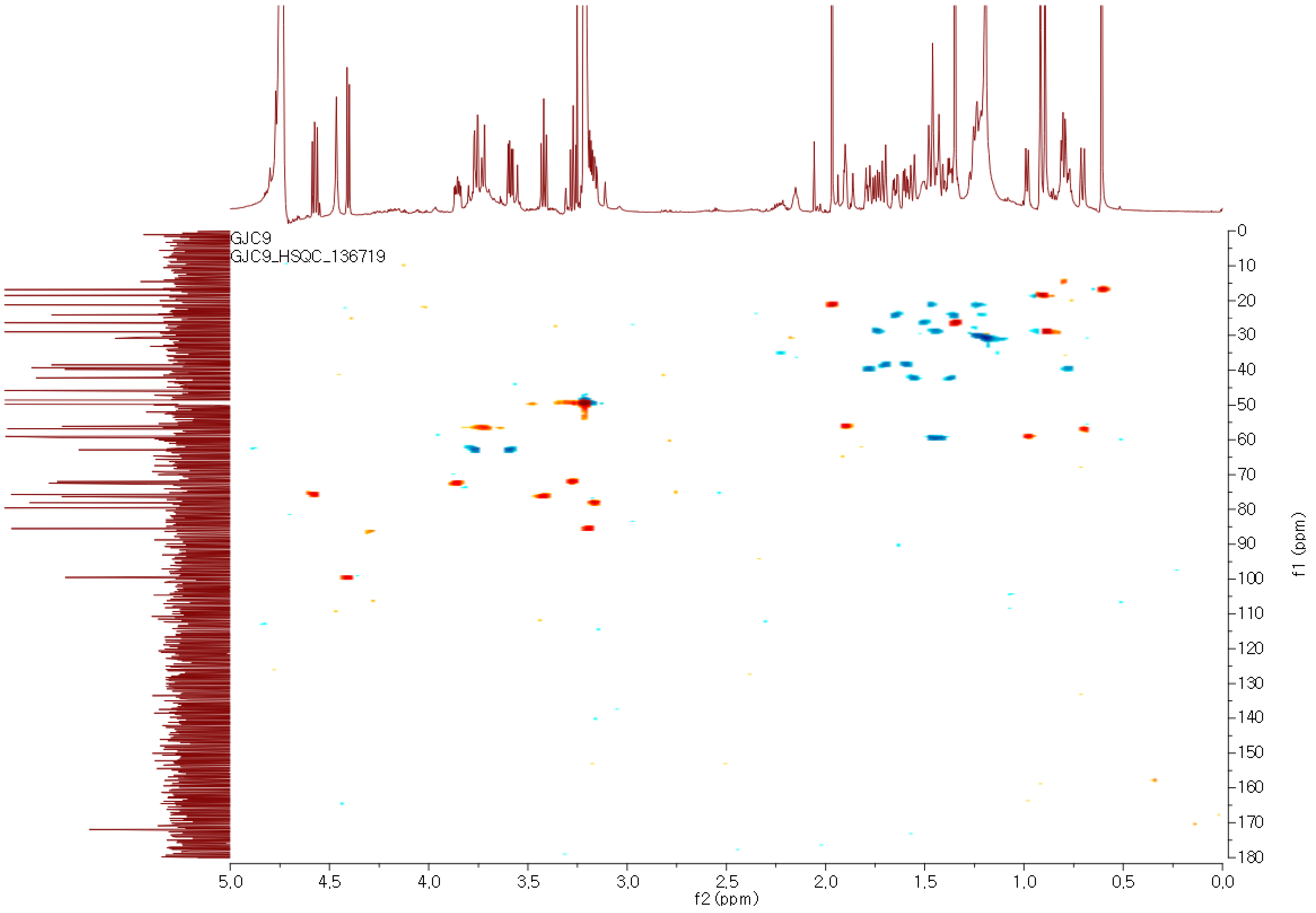

Figure S41. HMBC spectrum of **5** in CD_3OD

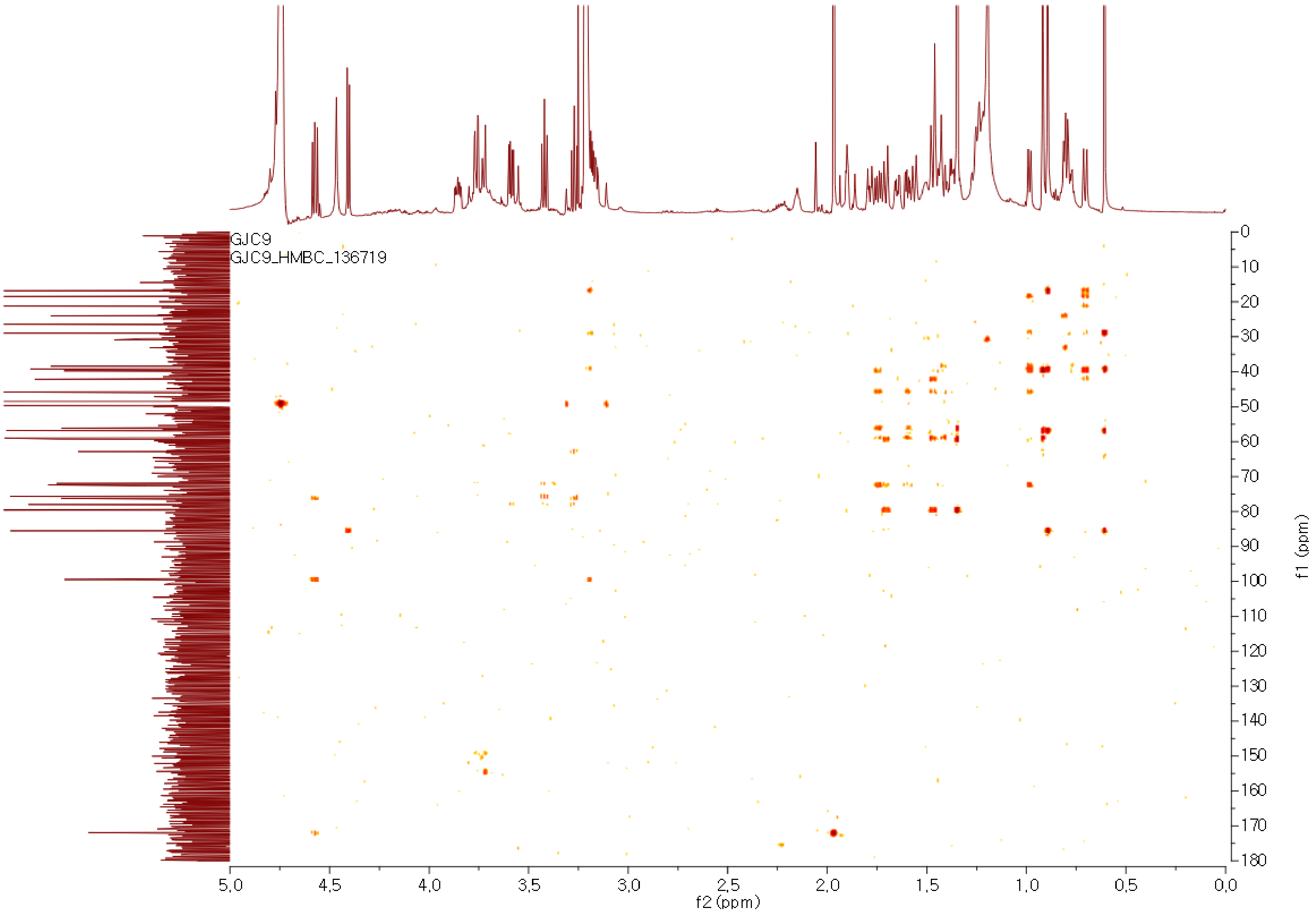

Figure S42. NOESY spectrum of **5** in CD_3OD

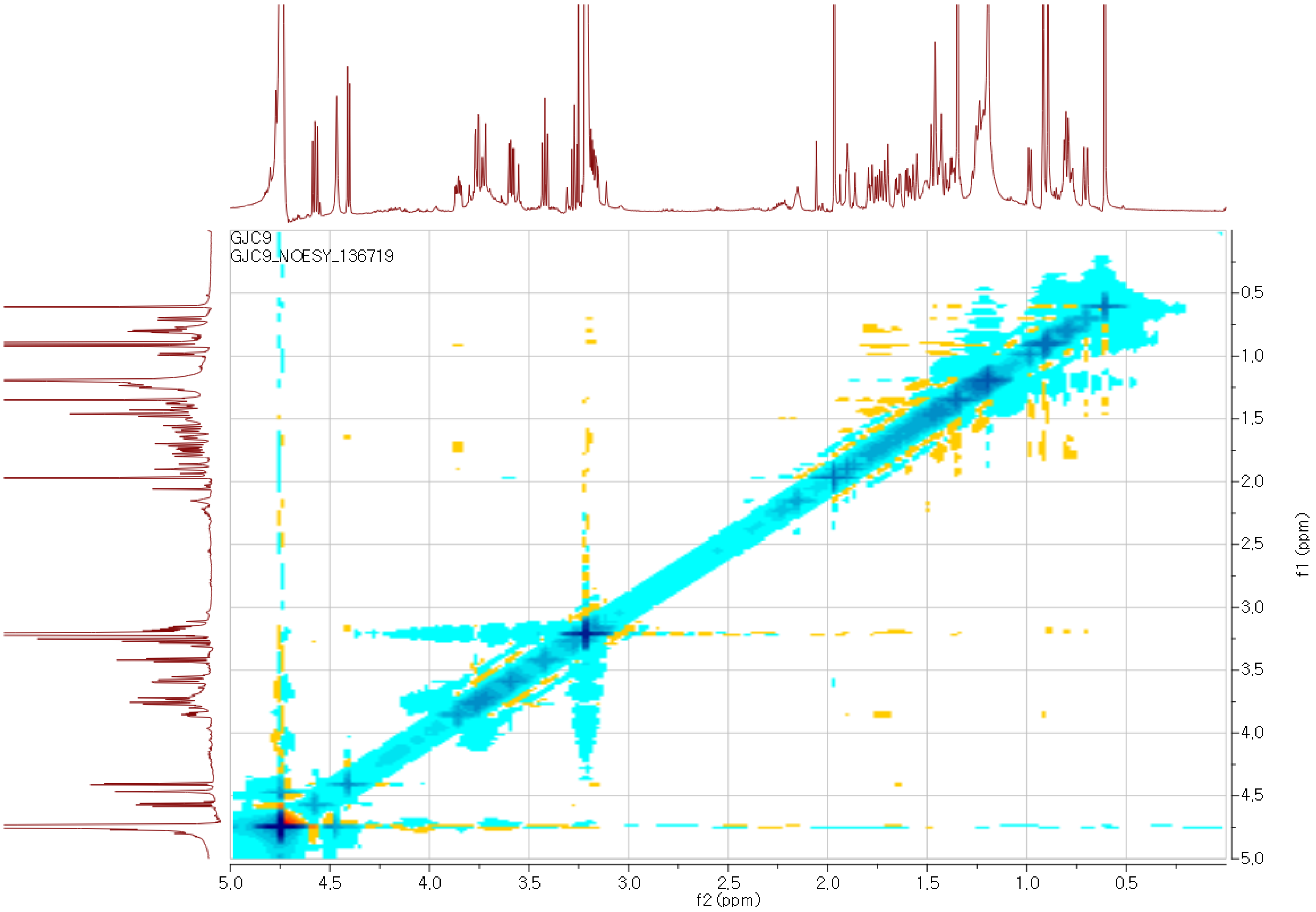

Figure S43. Comparison of standard D-glucopyranose and monosaccharide obtained by hydrolysis of **5**

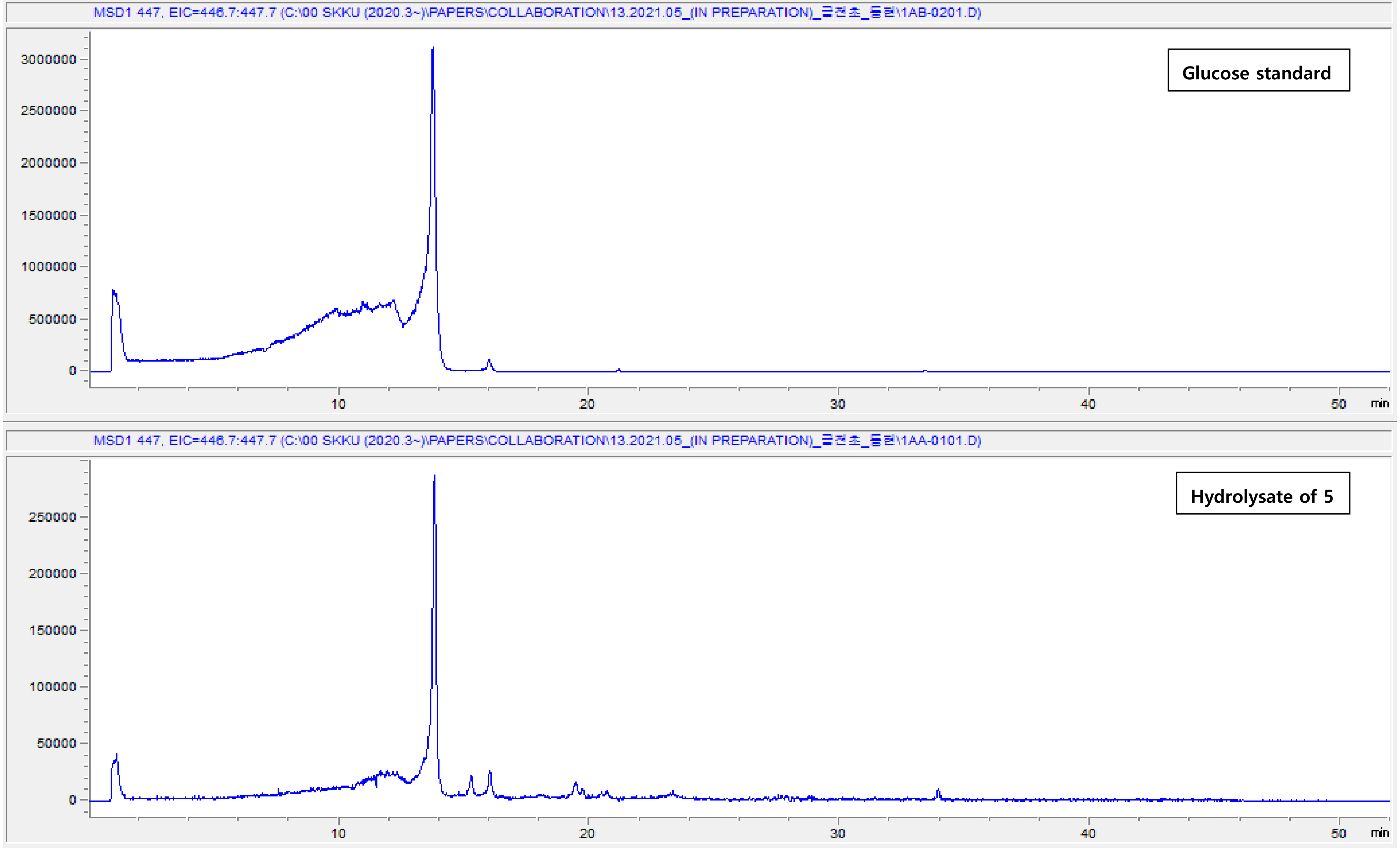

Figure S44. Optimized geometries of conformers of compound **1** at B3LYP/6-31G(d)

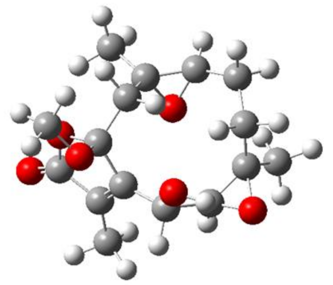

1-1

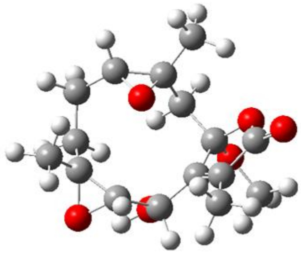

1-2

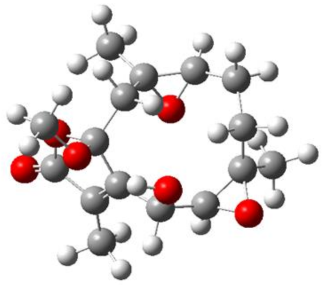

1-3

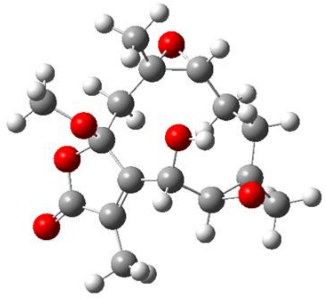

1-4

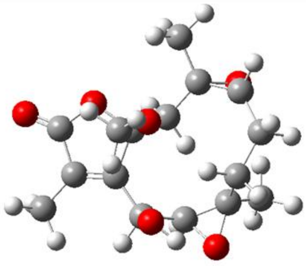

1-5

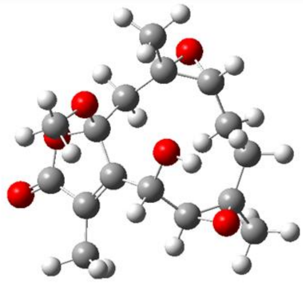

1-6

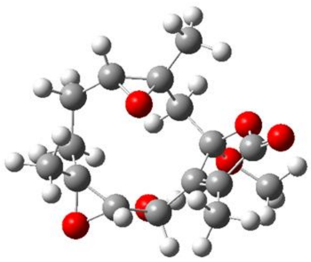

1-7

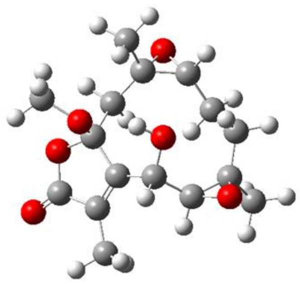

1-8

Figure S45. Optimized geometries of conformers of compound **2** (Boltzmann populations in parenthesis) at B3LYP/6-31G(d)

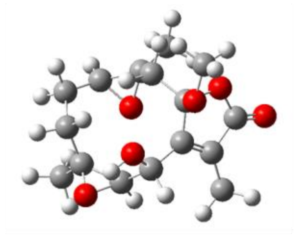

2-S1 (3%)

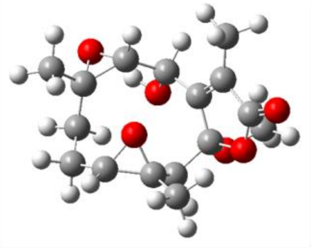

2-S2 (90%)

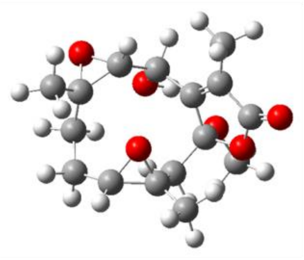

2-S3 (7%)

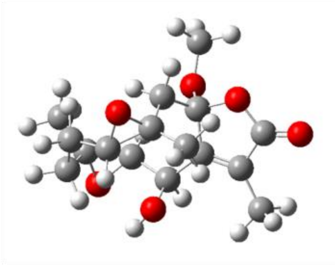

2-R1 (100%)

Figure S46. Optimized geometries of conformers of compound **3** at B3LYP/6-31G(d)

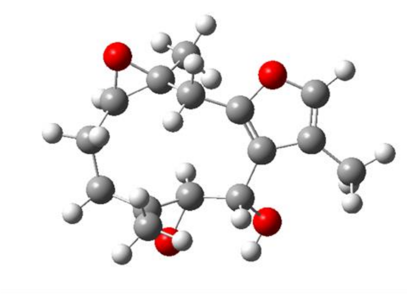

3-1

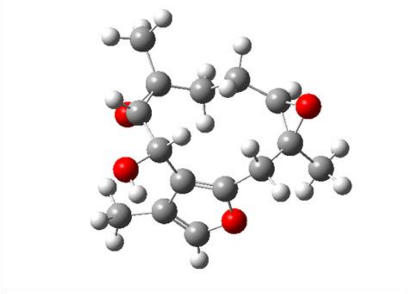

3-2

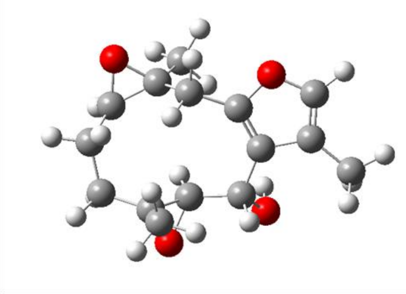

3-3

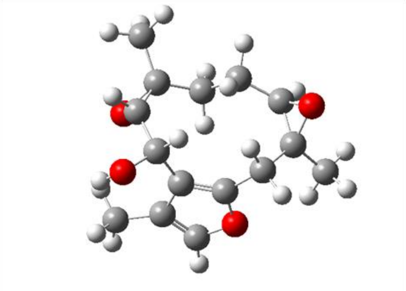

3-4

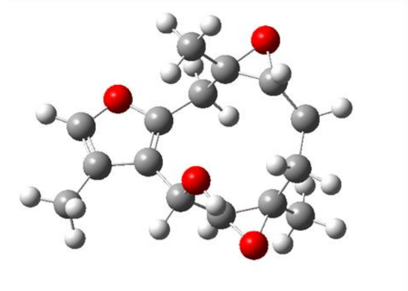

3-5

Table S1. Sum of electronic and zero-point energies (ZPE), thermal energies (E), thermal enthalpies (H), thermal free energies(G) calculated at B3LYP/6-31G(d) level for individual conformers of compound **1** with the use of an PCM model of methanol.

Conf. No.	ZPE	E	H	G[1]
**1-1**	-1073.832668	-1073.811182	-1073.810237	-1073.880849
**1-2**	-1073.836547	-1073.815102	-1073.814158	-1073.884525
**1-3**	-1073.832955	-1073.811470	-1073.810526	-1073.881005
**1-4**	-1073.826337	-1073.804740	-1073.803796	-1073.874776
**1-5**	-1073.830070	-1073.808671	-1073.807727	-1073.878025
**1-6**	-1073.830322	-1073.808683	-1073.807739	-1073.879002
**1-7**	-1073.833881	-1073.812427	-1073.811483	-1073.881827
**1-8**	-1073.828153	-1073.806609	-1073.805665	-1073.876326

[1]The thermal free energies (G) were used for calculation of Boltzmann distribution.

Table S2. Sum of electronic and zero-point energies (ZPE), thermal energies (E), thermal enthalpies (H), thermal free energies(G) calculated at B3LYP/6-31G(d) level for individual conformers of compound 2 with the use of an PCM model of methanol.

Conf. No.	ZPE	E	H	G
**2-R1**	-1073.831135	-1073.809463	-1073.808519	-1073.880062
**2-S1**	-1073.811182	-1073.810237	-1073.880849	-1073.811182
**2-S2**	-1073.811182	-1073.810237	-1073.880849	-1073.811182
**2-S3**	-1073.811182	-1073.810237	-1073.880849	-1073.811182

[1]The thermal free energies (G) were used for calculation of Boltzmann distribution.

Table S3. Sum of electronic and zero-point energies (ZPE), thermal energies (E), thermal enthalpies (H), thermal free energies(G) calculated at B3LYP/6-31G(d) level for individual conformers of compound 3 with the use of an PCM model of methanol.

Conf. No.	ZPE	E	H	G
**3-1**	-884.107937	-884.089940	-884.088995	-884.088995
**3-2**	-884.108768	-884.090758	-884.089814	-884.152661
**3-3**	-884.104834	-884.086596	-884.085651	-884.149989
**3-4**	-884.106951	-884.088951	-884.088007	-884.150907
**3-5**	-884.106059	-884.087989	-884.087045	-884.151121

[1]The thermal free energies (G) were used for calculation of Boltzmann distribution.

Table S4. Experimental (Exp.) and calculated (Cal.) ^{13}C chemical shift values of **2** and its possible isomers **2a** and **2b** used for DP4+ analysis.

Carbon	Exp.	Cal.	
	**2**	**2a**	**2b**
**1**	70.7	66.9	64.0
**2**	23.7	28.5	26.3
**3**	38.2	29.4	30.9
**4**	62.3	68.0	67.5
**5**	65.7	65.0	67.9
**6**	76.6	68.7	71.0
**7**	151.4	158.7	160.4
**8**	110.9	109.0	110.9
**9**	45.3	37.8	40.3
**10**	57.9	60.4	58.8
**11**	136.9	136.6	129.5
**12**	171.9	169.41	168.52
**13**	10.2	12.35	12.44
**14**	17.2	23.34	26.01
**15**	17.7	24.45	24.08
**8-OCH$_3$**	51.3	52.34	51.77

Table S5. Coordinates of the conformers 1-1 – 1-8.

1-1

Center Number	Atomic Number	Atomic Type	Coordinates (Angstroms) X	Y	Z
1	8	0	-3.396661	-0.667399	-2.027154
2	6	0	-2.559948	-0.614402	-1.160467
3	6	0	-1.414773	-1.522029	-0.910615
4	6	0	-1.185850	-2.706445	-1.795017
5	6	0	-0.740753	-1.074440	0.159321
6	6	0	0.472238	-1.727722	0.772808
7	8	0	0.695678	-1.285798	2.098408
8	6	0	1.737249	-1.589135	-0.089872
9	6	0	2.764443	-0.533791	-0.055899
10	8	0	2.956455	-1.808195	0.643031
11	6	0	3.657411	-0.397597	-1.274420
12	6	0	2.721439	0.674138	0.873173
13	6	0	2.522769	2.045397	0.197390
14	6	0	1.328423	2.267097	-0.723842
15	6	0	-0.113253	2.030410	-0.477197
16	8	0	0.664963	1.137748	-1.315642
17	6	0	-1.092185	2.902615	-1.247046
18	6	0	-0.635008	1.449708	0.834927
19	6	0	-1.461478	0.151001	0.724117
20	8	0	-1.992430	-0.252561	1.957695
21	6	0	-2.905058	0.633831	2.601118
22	8	0	-2.544433	0.377640	-0.209232

23	1	0	0.267930	-2.814327	0.786997
24	1	0	1.664020	-2.160228	-1.018005
25	1	0	1.526971	3.062928	-1.448230
26	1	0	-2.144298	-3.092877	-2.154559
27	1	0	-0.600662	-2.428587	-2.680846
28	1	0	-0.650737	-3.509059	-1.276325
29	1	0	1.607355	-1.572403	2.296147
30	1	0	3.777711	-1.366033	-1.768089
31	1	0	4.652677	-0.036880	-0.987558
32	1	0	3.227106	0.306002	-1.994090
33	1	0	1.982456	0.502999	1.652616
34	1	0	3.698124	0.712439	1.376652
35	1	0	2.497957	2.814588	0.982215
36	1	0	3.418130	2.269671	-0.396121
37	1	0	-1.343539	3.796734	-0.662710
38	1	0	-0.650185	3.223444	-2.194927
39	1	0	-2.019664	2.365399	-1.456799
40	1	0	-1.275155	2.211545	1.292661
41	1	0	0.167008	1.245516	1.543156
42	1	0	-2.395850	1.491274	3.060393
43	1	0	-3.674583	0.989263	1.907800
44	1	0	-3.375350	0.045310	3.391904

1-2

Center Number	Atomic Number	Atomic Type	Coordinates (Angstroms) X	Y	Z
1	8	0	3.484816	0.523902	1.878385
2	6	0	2.643730	0.185062	1.081702
3	6	0	1.615944	-0.877286	1.211862
4	6	0	1.539870	-1.705330	2.455011
5	6	0	0.875637	-0.890119	0.092431
6	6	0	-0.256253	-1.843791	-0.195201
7	8	0	-0.533452	-1.907884	-1.581922
8	6	0	-1.526266	-1.578320	0.629853
9	6	0	-2.676615	-0.717402	0.308844
10	8	0	-2.709092	-2.161554	0.052910
11	6	0	-3.578652	-0.311558	1.458766
12	6	0	-2.780877	0.135247	-0.949451
13	6	0	-2.746035	1.663161	-0.736387
14	6	0	-1.592277	2.289389	0.041083
15	6	0	-0.134266	2.158506	-0.185916
16	8	0	-0.793442	1.469017	0.909214
17	6	0	0.744143	3.317766	0.253841
18	6	0	0.425351	1.281472	-1.298623
19	6	0	1.406944	0.171347	-0.881548
20	8	0	1.884116	-0.332098	-2.093629
21	6	0	2.775309	-1.440850	-2.035848
22	8	0	2.510743	0.769823	-0.149815
23	1	0	0.081393	-2.840281	0.147485

24	1	0	-1.380294	-1.812397	1.686643
25	1	0	-1.887750	3.232883	0.509641
26	1	0	2.539478	-1.831847	2.881395
27	1	0	0.927546	-1.207981	3.217908
28	1	0	1.104096	-2.692463	2.266394
29	1	0	-1.408897	-2.336306	-1.635399
30	1	0	-3.576758	-1.082364	2.234606
31	1	0	-4.611208	-0.180219	1.113242
32	1	0	-3.239408	0.628616	1.904784
33	1	0	-2.028094	-0.187013	-1.665783
34	1	0	-3.755789	-0.095304	-1.402342
35	1	0	-2.800945	2.147860	-1.721254
36	1	0	-3.667424	1.958117	-0.218707
37	1	0	0.888226	4.019389	-0.577047
38	1	0	0.276653	3.855489	1.084083
39	1	0	1.729879	2.970022	0.571547
40	1	0	0.979716	1.915747	-1.998862
41	1	0	-0.361586	0.796907	-1.873753
42	1	0	3.138196	-1.577869	-3.056578
43	1	0	3.628264	-1.240000	-1.377351
44	1	0	2.261064	-2.354395	-1.714320

1-3

Center Number	Atomic Number	Atomic Type	Coordinates (Angstroms) X	Y	Z
1	8	0	-3.431301	-0.602321	-2.017655
2	6	0	-2.576491	-0.570691	-1.170765
3	6	0	-1.441334	-1.495518	-0.939139
4	6	0	-1.246014	-2.690343	-1.817047
5	6	0	-0.748869	-1.057242	0.122185
6	6	0	0.428048	-1.710304	0.817732
7	8	0	0.640166	-1.203476	2.123629
8	6	0	1.706478	-1.632958	-0.023829
9	6	0	2.762460	-0.604972	-0.039686
10	8	0	2.943432	-1.878486	0.640680
11	6	0	3.604410	-0.483440	-1.296970
12	6	0	2.773205	0.600211	0.892388
13	6	0	2.583820	1.978236	0.221447
14	6	0	1.408653	2.227641	-0.720508
15	6	0	-0.043663	2.033341	-0.499058
16	8	0	0.717986	1.119611	-1.324155
17	6	0	-0.995090	2.927091	-1.277344
18	6	0	-0.587102	1.462683	0.807169
19	6	0	-1.439094	0.184489	0.682808
20	8	0	-1.941340	-0.227001	1.952224
21	6	0	-2.865040	0.645949	2.609404
22	8	0	-2.534694	0.421967	-0.206519
23	1	0	0.201997	-2.790622	0.882041

24	1	0	1.576459	-2.195999	-0.953226
25	1	0	1.639050	3.016080	-1.443492
26	1	0	-2.215732	-3.084352	-2.136363
27	1	0	-0.694176	-2.420687	-2.726233
28	1	0	-0.688380	-3.483331	-1.308369
29	1	0	-0.240854	-1.077183	2.521228
30	1	0	3.667154	-1.449994	-1.805381
31	1	0	4.625003	-0.167411	-1.047224
32	1	0	3.176193	0.245139	-1.992544
33	1	0	2.063849	0.426737	1.698959
34	1	0	3.766135	0.621551	1.363830
35	1	0	2.556284	2.745614	1.008393
36	1	0	3.487428	2.199598	-0.360632
37	1	0	-1.240355	3.823043	-0.693292
38	1	0	-0.533700	3.243962	-2.217155
39	1	0	-1.929306	2.407824	-1.504689
40	1	0	-1.209105	2.236037	1.271398
41	1	0	0.215555	1.231708	1.506595
42	1	0	-3.306107	0.055789	3.415326
43	1	0	-2.366490	1.521968	3.042104
44	1	0	-3.651166	0.967144	1.920372

1-4

Center Number	Atomic Number	Atomic Type	Coordinates (Angstroms) X	Y	Z
1	8	0	-3.273283	-2.405748	-1.482347
2	6	0	-2.496968	-1.698489	-0.889050
3	6	0	-1.270574	-2.082650	-0.148981
4	6	0	-0.911631	-3.532463	-0.030602
5	6	0	-0.711675	-0.974100	0.366883
6	6	0	0.491603	-0.969629	1.282200
7	8	0	0.731013	0.314538	1.820064
8	6	0	1.733135	-1.581547	0.621392
9	6	0	2.899141	-0.913205	0.001988
10	8	0	2.954012	-1.435605	1.361981
11	6	0	3.715741	-1.722003	-0.988837
12	6	0	3.059978	0.592450	-0.126426
13	6	0	2.180562	1.245921	-1.224871
14	6	0	1.383852	2.390663	-0.635090
15	6	0	-0.068269	2.417364	-0.369045
16	8	0	0.517212	3.145328	-1.486731
17	6	0	-0.570932	3.313337	0.743280
18	6	0	-1.020387	1.345866	-0.889981
19	6	0	-1.597206	0.237059	0.022598
20	8	0	-2.144216	0.679295	1.236296
21	6	0	-3.378616	1.396676	1.169605
22	8	0	-2.647434	-0.343536	-0.796895
23	1	0	0.249675	-1.665948	2.107379

24	1	0	1.550164	-2.605727	0.292499
25	1	0	1.989846	3.023242	0.022153
26	1	0	-1.817846	-4.136372	-0.133644
27	1	0	-0.227566	-3.847492	-0.828603
28	1	0	-0.438209	-3.764441	0.929382
29	1	0	1.540671	0.223405	2.351327
30	1	0	3.565247	-2.796455	-0.849647
31	1	0	4.782750	-1.505806	-0.860782
32	1	0	3.442113	-1.462465	-2.018568
33	1	0	2.832088	1.047556	0.837959
34	1	0	4.119845	0.798480	-0.320519
35	1	0	2.804703	1.644240	-2.033855
36	1	0	1.517215	0.508032	-1.687825
37	1	0	-0.724860	2.735732	1.657835
38	1	0	0.151928	4.111219	0.937847
39	1	0	-1.519407	3.785832	0.461326
40	1	0	-0.569713	0.847892	-1.752352
41	1	0	-1.888806	1.880303	-1.294987
42	1	0	-3.578888	1.722157	2.192459
43	1	0	-3.315261	2.278113	0.521542
44	1	0	-4.190645	0.752832	0.819756

1-5

Center Number	Atomic Number	Atomic Type	Coordinates (Angstroms) X	Y	Z
1	8	0	-4.186705	1.139435	-0.796736
2	6	0	-3.110060	0.689608	-0.491300
3	6	0	-2.670519	-0.736382	-0.497335
4	6	0	-3.639697	-1.795928	-0.910752
5	6	0	-1.395920	-0.786898	-0.078118
6	6	0	-0.568356	-2.035593	0.128345
7	8	0	-0.309337	-2.207436	1.514139
8	6	0	0.730939	-2.132848	-0.694181
9	6	0	2.075485	-1.598857	-0.360848
10	8	0	1.710105	-2.990318	-0.089884
11	6	0	3.077651	-1.470931	-1.491851
12	6	0	2.359809	-0.779968	0.901204
13	6	0	3.090868	0.575421	0.772299
14	6	0	2.453387	1.853428	0.226500
15	6	0	1.159972	2.137930	-0.436326
16	8	0	2.393093	2.068980	-1.197186
17	6	0	0.569536	3.527598	-0.313030
18	6	0	0.189770	1.047204	-0.850768
19	6	0	-0.891909	0.642172	0.173228
20	8	0	-0.403669	0.900079	1.451353
21	6	0	-1.290242	0.637472	2.545948
22	8	0	-2.070386	1.465028	-0.059936
23	1	0	-1.177823	-2.882815	-0.226131

24	1	0	0.535937	-2.346809	-1.748698
25	1	0	2.875042	2.723863	0.738114
26	1	0	-4.553212	-1.722534	-0.310129
27	1	0	-3.943036	-1.647208	-1.953941
28	1	0	-3.231410	-2.803888	-0.806211
29	1	0	0.470689	-2.795253	1.549507
30	1	0	2.842257	-2.173541	-2.296538
31	1	0	4.088093	-1.696545	-1.130738
32	1	0	3.080163	-0.452344	-1.893931
33	1	0	1.446460	-0.645939	1.478924
34	1	0	3.025017	-1.412894	1.505801
35	1	0	3.392747	0.827351	1.796870
36	1	0	4.032681	0.445469	0.222521
37	1	0	0.088777	3.821291	-1.253419
38	1	0	-0.189764	3.550399	0.474781
39	1	0	1.346249	4.262211	-0.081071
40	1	0	0.772297	0.177110	-1.131745
41	1	0	-0.334704	1.373190	-1.756153
42	1	0	-0.752179	0.965569	3.437460
43	1	0	-2.213394	1.217157	2.446441
44	1	0	-1.509970	-0.430554	2.626197

1-6

Center Number	Atomic Number	Atomic Type	Coordinates (Angstroms) X	Y	Z
1	8	0	-3.764850	-1.299472	-1.728480
2	6	0	-2.853398	-0.863091	-1.067783
3	6	0	-1.790947	-1.617561	-0.354056
4	6	0	-1.813500	-3.114853	-0.364876
5	6	0	-0.978104	-0.739490	0.258704
6	6	0	0.146417	-1.133407	1.188723
7	8	0	0.629621	-0.017073	1.909338
8	6	0	1.257256	-1.940135	0.503475
9	6	0	2.581791	-1.503180	0.009773
10	8	0	2.427823	-2.143131	1.310623
11	6	0	3.269588	-2.385734	-1.014892
12	6	0	3.061904	-0.064331	0.034859
13	6	0	2.495750	0.800462	-1.120872
14	6	0	1.963112	2.103855	-0.568807
15	6	0	0.542248	2.450256	-0.355225
16	8	0	1.313473	3.015674	-1.456340
17	6	0	0.212444	3.455789	0.729166
18	6	0	-0.605299	1.613740	-0.914400
19	6	0	-1.471565	0.697016	-0.022348
20	8	0	-1.837466	1.416740	1.108540
21	6	0	-2.722707	0.786934	2.030019
22	8	0	-2.663893	0.465635	-0.840419
23	1	0	-0.293610	-1.855385	1.904101

24	1	0	0.868455	-2.860540	0.063304
25	1	0	2.671548	2.593910	0.107872
26	1	0	-2.838626	-3.461324	-0.524200
27	1	0	-1.209738	-3.519786	-1.186869
28	1	0	-1.433688	-3.541212	0.570091
29	1	0	1.384957	-0.353478	2.423020
30	1	0	2.885016	-3.409244	-0.982783
31	1	0	4.347303	-2.418386	-0.817185
32	1	0	3.125663	-1.990801	-2.027430
33	1	0	2.778626	0.382213	0.987976
34	1	0	4.158573	-0.071609	0.004188
35	1	0	3.275116	1.025230	-1.859024
36	1	0	1.709461	0.264654	-1.662359
37	1	0	-0.676544	4.037808	0.462611
38	1	0	0.011686	2.949337	1.675444
39	1	0	1.051694	4.146804	0.854894
40	1	0	-0.251234	1.018110	-1.759026
41	1	0	-1.318645	2.332763	-1.333826
42	1	0	-2.972297	1.552891	2.766684
43	1	0	-3.640609	0.449070	1.536854
44	1	0	-2.240695	-0.055615	2.540808

1-7

Center Number	Atomic Number	Atomic Type	Coordinates (Angstroms) X	Y	Z
1	8	0	3.429405	0.416258	1.957100
2	6	0	2.592139	0.115458	1.146180
3	6	0	1.580240	-0.968445	1.190585
4	6	0	1.514723	-1.897600	2.360957
5	6	0	0.849026	-0.901337	0.067221
6	6	0	-0.253452	-1.827323	-0.402668
7	8	0	-0.543197	-1.685895	-1.782770
8	6	0	-1.519043	-1.679038	0.450215
9	6	0	-2.684521	-0.797259	0.257933
10	8	0	-2.730023	-2.213197	-0.077153
11	6	0	-3.512784	-0.453922	1.482823
12	6	0	-2.851911	0.125836	-0.942670
13	6	0	-2.810431	1.640921	-0.647085
14	6	0	-1.652361	2.249230	0.139435
15	6	0	-0.191931	2.158254	-0.093972
16	8	0	-0.829829	1.417600	0.976230
17	6	0	0.664148	3.321452	0.379003
18	6	0	0.391111	1.324405	-1.228794
19	6	0	1.385179	0.226627	-0.812116
20	8	0	1.880663	-0.293130	-2.049439
21	6	0	3.084866	-1.065421	-1.988305
22	8	0	2.456748	0.792148	-0.054897
23	1	0	0.090362	-2.861446	-0.212085

24	1	0	-1.310516	-1.974800	1.483078
25	1	0	-1.957048	3.171596	0.643261
26	1	0	2.518276	-2.073272	2.760306
27	1	0	0.919552	-1.459063	3.171635
28	1	0	1.062395	-2.858261	2.094397
29	1	0	0.289859	-1.457936	-2.235385
30	1	0	-3.456281	-1.262363	2.217646
31	1	0	-4.566813	-0.320771	1.208883
32	1	0	-3.158322	0.467635	1.955163
33	1	0	-2.144549	-0.169540	-1.714565
34	1	0	-3.850141	-0.079179	-1.355175
35	1	0	-2.891442	2.181238	-1.601157
36	1	0	-3.720397	1.903857	-0.092726
37	1	0	0.795118	4.050317	-0.430441
38	1	0	0.185887	3.825511	1.223902
39	1	0	1.656011	2.983914	0.689485
40	1	0	0.937973	1.985272	-1.910212
41	1	0	-0.390260	0.839413	-1.811723
42	1	0	3.925822	-0.449290	-1.659091
43	1	0	2.984978	-1.931504	-1.321882
44	1	0	3.259412	-1.412628	-3.008339

1-8

Center Number	Atomic Number	Atomic Type	Coordinates (Angstroms) X	Y	Z
1	8	0	-3.475386	-2.179869	-1.468571
2	6	0	-2.621914	-1.556450	-0.891320
3	6	0	-1.402388	-2.047183	-0.205424
4	6	0	-1.141251	-3.519495	-0.118344
5	6	0	-0.751826	-0.992892	0.314016
6	6	0	0.432501	-1.022282	1.263278
7	8	0	0.724317	0.250036	1.805027
8	6	0	1.657954	-1.669915	0.616609
9	6	0	2.871151	-1.047013	0.046015
10	8	0	2.881048	-1.678136	1.350994
11	6	0	3.640178	-1.820901	-1.008814
12	6	0	3.082769	0.454430	0.032022
13	6	0	2.311744	1.154329	-1.118793
14	6	0	1.529261	2.338985	-0.601795
15	6	0	0.066159	2.423198	-0.410429
16	8	0	0.723342	3.078163	-1.529193
17	6	0	-0.454342	3.416381	0.611047
18	6	0	-0.912280	1.364227	-0.919312
19	6	0	-1.542442	0.286456	0.000763
20	8	0	-1.975788	0.770149	1.264943
21	6	0	-3.194295	1.523407	1.295131
22	8	0	-2.672150	-0.188576	-0.753740
23	1	0	0.157721	-1.713202	2.082562

24	1	0	1.392453	-2.648487	0.209420
25	1	0	2.121238	2.981547	0.057717
26	1	0	-2.087716	-4.062636	-0.194573
27	1	0	-0.507187	-3.864133	-0.944784
28	1	0	-0.647338	-3.794654	0.819325
29	1	0	-0.118494	0.611098	2.132332
30	1	0	3.427688	-2.892596	-0.950390
31	1	0	4.717564	-1.678594	-0.863065
32	1	0	3.389631	-1.470287	-2.017137
33	1	0	2.760538	0.846879	0.996789
34	1	0	4.158554	0.651408	-0.057613
35	1	0	3.004133	1.517701	-1.888235
36	1	0	1.642871	0.450176	-1.625452
37	1	0	-1.373370	3.893689	0.250208
38	1	0	-0.669971	2.936200	1.568981
39	1	0	0.286595	4.203944	0.773891
40	1	0	-0.462387	0.836569	-1.764335
41	1	0	-1.758798	1.916614	-1.346459
42	1	0	-3.289200	1.885071	2.320862
43	1	0	-3.165577	2.378876	0.612514
44	1	0	-4.047885	0.888983	1.043877

Table S6. Coordinates of the conformers 2-R1, 2-S1– 2-S3.

2-R1

Center Number	Atomic Number	Atomic Type	Coordinates (Angstroms) X	Y	Z
1	6	0	2.692520	1.557323	-0.584238
2	6	0	2.948052	0.072029	-0.939534
3	6	0	2.694253	-0.906478	0.201718
4	6	0	1.449345	-1.686376	0.401147
5	6	0	0.056806	2.066684	-0.509204
6	6	0	1.400446	2.131358	-1.131362
7	6	0	0.219192	-1.793765	-0.495457
8	6	0	-0.970100	-0.918947	-0.165610
9	6	0	-1.047728	0.139727	0.932851
10	6	0	-0.147570	1.391605	0.846004
11	6	0	-2.184389	-1.067645	-0.716265
12	6	0	-3.121270	-0.122358	-0.054576
13	8	0	-2.417383	0.597383	0.879757
14	8	0	0.877608	3.256004	-0.406637
15	8	0	2.607122	-2.306020	-0.192313
16	6	0	3.589944	-0.725319	1.417079
17	8	0	-4.297033	0.044919	-0.256089
18	6	0	-2.671395	-2.006483	-1.771543
19	8	0	-0.834885	-0.563579	2.138916
20	6	0	-1.153523	2.289202	-1.396849
21	6	0	-1.057446	0.153135	3.355737
22	8	0	0.541045	-1.633718	-1.874526

23	1	0	3.503313	2.172925	-0.993166
24	1	0	2.722675	1.724317	0.495965
25	1	0	2.345338	-0.209312	-1.804140
26	1	0	4.000566	-0.057017	-1.223741
27	1	0	1.241124	-2.008151	1.422242
28	1	0	1.407702	2.270381	-2.216718
29	1	0	-0.124584	-2.832207	-0.337158
30	1	0	0.822361	1.126745	1.271840
31	1	0	-0.592809	2.140222	1.511789
32	1	0	4.642782	-0.754366	1.112427
33	1	0	3.412701	0.231377	1.919434
34	1	0	3.422553	-1.528443	2.139937
35	1	0	-1.841610	-2.470379	-2.306960
36	1	0	-3.300684	-2.788461	-1.327405
37	1	0	-3.303024	-1.469769	-2.487791
38	1	0	-1.515330	1.355578	-1.835228
39	1	0	-1.971933	2.746210	-0.831337
40	1	0	-0.879261	2.966593	-2.210459
41	1	0	-1.025558	-0.597338	4.148403
42	1	0	-0.276477	0.900767	3.542290
43	1	0	-2.038686	0.637851	3.352643
44	1	0	1.354224	-2.159585	-1.995108

2-S1

Center Number	Atomic Number	Atomic Type	Coordinates (Angstroms) X	Y	Z
1	6	0	-2.522768	2.045397	0.197392
2	6	0	-2.721442	0.674135	0.873169
3	6	0	-2.764444	-0.533793	-0.055902
4	6	0	-1.737248	-1.589135	-0.089873
5	6	0	0.113251	2.030409	-0.477203
6	6	0	-1.328427	2.267093	-0.723848
7	6	0	-0.472237	-1.727720	0.772810
8	6	0	0.740754	-1.074440	0.159324
9	6	0	1.461478	0.151008	0.724117
10	6	0	0.635004	1.449713	0.834921
11	6	0	1.414778	-1.522028	-0.910610
12	6	0	2.559952	-0.614403	-1.160463
13	8	0	2.544432	0.377646	-0.209232
14	8	0	-0.664966	1.137744	-1.315644
15	8	0	-2.956454	-1.808198	0.643029
16	6	0	-3.657419	-0.397604	-1.274419
17	8	0	3.396669	-0.667405	-2.027145
18	6	0	1.185866	-2.706450	-1.795009
19	8	0	1.992427	-0.252552	1.957696
20	6	0	1.092181	2.902609	-1.247059
21	6	0	2.905052	0.633842	2.601121
22	8	0	-0.695677	-1.285796	2.098409
23	1	0	-2.497942	2.814582	0.982223

24	1	0	-3.418132	2.269684	-0.396109
25	1	0	-3.698127	0.712435	1.376648
26	1	0	-1.982461	0.502996	1.652613
27	1	0	-1.664013	-2.160228	-1.018006
28	1	0	-1.526978	3.062922	-1.448238
29	1	0	-0.267930	-2.814325	0.787000
30	1	0	-0.167010	1.245522	1.543153
31	1	0	1.275149	2.211553	1.292656
32	1	0	-3.777676	-1.366031	-1.768119
33	1	0	-4.652700	-0.036942	-0.987541
34	1	0	-3.227149	0.306036	-1.994069
35	1	0	2.144323	-3.092930	-2.154475
36	1	0	0.650681	-3.509032	-1.276341
37	1	0	0.600754	-2.428582	-2.680885
38	1	0	2.019647	2.365381	-1.456838
39	1	0	0.650167	3.223461	-2.194926
40	1	0	1.343562	3.796716	-0.662715
41	1	0	3.674581	0.989271	1.907805
42	1	0	2.395842	1.491287	3.060390
43	1	0	3.375340	0.045324	3.391911
44	1	0	-1.607353	-1.572404	2.296149

2-S2

Center Number	Atomic Number	Atomic Type	Coordinates (Angstroms) X	Y	Z
1	6	0	2.746035	1.663161	-0.736387
2	6	0	2.780877	0.135247	-0.949451
3	6	0	2.676615	-0.717402	0.308844
4	6	0	1.526265	-1.578320	0.629853
5	6	0	0.134266	2.158506	-0.185916
6	6	0	1.592277	2.289389	0.041083
7	6	0	0.256253	-1.843791	-0.195201
8	6	0	-0.875637	-0.890119	0.092431
9	6	0	-1.406944	0.171347	-0.881548
10	6	0	-0.425352	1.281472	-1.298623
11	6	0	-1.615944	-0.877286	1.211862
12	6	0	-2.643730	0.185062	1.081702
13	8	0	-2.510743	0.769823	-0.149815
14	8	0	0.793441	1.469017	0.909213
15	8	0	2.709092	-2.161554	0.052911
16	6	0	3.578652	-0.311559	1.458766
17	8	0	-3.484816	0.523901	1.878385
18	6	0	-1.539869	-1.705329	2.455011
19	8	0	-1.884116	-0.332098	-2.093628
20	6	0	-0.744143	3.317766	0.253841
21	6	0	-2.775308	-1.440851	-2.035848
22	8	0	0.533452	-1.907883	-1.581922
23	1	0	2.800945	2.147860	-1.721254

24	1	0	3.667423	1.958117	-0.218707
25	1	0	3.755789	-0.095304	-1.402342
26	1	0	2.028094	-0.187012	-1.665783
27	1	0	1.380294	-1.812397	1.686643
28	1	0	1.887749	3.232883	0.509641
29	1	0	-0.081393	-2.840281	0.147485
30	1	0	0.361586	0.796907	-1.873753
31	1	0	-0.979716	1.915747	-1.998862
32	1	0	3.576758	-1.082364	2.234606
33	1	0	4.611209	-0.180220	1.113242
34	1	0	3.239409	0.628616	1.904783
35	1	0	-0.927543	-1.207981	3.217907
36	1	0	-2.539478	-1.831844	2.881397
37	1	0	-1.104098	-2.692464	2.266394
38	1	0	-1.729879	2.970022	0.571547
39	1	0	-0.276653	3.855489	1.084083
40	1	0	-0.888225	4.019389	-0.577048
41	1	0	-2.261062	-2.354395	-1.714320
42	1	0	-3.628264	-1.240002	-1.377351
43	1	0	-3.138194	-1.577871	-3.056578
44	1	0	1.408896	-2.336306	-1.635399

2-S3

Center Number	Atomic Number	Atomic Type	Coordinates (Angstroms) X	Y	Z
1	6	0	-2.583822	1.978234	0.221448
2	6	0	-2.773206	0.600209	0.892389
3	6	0	-2.762460	-0.604974	-0.039687
4	6	0	-1.706477	-1.632959	-0.023831
5	6	0	0.043660	2.033341	-0.499058
6	6	0	-1.408656	2.227640	-0.720509
7	6	0	-0.428047	-1.710305	0.817730
8	6	0	0.748871	-1.057242	0.122185
9	6	0	1.439094	0.184491	0.682807
10	6	0	0.587098	1.462683	0.807168
11	6	0	1.441338	-1.495519	-0.939138
12	6	0	2.576494	-0.570690	-1.170764
13	8	0	2.534693	0.421970	-0.206518
14	8	0	-0.717989	1.119612	-1.324156
15	8	0	-2.943430	-1.878488	0.640678
16	6	0	-3.604412	-0.483443	-1.296969
17	8	0	3.431306	-0.602319	-2.017651
18	6	0	1.246021	-2.690346	-1.817043
19	8	0	1.941340	-0.226997	1.952225
20	6	0	0.995086	2.927093	-1.277345
21	6	0	2.865038	0.645955	2.609403
22	8	0	-0.640165	-1.203481	2.123628
23	1	0	-2.556286	2.745611	1.008394

24	1	0	-3.487431	2.199596	-0.360630
25	1	0	-3.766136	0.621549	1.363831
26	1	0	-2.063849	0.426734	1.698958
27	1	0	-1.576458	-2.195998	-0.953228
28	1	0	-1.639056	3.016078	-1.443494
29	1	0	-0.201995	-2.790624	0.882037
30	1	0	-0.215559	1.231706	1.506592
31	1	0	1.209099	2.236038	1.271399
32	1	0	-3.667153	-1.449995	-1.805382
33	1	0	-4.625006	-0.167417	-1.047221
34	1	0	-3.176198	0.245140	-1.992543
35	1	0	0.694173	-2.420696	-2.726225
36	1	0	2.215740	-3.084347	-2.136367
37	1	0	0.688398	-3.483338	-1.308360
38	1	0	1.929303	2.407827	-1.504690
39	1	0	0.533696	3.243962	-2.217156
40	1	0	1.240350	3.823045	-0.693292
41	1	0	3.651160	0.967157	1.920369
42	1	0	2.366485	1.521971	3.042108
43	1	0	3.306112	0.055795	3.415321
44	1	0	0.240855	-1.077185	2.521226

Table S7. Coordinates of the conformers 3-1 – 3-5.

3-1

Center Number	Atomic Number	Atomic Type	Coordinates (Angstroms) X	Y	Z
1	6	0	-1.566805	-2.289575	1.643963
2	6	0	-1.589399	-1.813403	0.204391
3	6	0	-2.921378	-1.243203	-0.269624
4	6	0	-3.213521	0.180233	0.245954
5	6	0	-2.532259	1.280135	-0.544396
6	6	0	-1.210074	1.894506	-0.272832
7	8	0	-2.435676	2.554934	0.109597
8	6	0	-0.478395	2.590548	-1.404309
9	6	0	-0.344980	1.468500	0.923810
10	6	0	1.054961	1.033387	0.603107
11	6	0	1.677582	-0.125572	0.210788
12	6	0	3.094531	0.175034	0.124158
13	6	0	4.235269	-0.732115	-0.235330
14	6	0	3.203541	1.488793	0.455848
15	8	0	1.987158	2.027662	0.749058
16	6	0	1.058164	-1.479756	-0.028911
17	8	0	1.832699	-2.154147	-1.026166
18	6	0	-0.371106	-1.425087	-0.537662
19	8	0	-0.970285	-2.722384	-0.737301
20	1	0	-2.881968	1.345810	-1.580351
21	1	0	1.090305	-2.067912	0.901399
22	1	0	-0.492289	-0.760639	-1.395219

23	1	0	-0.594794	-2.696584	1.928475
24	1	0	-1.818543	-1.480420	2.338703
25	1	0	-2.313459	-3.082057	1.773481
26	1	0	-2.929583	-1.255808	-1.366318
27	1	0	-3.722885	-1.919700	0.054199
28	1	0	-4.293374	0.368218	0.183813
29	1	0	-2.959224	0.275590	1.306801
30	1	0	0.082118	3.451245	-1.022319
31	1	0	0.235804	1.918438	-1.891407
32	1	0	-1.193588	2.949513	-2.149982
33	1	0	-0.858150	0.698974	1.505583
34	1	0	-0.260562	2.344622	1.577715
35	1	0	4.252212	-1.628089	0.394889
36	1	0	5.189732	-0.209554	-0.108094
37	1	0	4.161222	-1.078927	-1.269623
38	1	0	4.047358	2.158542	0.532953
39	1	0	1.371715	-2.994319	-1.189942

3-2

Center Number	Atomic Number	Atomic Type	Coordinates (Angstroms) X	Y	Z
1	6	0	-1.942659	3.391751	-0.487183
2	6	0	-1.440708	1.961704	-0.437188
3	6	0	-0.033541	1.739284	-0.974780
4	6	0	1.075048	2.101715	0.039071
5	6	0	2.383767	1.418526	-0.293986
6	6	0	2.763448	0.064324	0.162309
7	8	0	3.339142	1.244037	0.767526
8	6	0	3.742560	-0.749592	-0.658831
9	6	0	1.851241	-0.752241	1.092770
10	6	0	0.686635	-1.399546	0.409923
11	6	0	-0.662450	-1.151931	0.391462
12	6	0	-1.248991	-2.107781	-0.529559
13	6	0	-2.693277	-2.256733	-0.905080
14	6	0	-0.204864	-2.845319	-0.989740
15	8	0	0.978425	-2.433088	-0.436495
16	6	0	-1.400643	-0.151597	1.237732
17	8	0	-2.475219	-0.783304	1.956256
18	6	0	-2.059631	1.034988	0.535269
19	8	0	-2.421592	0.977935	-0.849125
20	1	0	2.836309	1.750072	-1.233364
21	1	0	-0.696406	0.280738	1.968142
22	1	0	-2.842664	1.449754	1.174914
23	1	0	-2.956796	3.467226	-0.084411

24	1	0	-1.289420	4.057356	0.090133
25	1	0	-1.957254	3.751542	-1.523027
26	1	0	0.055960	0.689658	-1.259686
27	1	0	0.091565	2.326138	-1.894623
28	1	0	1.236700	3.187187	0.067140
29	1	0	0.772452	1.816476	1.050872
30	1	0	4.423915	-1.298705	0.002581
31	1	0	3.217244	-1.480123	-1.281860
32	1	0	4.343338	-0.098425	-1.300600
33	1	0	1.485073	-0.097936	1.887199
34	1	0	2.465215	-1.522507	1.579480
35	1	0	-3.317684	-2.417930	-0.020503
36	1	0	-2.833356	-3.099666	-1.589880
37	1	0	-3.058365	-1.346731	-1.391644
38	1	0	-0.134603	-3.671947	-1.681056
39	1	0	-2.102882	-1.572126	2.381769

3-3

Center Number	Atomic Number	Atomic Type	Coordinates (Angstroms) X	Y	Z
1	6	0	-1.231548	-2.187267	1.776809
2	6	0	-1.480417	-1.827183	0.324877
3	6	0	-2.892920	-1.354135	-0.006499
4	6	0	-3.208447	0.095380	0.405046
5	6	0	-2.606397	1.138856	-0.514816
6	6	0	-1.293330	1.808157	-0.354298
7	8	0	-2.522151	2.472567	0.010307
8	6	0	-0.616075	2.419733	-1.564866
9	6	0	-0.389496	1.519275	0.850382
10	6	0	1.014590	1.066695	0.568242
11	6	0	1.649644	-0.078583	0.148880
12	6	0	3.075889	0.193874	0.219971
13	6	0	4.217769	-0.733148	-0.080089
14	6	0	3.180306	1.476200	0.654907
15	8	0	1.951474	2.022291	0.869408
16	6	0	1.100752	-1.413232	-0.304812
17	8	0	1.796748	-1.872040	-1.476130
18	6	0	-0.387028	-1.462221	-0.604414
19	8	0	-0.959512	-2.775278	-0.629659
20	1	0	-2.999343	1.095526	-1.536273
21	1	0	1.327909	-2.182183	0.440779
22	1	0	-0.663937	-0.850839	-1.469136
23	1	0	-0.198840	-2.495478	1.952427

24	1	0	-1.457549	-1.346994	2.443266
25	1	0	-1.884364	-3.021042	2.061561
26	1	0	-3.042028	-1.476112	-1.086404
27	1	0	-3.606085	-2.031744	0.481066
28	1	0	-4.296462	0.242021	0.386905
29	1	0	-2.902843	0.288729	1.438595
30	1	0	-0.079878	3.332123	-1.278521
31	1	0	0.113174	1.732790	-2.006921
32	1	0	-1.359965	2.683056	-2.322181
33	1	0	-0.883941	0.815861	1.525028
34	1	0	-0.298050	2.460293	1.404818
35	1	0	4.144021	-1.659313	0.502556
36	1	0	5.172473	-0.257117	0.167606
37	1	0	4.238665	-1.031739	-1.131824
38	1	0	4.024485	2.117188	0.862580
39	1	0	1.720345	-1.169751	-2.143261

3-4

Center Number	Atomic Number	Atomic Type	Coordinates (Angstroms) X	Y	Z
1	6	0	-1.887064	3.402510	-0.504706
2	6	0	-1.416772	1.961594	-0.448393
3	6	0	-0.025479	1.705592	-1.008807
4	6	0	1.090973	2.082545	-0.008477
5	6	0	2.398668	1.385884	-0.315339
6	6	0	2.768609	0.044636	0.184552
7	8	0	3.338696	1.239718	0.764177
8	6	0	3.760559	-0.791995	-0.597603
9	6	0	1.834857	-0.744038	1.117770
10	6	0	0.674521	-1.387876	0.425264
11	6	0	-0.674888	-1.151026	0.407222
12	6	0	-1.245059	-2.097979	-0.532074
13	6	0	-2.686341	-2.278082	-0.908947
14	6	0	-0.194003	-2.818260	-1.003164
15	8	0	0.979882	-2.405506	-0.439148
16	6	0	-1.425978	-0.157677	1.259083
17	8	0	-2.464885	-0.799920	2.015300
18	6	0	-2.025775	1.069373	0.562519
19	8	0	-2.438597	1.000988	-0.813371
20	1	0	2.863441	1.692121	-1.257282
21	1	0	-0.749550	0.225520	2.031982
22	1	0	-2.772444	1.527101	1.217821
23	1	0	-2.887320	3.507860	-0.074784

24	1	0	-1.202463	4.060651	0.043661
25	1	0	-1.922819	3.747124	-1.545148
26	1	0	0.049829	0.649047	-1.270006
27	1	0	0.094500	2.272164	-1.941795
28	1	0	1.259068	3.167421	-0.006997
29	1	0	0.785928	1.824859	1.010123
30	1	0	4.375163	-0.157823	-1.243385
31	1	0	4.427802	-1.325284	0.090572
32	1	0	3.245554	-1.536751	-1.211965
33	1	0	1.463956	-0.073910	1.896502
34	1	0	2.431438	-1.513041	1.627506
35	1	0	-3.275092	-2.691654	-0.078261
36	1	0	-2.785840	-2.976629	-1.746119
37	1	0	-3.134737	-1.325554	-1.210272
38	1	0	-0.116345	-3.632173	-1.708741
39	1	0	-3.151809	-1.074493	1.387522

3-5

Center Number	Atomic Number	Atomic Type	Coordinates (Angstroms) X	Y	Z
1	6	0	2.714135	-1.991705	-1.563236
2	6	0	1.922677	-1.641006	-0.312512
3	6	0	2.612187	-0.650055	0.618534
4	6	0	2.849646	0.760150	0.019827
5	6	0	1.948395	1.821435	0.613594
6	6	0	0.589985	2.168379	0.151873
7	8	0	1.726239	2.995009	-0.187890
8	6	0	-0.374690	2.811460	1.127876
9	6	0	-0.046711	1.476386	-1.059280
10	6	0	-1.292540	0.707915	-0.747509
11	6	0	-1.590828	-0.461584	-0.096494
12	6	0	-3.035033	-0.566266	-0.081036
13	6	0	-3.860445	-1.661738	0.525404
14	6	0	-3.490580	0.540291	-0.724246
15	8	0	-2.451092	1.324236	-1.133385
16	6	0	-0.674697	-1.464362	0.547511
17	8	0	-0.241545	-0.986655	1.821955
18	6	0	0.471214	-1.955699	-0.331743
19	8	0	1.396914	-2.824205	0.347169
20	1	0	2.163043	2.041427	1.663826
21	1	0	-1.276086	-2.375785	0.707064
22	1	0	0.093470	-2.368646	-1.272303
23	1	0	2.213819	-2.783632	-2.127576

24	1	0	2.840379	-1.126429	-2.222954
25	1	0	3.712486	-2.350058	-1.285209
26	1	0	2.039445	-0.562530	1.542125
27	1	0	3.576728	-1.104102	0.880871
28	1	0	3.884821	1.071747	0.206263
29	1	0	2.737909	0.762641	-1.068184
30	1	0	-1.073684	3.473667	0.603568
31	1	0	-0.954142	2.049137	1.657796
32	1	0	0.178342	3.407127	1.859823
33	1	0	0.686502	0.834503	-1.554609
34	1	0	-0.317781	2.257008	-1.779708
35	1	0	-3.667477	-2.632190	0.050524
36	1	0	-4.928932	-1.449906	0.417046
37	1	0	-3.649949	-1.776541	1.595627
38	1	0	-4.478248	0.900950	-0.970096
39	1	0	0.318941	-1.697083	2.181932
